# Expression of Rate-Limiting Enzymes of Melatonin Synthesis in Several Extrapineal Organs During Pregnancy in Ewes

**DOI:** 10.3390/biom16071047

**Published:** 2026-07-17

**Authors:** Yanshu Xu, Haozhe Hou, Yang Yang, Dongsheng Gao, Leying Zhang, Ling Yang

**Affiliations:** School of Life Sciences and Food Engineering, Hebei University of Engineering, Handan 056038, China; 18831709263@163.com (Y.X.); 19831375921@163.com (H.H.); jiaoyang79@163.com (Y.Y.); 15512753002@163.com (D.G.); zhangly056000@126.com (L.Z.)

**Keywords:** aralkylamine N-acetyltransferase, acetylserotonin O-methyltransferase, extrapineal organs, pregnancy, small-tail Han ewes

## Abstract

Melatonin is not only produced by the pineal gland but is also synthesized in extrapineal organs. It plays a key role in antioxidant defense and immune regulation during pregnancy. Two key rate-limiting enzymes are involved in the melatonin synthesis, aralkylamine N-acetyltransferase (AANAT), and acetylserotonin O-methyltransferase (ASMT). Nevertheless, it remained unclear whether pregnancy affects the expression of AANAT and ASMT in extrapineal organs. In this study, ovine maternal thymus, spleen, liver, lymph nodes, thyroid, duodenum, and endometrium were collected on day 16 of the estrous cycle (N16), and on days 13, 16, 25, and 70 (G70) of gestation (*n* = 6 per group). The expression of AANAT and ASMT in these extrapineal organs was analyzed using RT-qPCR, Western blot, and immunohistochemistry. The results revealed that pregnancy upregulated the expression of AANAT in the liver and ASMT in the thymus and duodenum, but downregulated AANAT expression in the lymph nodes and duodenum. Moreover, AANAT expression was elevated in the thymus and spleen but reduced in the thyroid and endometrium at G70 compared with N16. ASMT expression was increased in the lymph nodes yet decreased in the spleen, liver, and thyroid at G70 compared with N16. Notably, endometrial ASMT expression showed a pregnancy-stage-specific manner with no significant difference between N16 and G70. In summary, this paper reports, for the first time in sheep, that pregnancy modulates the expression of AANAT and ASMT in these extrapineal organs in a pregnancy-stage- and tissue-specific manner.

## 1. Introduction

Melatonin maintains regular circadian rhythms and optimal immune regulation during pregnancy, which are critical for uterine receptivity, fetal growth, and development [1]. Early pregnancy modulates the expression of melatonin receptors and CD4 in the ovine maternal thymus, lymph node, spleen, and liver in a tissue-specific manner, which participates in the immune regulation of these organs in sheep [2]. Melatonin supplementation can reinstate gut microbiota in heat-stressed pregnant mice, and also enhance intestinal and placental barrier integrity to protect fetuses against oxidative stress [3]. Maternal circadian disruption and elevated oxidative stress trigger reproductive disorders during pregnancy, which can be alleviated by melatonin to improve pregnancy outcomes [4].

The pineal gland, gastrointestinal tract, liver, thymus, placenta, and uterus can synthesize melatonin from tryptophan, and aralkylamine N-acetyltransferase (AANAT) and acetylserotonin O-methyltransferase (ASMT) are two key rate-limiting enzymes in the melatonin biosynthesis [5]. AANAT protein is expressed in rat thyroid cells, where it has paracrine and autocrine functions and safeguards follicular cells from oxidative stress [6]. There is an upregulation of AANAT and ASMT in the corpus luteum during pregnancy, and melatonin plays a paracrine and/or autocrine role in luteal function of pregnant sows [7]. AANAT is expressed in the uterus, which participates in the formation of endometrial receptivity via melatonin membrane receptor 2 (MT2) in mice [8].

A successful pregnancy involves complex immune tolerance mechanisms that play critical roles in enabling the fetus to evade rejection by the maternal immune system [9]. The ruminant pregnancy recognition signal (interferon-tau, IFNT) secreted by the conceptus communicates with the maternal immune system to modulate the maternal immune tolerance during pregnancy, which plays an important role in success of pregnancy [10]. In addition, IFNT stimulates the mRNA and protein expression of interferon-stimulated gene 15 in maternal thymus, spleen, liver, and lymph nodes, which is implicated in the immunoregulation of ewes during early pregnancy [11]. Furthermore, the expression of interferon-stimulated genes in the thyroid and duodenum changes in a pregnancy-stage- and tissue-specific manner during early gestation in ewes [12,13].

In this study, it was hypothesized that the expression of rate-limiting enzymes (AANAT and ASMT) of melatonin synthesis in the maternal thymus, spleen, liver, lymph node, thyroid, duodenum, and endometrium was modulated during pregnancy. The objective of this study was to characterize expression profiles of AANAT and ASMT in these maternal organs from nonpregnant and pregnant ewes at different stages, which will help to elucidate the effects of pregnancy stage on the expression of these rate-limiting enzymes in the maternal main immune organs, thyroid, duodenum, and endometrium.

## 2. Materials and Methods

### 2.1. Animals and Experimental Design

This study was carried out on a farm of small-tail Han ewes from October to December, during the short-day season with natural lighting, and all experimental procedures involving animals were approved by the Hebei University of Engineering Animal Care and Use Committee (HUEAE 2019-017). Thirty multiparous ewes, aged 18 months, with similar body conditions (average weight of 41 kg and good reproductive function) and body condition score of 3 ± 0.5 described by Ngwa et al. [14], were randomly divided into 5 groups (*n* = 6 per group). After estrous synchronization, an epididymectomized ram was used to detect sexual receptivity (day 0 of pregnancy or nonpregnancy). The females in four groups were bred with intact males, but animals of the remaining group were not. All ewes were euthanized between 11:00 and 12:00 a.m. on days 13 (G13), 16 (G16), 25 (G25), and 70 (G70) of gestation, and on day 16 of the estrous cycle (N16). Immediately before sampling after euthanasia, it was confirmed that all ewes in the pregnant groups carried a viable conceptus in the uterus. The thymus, spleen, liver, lymph nodes, thyroid, duodenum, and endometrium were sampled, snap-frozen in liquid nitrogen, and stored at −80 °C for RNA extraction and Western blot analysis. In addition, cross-sections of these tissues were immediately fixed in fresh 4% (*w*/*v*) paraformaldehyde for subsequent immunohistochemistry staining.

### 2.2. RNA Extraction and RT-qPCR Assay

The frozen samples of these tissues were crushed into fine pieces in liquid nitrogen and subjected to the TRNzol Universal (Tiangen Biotech Co., Ltd., Beijing, China) extraction protocol for RNA extraction. The RNA purity was evaluated by agarose gel (1%) electrophoresis, and optical density at 260/280 nm was in the range of 1.8 and 2.1. RNA integrity was assessed by examining the 28S and 18S rRNA bands of representative samples. Genomic DNA removal and cDNA synthesis were performed using a FastQuant RT kit with gDNase (KR106, Tiangen Biotech) according to the manufacturer’s instructions. The ovine-specific primers for *AANAT* and *ASMT* genes are listed in Table 1, and ovine glyceraldehyde-3-phosphate dehydrogenase (*GAPDH*) was employed to normalize the relative expression. The cDNA templates were amplified utilizing a SuperReal PreMix Plus kit (Tiangen Biotech Co., Ltd.) in a CFX96 real-time PCR system (Bio-Rad Laboratories, Inc., Hercules, CA, USA) according to the manufacturer’s recommendations. A template that did not undergo reverse transcription reaction served as the negative control, and water was used instead of DNA template as a no-template control. Amplification of a single product by a dissociation curve was used to confirm the specificity of the primers, and the amplification efficiency ranged from 95 to 105% (Figure S2). It has been reported that *GAPDH* is a suitable reference gene in sheep, as determined using the geNorm algorithm [15]. The 2^−ΔΔCt^ method was used to analyze gene expression [16], and *GAPDH* was used to normalize the relative expression. The samples from N16 were set to a value of 1.

### 2.3. Western Blot

Total protein extraction and concentration determination were carried out as described previously [11]. A pre-stained molecular weight ladder and 10 µg of total proteins were separated by electrophoresis, and the separated proteins were electrotransferred onto polyvinylidene fluoride membrane (Millipore, Bedford, MA, USA). Skim milk powder (5%) was utilized to block the blot at 4 °C overnight, and primary antibodies (Table 2) were used to detect the target proteins with a dilution of 1:1000. The membrane was incubated with goat anti-rabbit IgG-HRP (Biosharp, Hefei, China; BL003A) secondary antibody at a dilution of 1:2000 for 1 h at room temperature after being washed 3 times. The protein signals were detected using a chemiluminescence Western blotting detection reagent (Tiangen Biotech). In addition, GAPDH antibody (Santa Cruz Biotechnology, Santa Cruz, CA, USA; sc-47724) was used to normalize relative expression on a parallel membrane using the same protein extracts and concentration. Semi-quantitative analysis was performed utilizing the Quantity One V452 software (Bio-Rad Laboratories). AANAT may show two protein bands (23 kDa and 35 kDa), and ASMT protein may have two bands (near 38 kDa) caused by different splicing isoforms [7,17]. When multiple bands were detected, all bands corresponding to AANAT or ASMT were summed for densitometric analysis.

### 2.4. Immunohistochemistry Analysis

Immunohistochemistry analysis for AANAT protein in the thymus, spleen, liver, lymph nodes, thyroid, duodenum, and endometrium was performed as described previously [11]. The tissues of these organs were dehydrated in ethanol, embedded in paraffin, and then cut into 5 μm thick sections, following deparaffinization, rehydration, and antigen recovery. Tissue sections were incubated with the rabbit polyclonal antibody to AANAT (ABclonal Biotechnology Co., Ltd., Wuhan, Hubei, China, A11850) at a 1:100 dilution overnight at 4 °C for detecting the target protein. In contrast, for the negative controls, the AANAT antibody was replaced with an antiserum-specific isotype at the same protein concentration. A goat anti-rabbit IgG-HRP secondary antibody (Biosharp, BL003A) was utilized at a dilution of 1:500 to recognize the primary antibody, and hematoxylin was used for nuclear stain. Images were captured using a light microscope (Nikon Eclipse E800, Tokyo, Japan) to analyze the localization of AANAT protein in these tissues.

### 2.5. Statistical Analysis

Statistical analysis was performed using the General Linear Model procedures of SAS (Version 9.3; SAS Institute, Cary, NC, USA). Data for relative expression values of mRNA and protein for AANAT and ASMT in the thymus, spleen, liver, lymph nodes, thyroid, duodenum, and endometrium were subjected to ANOVA. Data from these tissues were analyzed across five experimental groups (N16, G13, G16, G25, and G70), with individual ewes serving as the experimental unit. Data normality was tested using the PROC UNIVARIATE procedure in SAS version 9.2 (SAS Institute Inc.), and the data were normally distributed. One-way ANOVA followed by Tukey’s post hoc test was used to analyze differences in the relative expression levels of mRNA and protein across groups. All results were presented as the least squares means ± SEM. Statistical significance was determined at *p* < 0.05.

## 3. Results

### 3.1. Expression Levels of AANAT and ASMT in the Thymus

**Figure 1:**
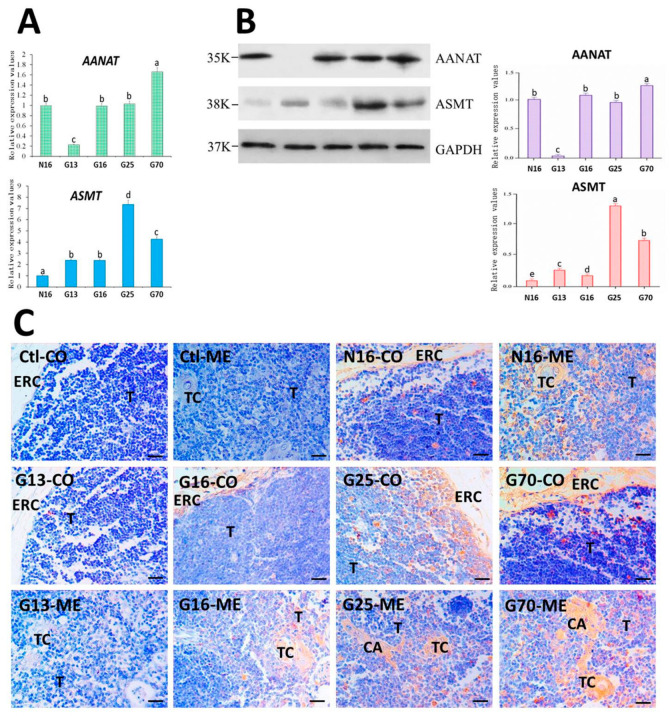
Expression of AANAT and ASMT in the thymus. (**A**) Expression values of *AANAT* and *ASMT* mRNA in the thymus. (**B**) Expression of AANAT and ASMT proteins in one representative animal per group. (**C**) Representative immunohistochemical localization of AANAT protein in the thymus. The thymus is divided into the cortex (CO) and the medulla (ME). Note: Significant differences (*p* < 0.05) are indicated by different letters. T = thymocyte; ERC = epithelial reticular cell; CA = capillary; TC = thymic corpuscle. Bar = 20 µm.

As shown in Figure 1A,B and Figure S1, the relative expression levels of *AANAT* mRNA and protein were the highest in the thymus at G70 and the lowest at G13 among the five groups (*p* < 0.05). There was no significant difference among N16, G16, and G25 (*p* > 0.05), but AANAT protein was almost undetectable at G13. In addition, the mRNA and protein levels of ASMT peaked at G25, with the lowest values at N16 (*p* < 0.05). Furthermore, ASMT levels at G70 were higher than those at G13 and G16 (*p* < 0.05). AANAT protein was mainly localized to the epithelial reticular cells, capillaries, and thymic corpuscles (Figure 1C).

### 3.2. Expression Levels of AANAT and ASMT in the Lymph Nodes

**Figure 2:**
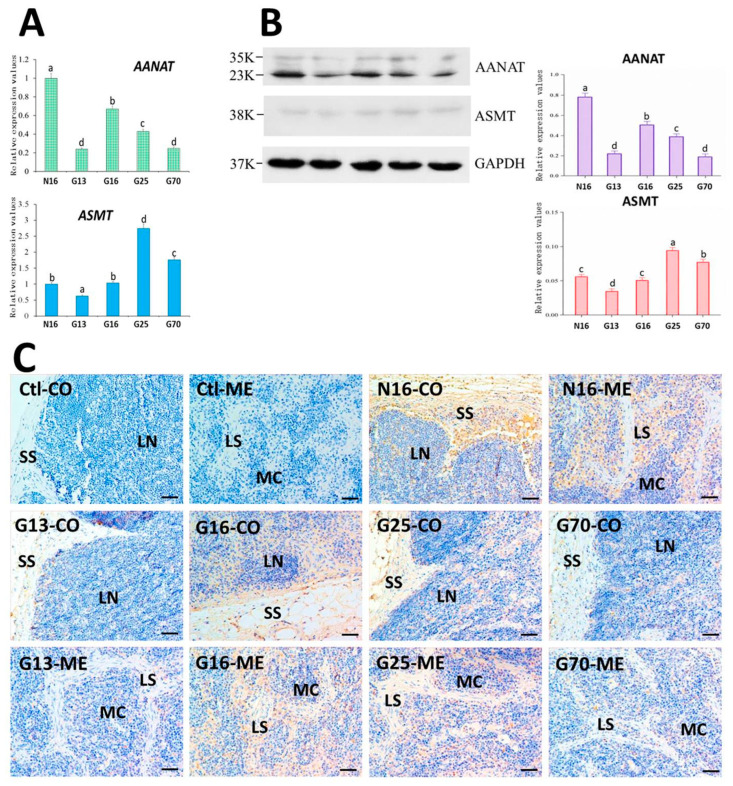
Expression of AANAT and ASMT in the lymph node. (**A**) Expression values of *AANAT* and *ASMT* mRNA in the lymph node. (**B**) Expression of AANAT and ASMT proteins in one representative animal per group. (**C**) Representative immunohistochemical localization of AANAT protein in the lymph node. The lymph node is divided into an outer cortex (CO) and an inner medulla (ME). Lymph enters the convex through the subcapsular sinus (SS) around the lymphoid nodule (LN) and flows into the medulla through the lymph sinus (LS) around the medullary cord (MC). Significant differences (*p* < 0.05) are indicated by different letters. Bar = 20 µm.

Pregnancy downregulated the mRNA and protein expression of AANAT in the lymph nodes compared with N16 (*p* < 0.05), and among the pregnant ewes, the values were the highest at G16 (Figure 2A,B and Figure S1). There were two protein bands (23 kDa and 35 kDa) of AANAT. In addition, the levels of *ASMT* mRNA and protein were the highest at G25, with the lowest value at G13, among the five groups (*p* < 0.05). The values of ASMT expression were greater at G70 than at N16 and G16 (*p* < 0.05). Furthermore, the AANAT protein was mainly located in the subcapsular sinus and lymph sinus (Figure 2C).

### 3.3. Expression Levels of AANAT and ASMT in the Spleen

**Figure 3:**
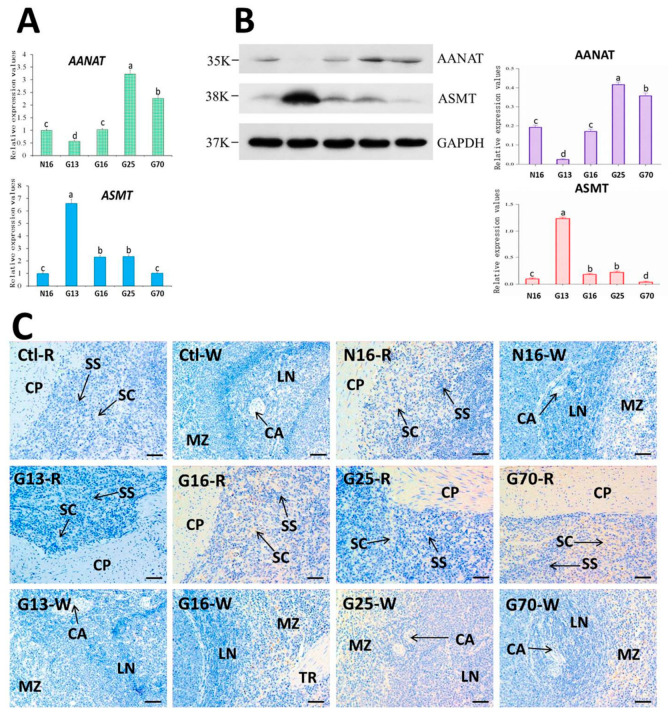
Expression of AANAT and ASMT in the spleen. (**A**) Expression values of *AANAT* and *ASMT* mRNA in the spleen. (**B**) Expression of AANAT and ASMT proteins in one representative animal per group. (**C**) Representative immunohistochemical localization of AANAT protein in the spleen. Spleen is divided into red pulp (R) and white pulp (W), and surrounded by a thickened capsule (CP). Capsule with several trabeculae (TR) projects into the substance of the spleen. Note: Significant differences (*p* < 0.05) are indicated by different letters. Ctl = negative control; SS = splenic sinuses; SC = splenic cords; MZ = marginal zone; LN = lymphoid nodule; CA = central arteriole. Bar = 50 µm.

There was an upregulation of the relative expression levels of *AANAT* mRNA and protein at G25 and G70 in the spleen compared with N16, G13, and G16 (*p* < 0.05), and AANAT protein was almost undetected at G13 (Figure 3A,B and Figure S1). However, the relative level of *ASMT* mRNA and protein peaked at G13, with the lowest values at G70 (*p* < 0.05). In addition, the immunohistochemistry for the AANAT protein was limited to the capsule, trabeculae, and splenic cords (Figure 3C).

### 3.4. Expression Levels of AANAT and ASMT in the Liver

**Figure 4:**
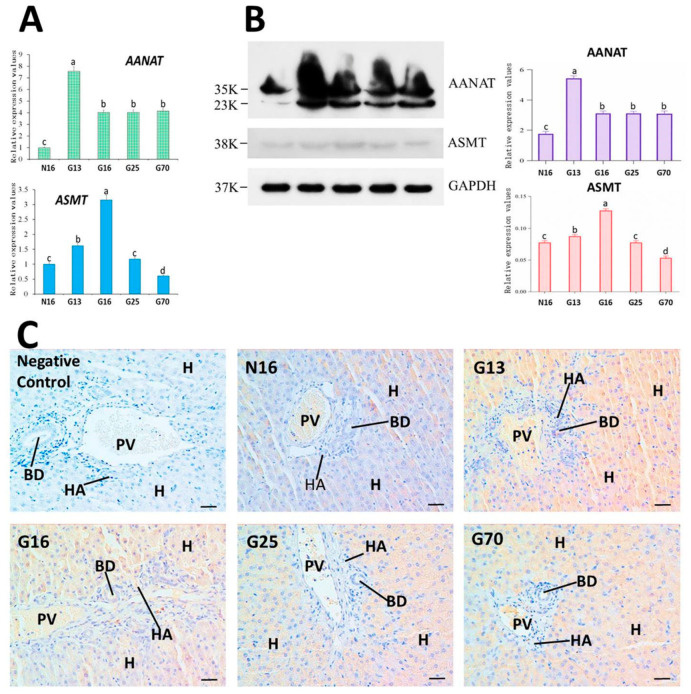
Expression of AANAT and ASMT in the liver. (**A**) Expression values of *AANAT* and *ASMT* mRNA in the liver. (**B**) Expression of AANAT and ASMT proteins in one representative animal per group. (**C**) Representative immunohistochemical localization of AANAT protein in the liver. A portal triad is a component of the hepatic lobule and consists of proper hepatic artery (HA), hepatic portal vein (PV), and small bile ductile (BD). Note: Significant differences (*p* < 0.05) are indicated by different letters. H = hepatocyte; Bar = 50 µm.

The expression values of *AANAT* mRNA and protein peaked at G13, with the lowest levels at N16 (*p* < 0.05), and there was no significant difference among G16, G25, and G70 (*p* > 0.05) (Figure 4A,B and Figure S1). In addition, AANAT presented as two protein bands (23 kDa and 35 kDa). However, ASMT protein expression was weak in the maternal liver. The expression levels of *ASMT* mRNA and protein peaked at G16, with the lowest values at G70, and ASMT levels were greater at G13 than that at N16 and G25 (*p* < 0.05). Furthermore, the immunohistochemistry for the AANAT protein was localized to hepatocytes (Figure 4C).

### 3.5. Expression Levels of AANAT and ASMT in the Thyroid

**Figure 5:**
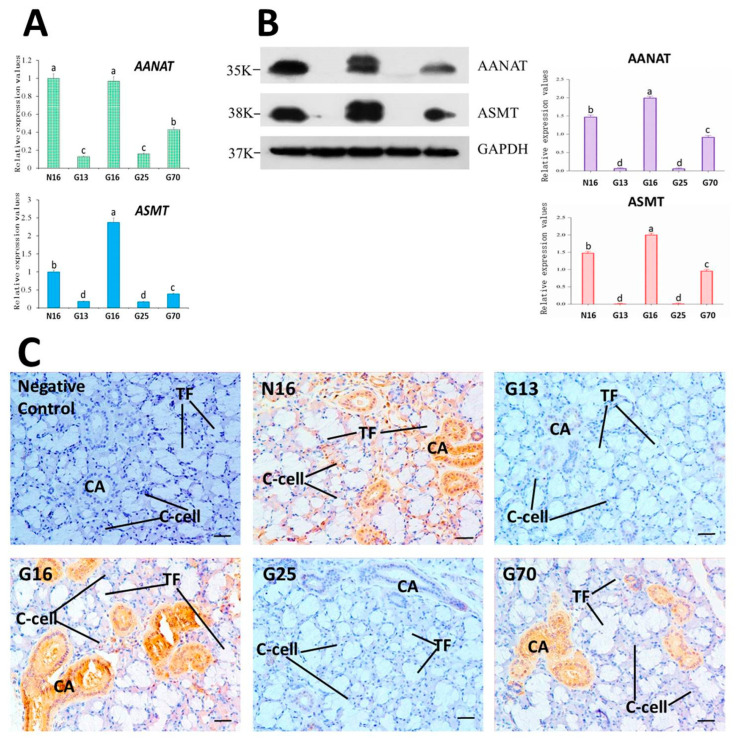
Expression of AANAT and ASMT in the thyroid. (**A**) Expression values of *AANAT* and *ASMT* mRNA in the thyroid. (**B**) Expression of AANAT and ASMT proteins in one representative animal per group. (**C**) Representative immunohistochemical localization of AANAT protein in the thyroid. Thyroid follicles (TF), containing colloid in their lumina, are lined predominantly by parafollicular cells (C-cells). Significant differences (*p* < 0.05) are indicated by different letters. CA = capillary. Bar = 50 µm.

The mRNA and protein expression levels of both AANAT and ASMT were greater at N16 and G16 compared with other three groups (*p* < 0.05), and their values were lower at G13 and G25 than that at G70 (*p* < 0.05) (Figure 5A,B and Figure S1). In addition, AANAT and ASMT proteins were almost undetected at G13 and G25. Furthermore, the immunohistochemistry for the AANAT protein was strongly located to the capillaries and weakly to the parafollicular cells, also known as thyroid medullary cells (C-cells), and thyroid follicular cells.

### 3.6. Expression Levels of AANAT and ASMT in the Duodenum

**Figure 6:**
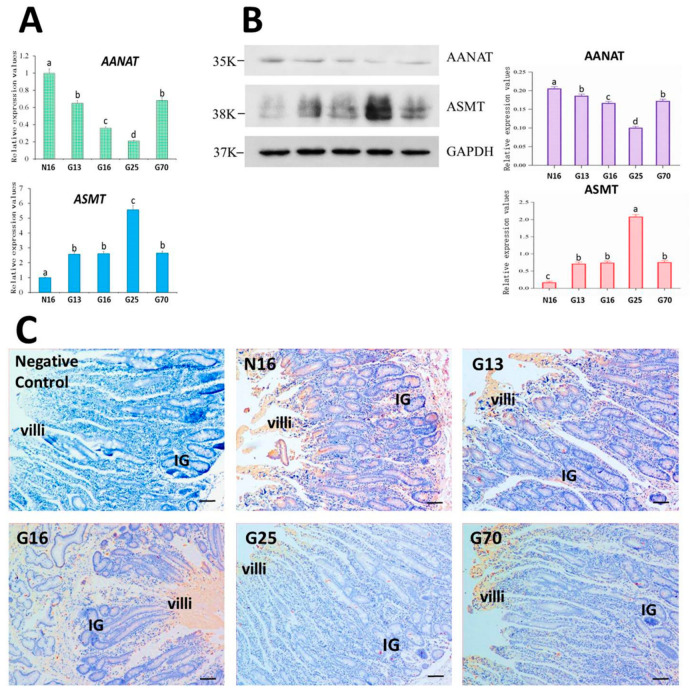
Expression of AANAT and ASMT in the duodenum. (**A**) Expression values of *AANAT* and *ASMT* mRNA in the duodenum. (**B**) Expression of AANAT and ASMT proteins in one representative animal per group. (**C**) Representative immunohistochemical localization of AANAT protein in the duodenum. The mucosa forms fingerlike projections called villi, and there are the intestinal glands (IGs) underlying the villi in the duodenum. Significant differences (*p* < 0.05) are indicated by different letters. Bar = 50 µm.

As shown in Figure 6A,B and Figure S1, the relative expression levels of *AANAT* mRNA and protein peaked in the duodenum at N16 and were the lowest at G25 (*p* < 0.05). However, the levels of *ASMT* mRNA and protein were the lowest at N16 (*p* < 0.05), with the greatest values at G25 (*p* < 0.05). ASMT protein presented as two bands (near 38 kDa). In addition, the immunohistochemistry for the AANAT protein was strongly located in villi, and weakly in intestinal glands (IGs, Figure 6C).

### 3.7. Expression Levels of AANAT and ASMT in the Endometria

**Figure 7:**
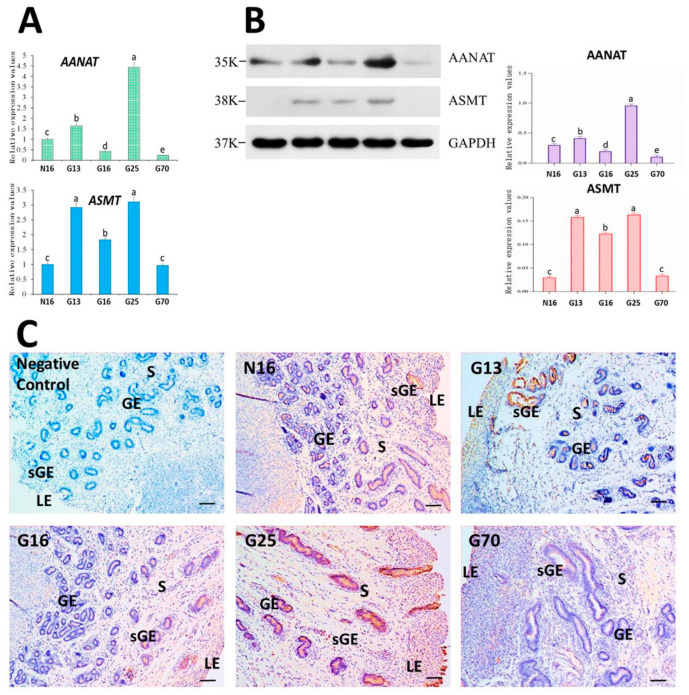
Expression of AANAT and ASMT in the endometria. (**A**) Expression values of *AANAT* and *ASMT* mRNA in the endometria. (**B**) Expression of AANAT and ASMT proteins in one representative animal per group. (**C**) Representative immunohistochemical localization of AANAT protein in the endometria. Note: sGE = superficial glandular epithelium; LE = luminal epithelium; GE = glandular epithelium; S = stroma. Significant differences (*p* < 0.05) are indicated by different letters. Bar = 50 µm.

The relative expression levels of *AANAT* mRNA and protein were downregulated in the endometrium at G70 compared with the other four stages (*p* < 0.05), with a peak at G25 (Figure 7A,B and Figure S1). The levels of AANAT were lower at G16 compared with N16 and G13 (*p* < 0.05). In addition, ASMT expression levels were greater at G13 and G25 compared with N16, G16, and G70, and the expression values at N16 and G70 were lower than that at G16. ASMT protein was almost undetected at N16 and G70. Furthermore, the immunohistochemistry for the AANAT protein was localized to the uterine luminal epithelium (LE) and superficial glandular epithelium (sGE), with weak staining in the stroma (Figure 7C).

## 4. Discussion

**Figure 8:**
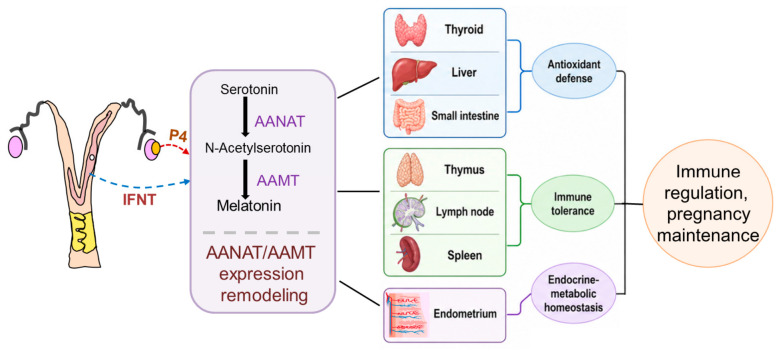
A hypothetical model of pregnancy modulating the expression of rate-limiting enzymes of melatonin synthesis, including N-acetyltransferase (AANAT) and acetylserotonin O-methyltransferase (ASMT), in the maternal thymus, spleen, liver, lymph node, thyroid, duodenum, and endometrium based on the literature. Pregnancy signal (interferon-tau, IFNT) and progesterone (P4) may induce changes in the expression of AANAT and ASMT in these maternal extrapineal organs, which may be involved in antioxidant defense, immune tolerance, and endometrial endocrine–metabolic homeostasis. Finally, these changes may contribute to maternal immune regulation and maintenance of pregnancy in sheep.

The thymus, spleen, and lymph nodes are immune organs, while the liver, thyroid, and duodenum are involved in to nutrient digestion, absorption, and metabolism. The endometrium plays a critical role during pregnancy. The maternal immune system needs to be modulated [9], and the thyroid and duodenum are also regulated in a pregnancy-stage-specific manner during early gestation in ewes [12,13]. In sheep, IFNT and progesterone have effects on the maternal thymus, spleen, liver, lymph node, thyroid, duodenum, and endometrium during pregnancy [12,13]. In this study, pregnancy modulated the expression of AANAT and ASMT in these extrapineal organs in a pregnancy-stage- and tissue-specific manner. This modulation may suggest a potential relationship with pathways involved in maternal immune, oxidative, and metabolic adaptation during pregnancy (Figure 8).

There is a thymus–pineal axis in which AANAT, expressed in both the thymus and pineal, regulates melatonin secretion, thereby affecting thymocyte maturation and immune function [18]. In addition, ASMT is expressed in the human thymus and is implicated in the immune efficiency of the thymus [19]. Furthermore, melatonin treatment at a physiological dose can regulate the proliferation and differentiation of T cells in the thymus [20]. There are adaptations in the maternal thymus for the development of T lymphocytes and central tolerance during pregnancy, and endogenous thymic melatonin synthesis is implicated in thymocyte maturation and thymic peptide production [21]. Melatonin can repair thymus damage caused by T-2 toxin through antioxidant, anti-inflammatory, and anti-apoptotic mechanisms, and restore its immune function in mice [22]. Moreover, melatonin receptors are upregulated in the maternal thymus during early pregnancy, which is related to immune regulation of the maternal thymus in ewes [2]. In this study, the data showed that AANAT protein expression peaked at G70 in the maternal thymus but was undetectable at G13. However, ASMT expression was upregulated at G25 and G70. Therefore, the upregulation of AANAT at G70 and ASMT at G25 and G70 may be associated with the modulation of the thymus function during pregnancy.

Lymph nodes consist of antigen-presenting cells and antigen-responsive cells, which are involved in the generation of adaptive immune responses and the suppression of autoreactive cells [23]. Melatonin has immunomodulatory activity, which inhibits the Th1-dependent immune response by suppressing the production of interferon-γ in the lymph nodes [24]. Melatonin injection can improve the circadian rhythm of immune cell proliferation and autonomic activity in submaxillary lymph nodes of old rats [25]. Our previous research found that early pregnancy stimulates the expression of interferon-stimulated gene 15 and modulates prostaglandin synthase expression in ovine maternal lymph nodes [26]. In addition, the expression of MT1 increases in the lymph nodes of ewes, contributing to the immune regulation of the maternal lymph nodes during early pregnancy [2]. However, there are currently no reports on AANAT and ASMT expression in lymph nodes. In this study, mRNA and protein expression of AANAT declined at G25 and G70, while ASMT expression was enhanced in the maternal lymph nodes compared with N16. Therefore, it is suggested that the modulation of AANAT and ASMT in the maternal lymph nodes may be associated with immune regulation during pregnancy.

The spleen harbors diverse populations of innate immune cells that play critical roles in regulating adaptive immune responses [27]. Melatonin can alleviate atrazine-induced immune damage in the spleen [28] and improve spleen tissue structure and splenocyte proliferation in mice [29]. In addition, AANAT and ASMT are expressed in the spleen with a circadian rhythm, which contributes to the daily oscillation of the immune system [30]. Melatonin treatment can increase the AANAT activity and modulate the inflammatory response and MT1 expression in the spleen of hamsters [31]. Furthermore, the relative expression levels of MT1 and MT2 in the spleen decrease during early pregnancy, which is related to the maternal immune tolerance in ewes [2]. In this study, AANAT expression was upregulated at G16, G25, and G70. However, the expression levels of ASMT peaked at G13, but significantly declined at G70. Therefore, it is suggested the upregulation of AANAT and changed expression of ASMT may be related to the modulation of maternal splenic function during pregnancy.

Carbohydrate and lipid metabolism is regulated in the liver by melatonin. The liver is the sole organ where circulating melatonin is metabolized into products excreted in the urine [32]. There are anatomical and immunological maternal hepatic adaptations during pregnancy, and melatonin can act on the liver to elevate plasma glucose levels and attenuate liver steatosis [33]. Melatonin enhances the activity of key antioxidant enzymes, including superoxide dismutase and catalase, helping to alleviate oxidative stress in the liver of prediabetic rats [34]. AANAT is expressed in the liver, where it is related to the regulation of hepatic microvascularization and biliary functions by melatonin via autocrine and paracrine mechanisms in rats [35]. *AANAT* and *ASMT* genes are upregulated in the liver, which is associated with the activity of superoxide dismutase and catalase in old Wistar rats [36]. MT1 is upregulated in the maternal liver, but MT2 is downregulated during early pregnancy in an animal model, which affects lipid and glucose metabolism [2]. Our data showed that pregnancy induced the expression of AANAT in the maternal liver, and ASMT expression was also increased at G16 and G25. Therefore, it is suggested that the increases in the expression of AANAT and ASMT may be associated with the antioxidant and immunoregulatory functions of melatonin in the liver during pregnancy in sheep.

Pregnancy changes maternal hormone levels and metabolic demands, thereby leading to significant alterations in maternal thyroid function [37]. AANAT is expressed in the C-cells of the rat thyroid, enabling them to synthesize melatonin autonomously and regulate follicular cell function through paracrine pathways [6]. Both thyroid follicular cells and C-cells express ASMT and AANAT, which are involved in melatonin synthesis in the thyroid [38]. The expression of AANAT and ASMT in thyroid C-cells exerts effects on thyroid function in a paracrine manner [39]. Melatonin treatment prevents oxidative damage induced by sodium/iodide symporter inhibitors, and melatonin can be used as a potential antioxidant in the thyroid [40]. Early pregnancy affects the expression of interferon-stimulated genes in the thyroid in a pregnancy-stage- and tissue- specific manner in sheep [12]. Our results showed that the expression of AANAT and ASMT was greater at N16, G16, and G70 than G13 and G25. Therefore, it is suggested that the variation in AANAT and ASMT expression may be associated with the modulation of thyroid function by melatonin.

Melatonin can regulate the duodenal circadian rhythm and the expression of lipid transport genes to improve lipid absorption and excretion, and adjust gut microbiota composition to alleviate continuous light-induced metabolic disorders in mice [41]. ASMT overexpression in melatonin-enriched transgenic sheep can enhance resistance to brucellosis by modulating immune-related signaling pathways and intestinal microbiota [42]. *AANAT* mRNA is expressed in the duodenum, but its expression is inhibited by zinc oxide nanoparticles, which have potential toxicity and reduce the melatonin synthesis and secretion in mice [43]. The metabolites of the intestinal microbiota integrate circadian signals to induce melatonin biosynthesis, which is related to the expression of AANAT and ASMT in the gut [44]. Our previous research has reported that early pregnancy modulates the duodenal adaptation and immunoregulation via regulating the expression of progesterone receptors, signal transducer, and activator of transcription protein 1, which is related to IFNT and progesterone [13]. In this study, there was a downregulation of AANAT at G16 and G25, but upregulation at G70. In addition, ASMT expression upregulated during pregnancy. Therefore, the decrease in AANAT at G16 and G25 may be related to pregnancy recognition and embryo implantation. However, the upregulation of AANAT at G70 and ASMT during pregnancy may be associated with the modulation of duodenal function.

AANAT is a key enzyme for melatonin synthesis and is expressed in the epithelial cells of the endometrium of sheep [45]. AANAT can promote endometrial receptivity and embryo implantation, and AANAT absence leads to abnormal endometrium and implantation failure in mice [8]. Upregulation of AANAT boosts melatonin synthesis and enhances endometrial and gland development, thereby improving key implantation gene expression and enhancing implantation rate and litter size during early pregnancy in mice [46]. In addition, the expression level of the *ASMT* gene reaches its peak on day 10 of the estrus cycle, with the lowest level on day 14 in the ovine uterus, which is regulated by estrogen and/or progesterone [47]. Furthermore, the expression of *AANAT* and *ASMT* genes increases in the endometrium at early stage of pregnancy in sows [48]. Our data revealed that AANAT and ASMT expression was upregulated in the endometrium at G13, G16, and G25, but was significantly declined at G70. Therefore, it is suggested the upregulation of AANAT and ASMT during pregnancy and the downregulation of these rate-limiting enzymes in the endometria at G70 may be related to the modulation of the endometrium function.

This study has several limitations. Further studies are needed to measure melatonin concentration, AANAT/ASMT activity, immune markers, oxidative stress markers, thyroid function, intestinal barrier markers, or implantation-related endpoints. In addition, location of ASMT protein in these extrapineal tissues needs to be analyzed.

## 5. Conclusions

This study demonstrates, for the first time in sheep, that pregnancy regulates the expression of AANAT and ASMT in maternal tissues, including the thymus, spleen, liver, lymph node, thyroid, duodenum, and endometrium, in a pregnancy-stage- and tissue-specific manner. Specifically, AANAT was upregulated in the liver, while ASMT showed increased expression in the thymus and duodenum. Conversely, AANAT was downregulated in the lymph nodes and duodenum. Moreover, the protein expression levels of AANAT and ASMT in these extrapineal organs exhibited distinct tissue-specific patterns. Collectively, these findings suggest that the stage- and tissue-dependent modulation of AANAT and ASMT in maternal organs may contribute to the functional adaptation of these extrapineal tissues during pregnancy, although the physiological significance of these changes warrants further investigation.

## Figures and Tables

**Table 1:** Primers used for RT-qPCR.

Gene	Primer	Sequence	Size (bp)	Accession Numbers
*AANAT*	Forward	CGTGTTTGAGATTGAGCGAGAGG	116	NM_001009461.1
	Reverse	CGAACCAGCCCAGGGACAG		
*ASMT*	Forward	GTCTCCTGAAGGAATACGCCAAC	118	NM_001306120.1
	Reverse	ACTGCCGCTGAGTCCAAGG		
*GAPDH*	Forward	GGGTCATCATCTCTGCACCT	176	NM_001190390.1
	Reverse	GGTCATAAGTCCCTCCACGA		

**Table 2:** Antibodies used for Western blot.

Description	Catalog No.	Source
Rabbit polyclonal antibody to AANAT	A11850	ABclonal Biotechnology Co., Ltd., Wuhan, Hubei, China
Rabbit polyclonal antibody to ASMT	A6529	ABclonal Biotechnology Co., Ltd., Wuhan, Hubei, China
Mouse monoclonal antibody to GAPDH	sc-47724	Santa Cruz Biotechnology, Santa Cruz, CA, USA

## Data Availability

The original contributions presented in this study are included in the article/Supplementary Material. Further inquiries can be directed to the corresponding author.

## Supplementary Materials

The following supporting information can be downloaded at https://www.mdpi.com/article/10.3390/biom16071047/s1, Figure S1: Original Western blot for 1–7; Figure S2: Melt curve and amplification.

## Abbreviations

The following abbreviations are used in this manuscript:

AANAT	Aralkylamine N-acetyltransferase
ASMT	Acetylserotonin O-methyltransferase
GAPDH	Glyceraldehyde-3-phosphate dehydrogenase
IFNT	Interferon-tau
MT1	Melatonin membrane receptor 1
