# Supplementary material for: Expression of Rate-Limiting Enzymes of Melatonin Synthesis in Several Extrapineal Organs During Pregnancy in Ewes
Source: Biomolecules. 2026 Jul 17;16(7):1047. doi: 10.3390/biom16071047 (PMC13406823; doi:10.3390/biom16071047)
Supplement: Supplementary file 1 [file biomolecules-16-01047-s001.zip › Figure S1 Original Western Blot for 1-7.pdf]

Figure S1: Original Western Blot for 1-7.

1 Thymus

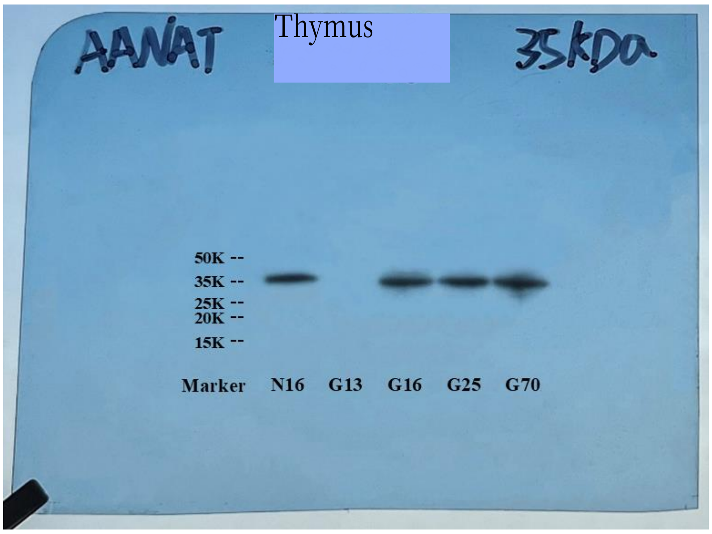

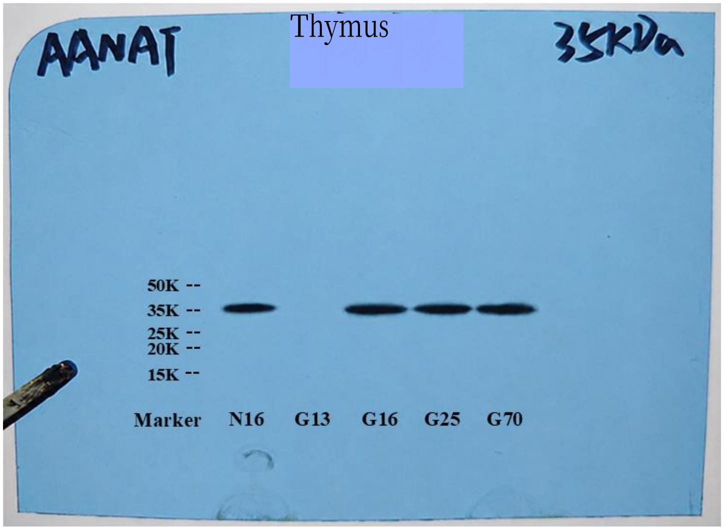

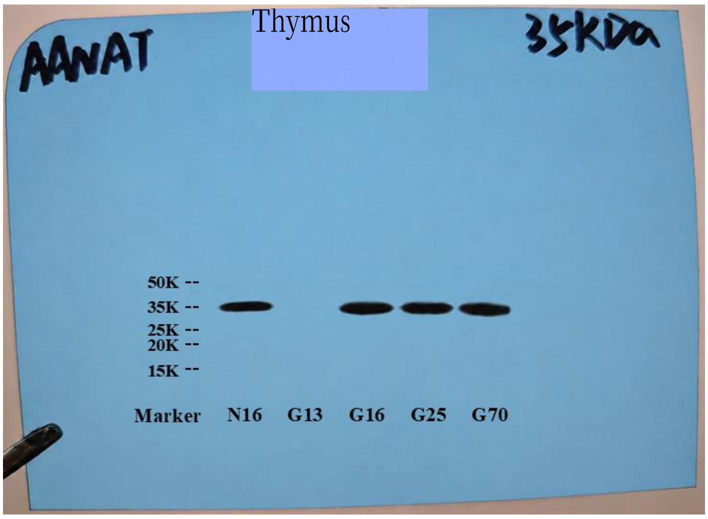

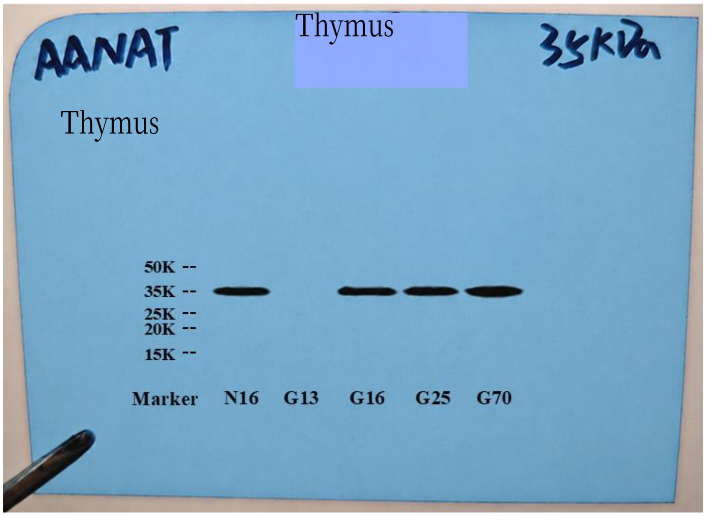

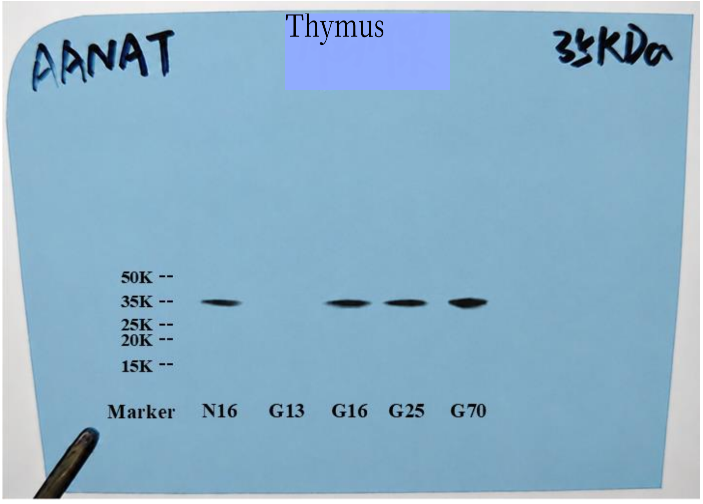

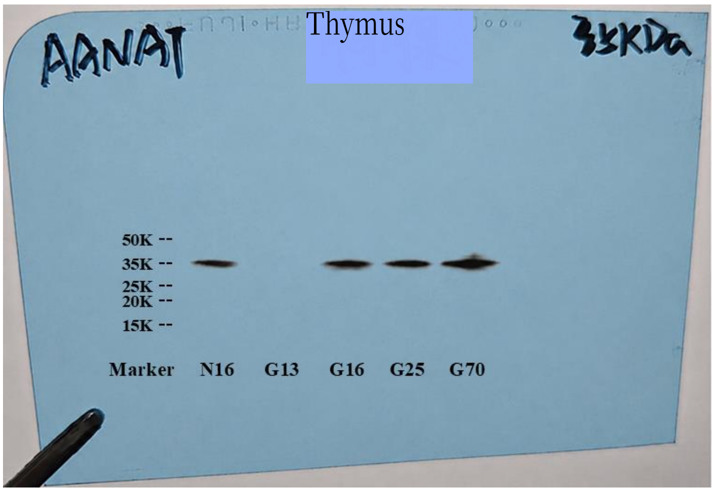

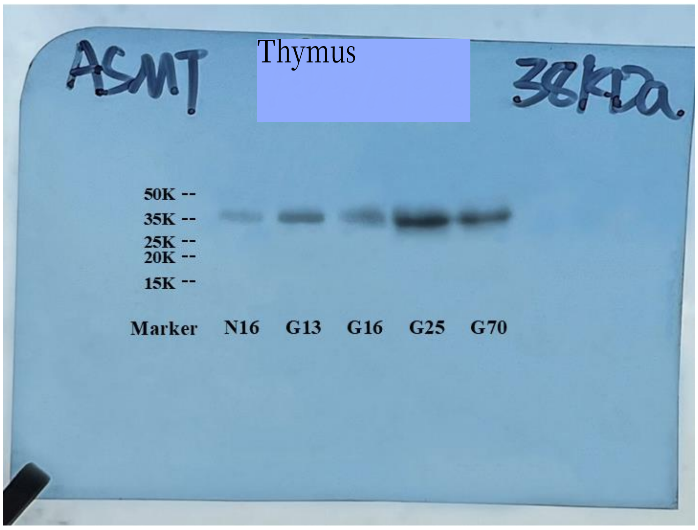
ASMT
Thymus
38KDa
50K --
35K --
25K --
20K --
15K --
Marker N16 G13 G16 G25 G70

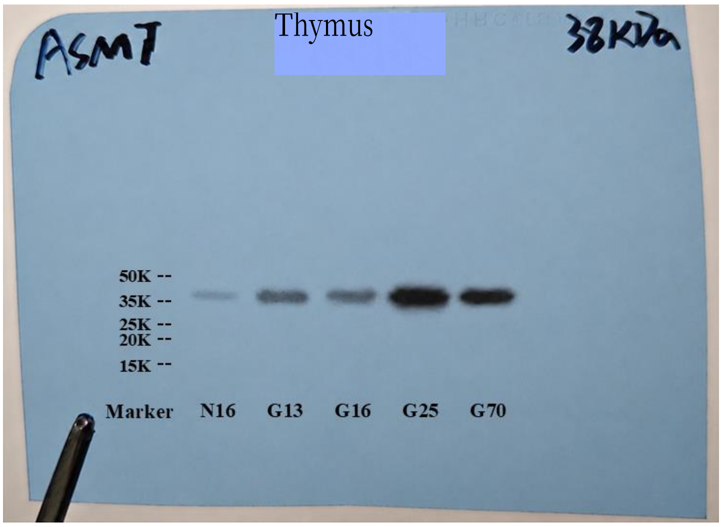
ASMT
Thymus
38KDa
50K --
35K --
25K --
20K --
15K --
Marker N16 G13 G16 G25 G70

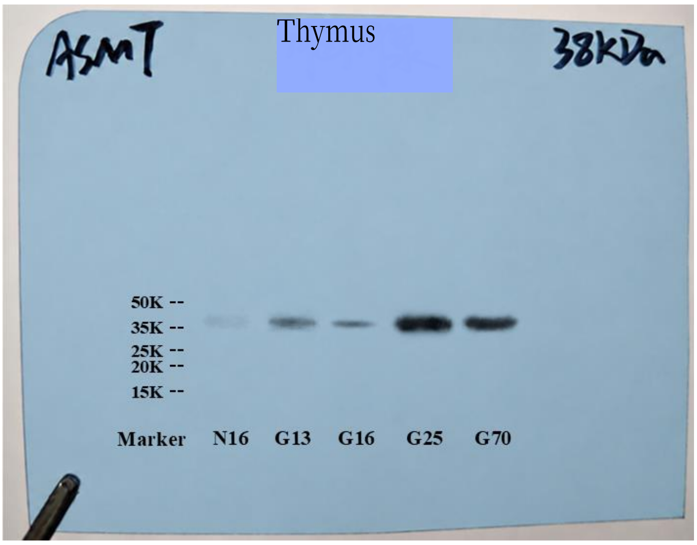
ASMT
Thymus
38KDa
50K --
35K --
25K --
20K --
15K --
Marker N16 G13 G16 G25 G70

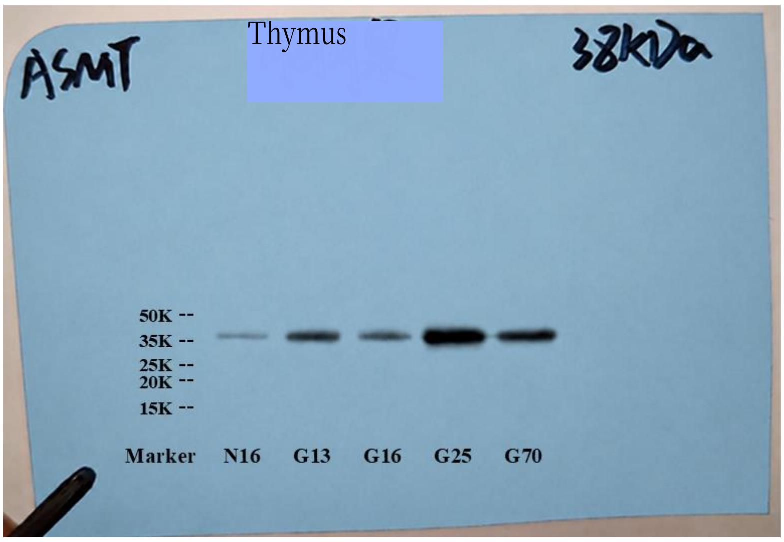
ASMT
Thymus
38KDa
50K --
35K --
25K --
20K --
15K --
Marker N16 G13 G16 G25 G70

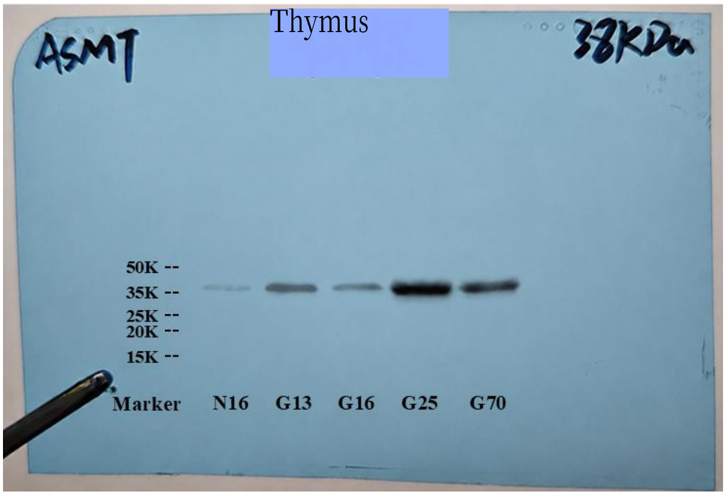
ASMT
Thymus
38KDa
50K --
35K --
25K --
20K --
15K --
Marker N16 G13 G16 G25 G70

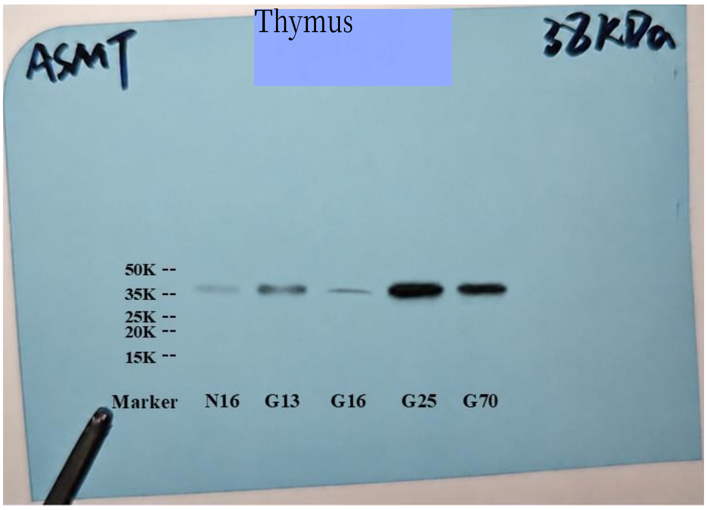
ASMT
Thymus
38KDa
50K --
35K --
25K --
20K --
15K --
Marker N16 G13 G16 G25 G70

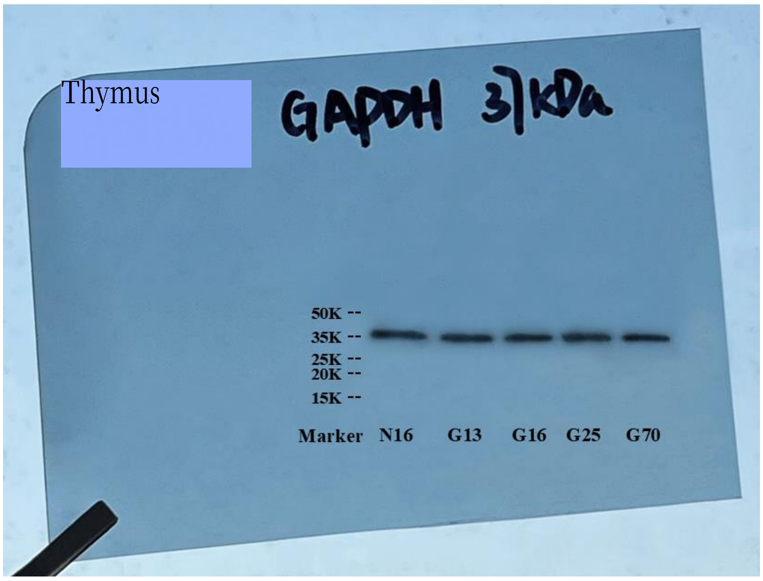
Thymus
GAPDH 37kDa
50K
35K
25K
20K
15K
Marker
N16
G13
G16
G25
G70

2 Lymph nodes

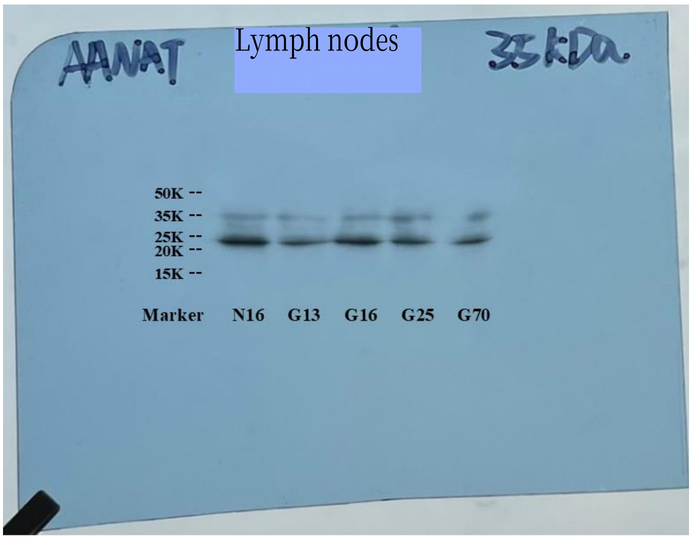

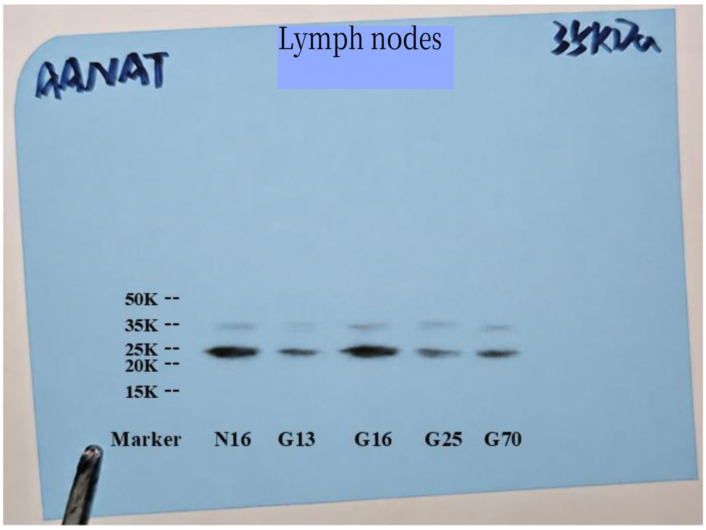

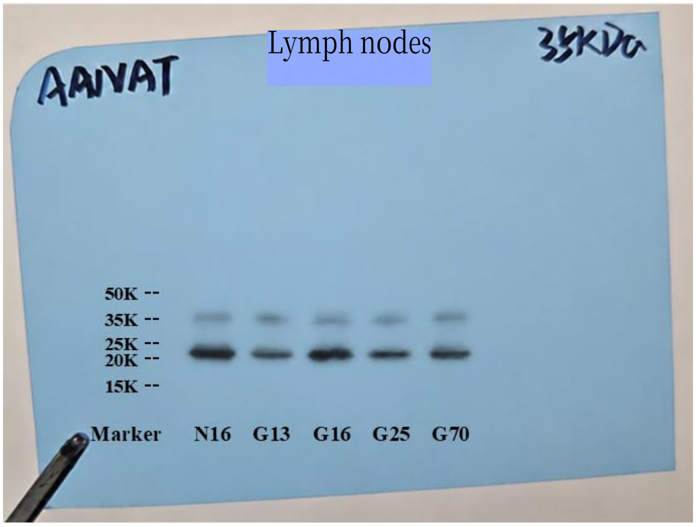

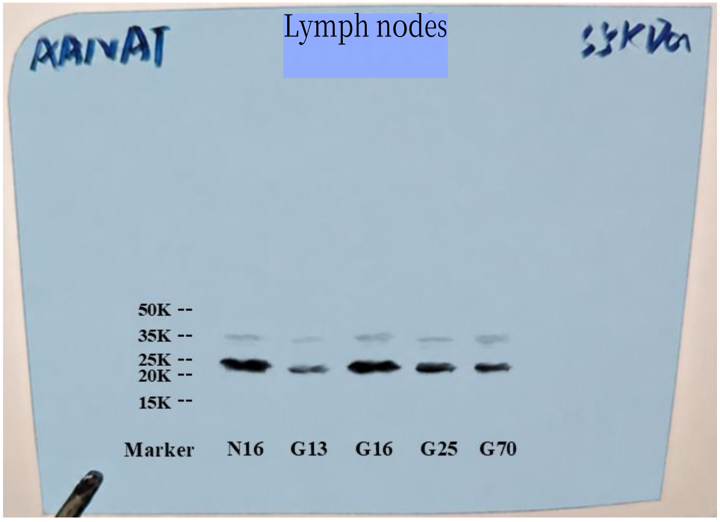

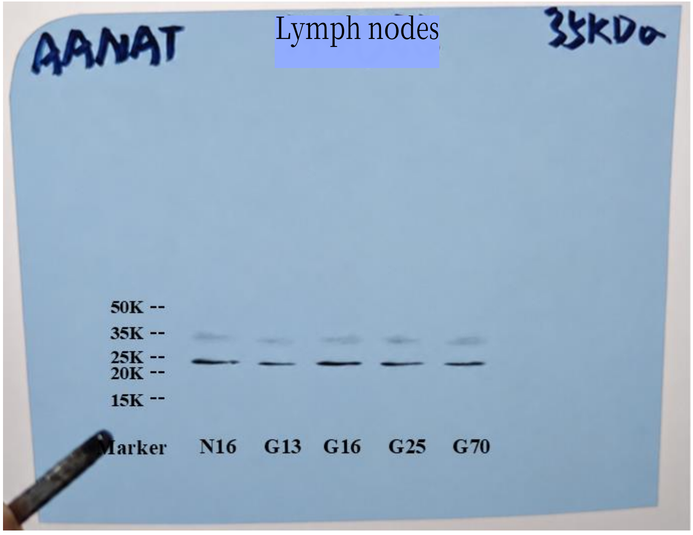

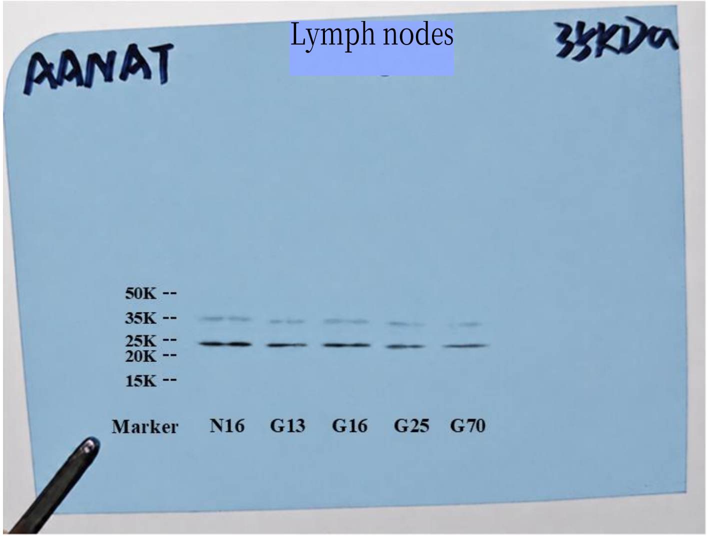

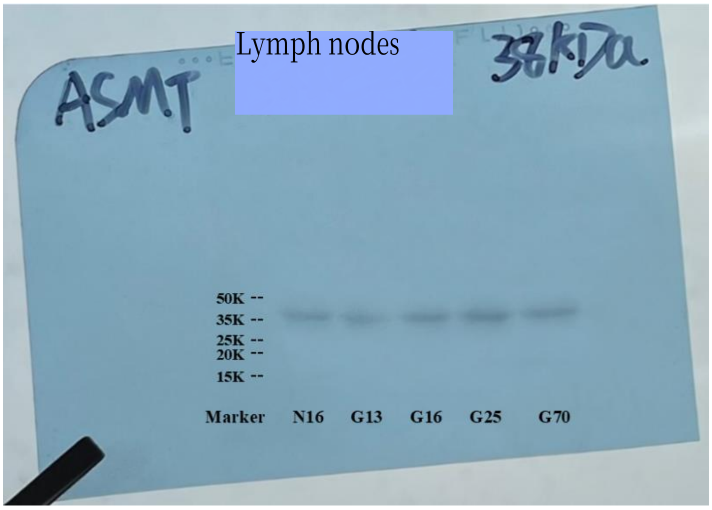
ASMT
Lymph nodes
38kDa
50K
35K
25K
20K
15K
Marker N16 G13 G16 G25 G70

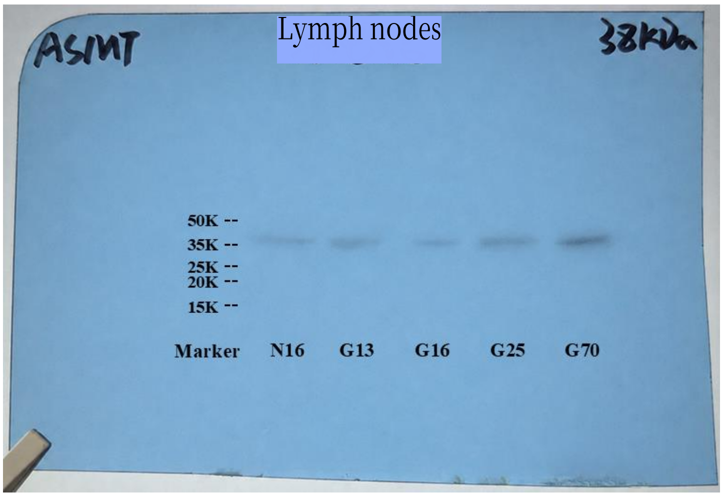
ASMT
Lymph nodes
38kDa
50K
35K
25K
20K
15K
Marker N16 G13 G16 G25 G70

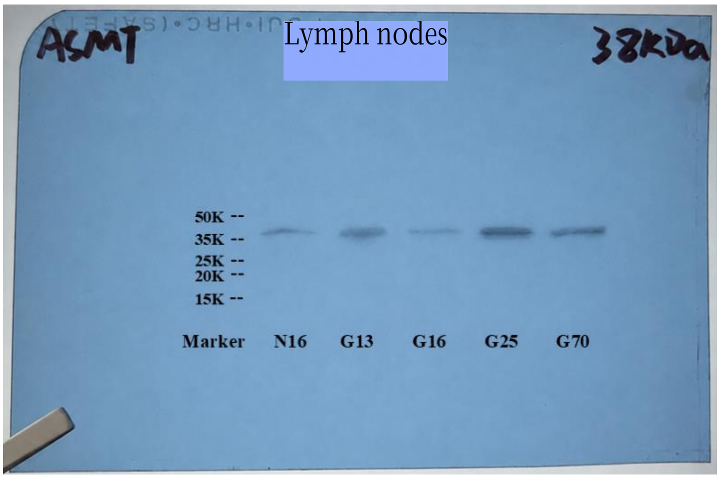
ASMT
Lymph nodes
38kDa
50K
35K
25K
20K
15K
Marker N16 G13 G16 G25 G70

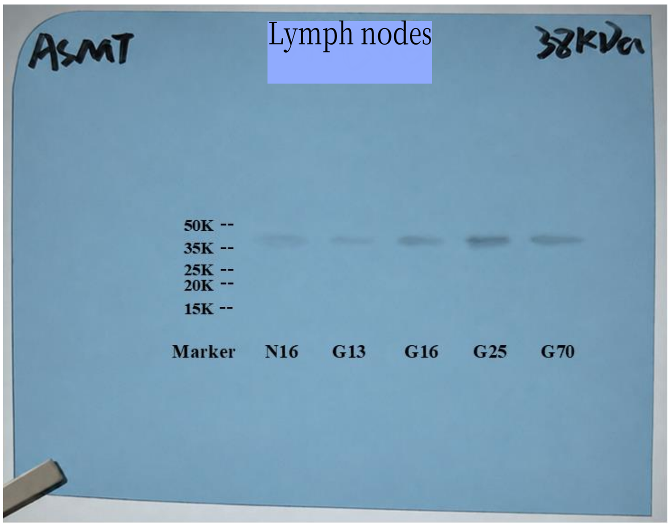
ASMT
Lymph nodes
38kDa
50K
35K
25K
20K
15K
Marker N16 G13 G16 G25 G70

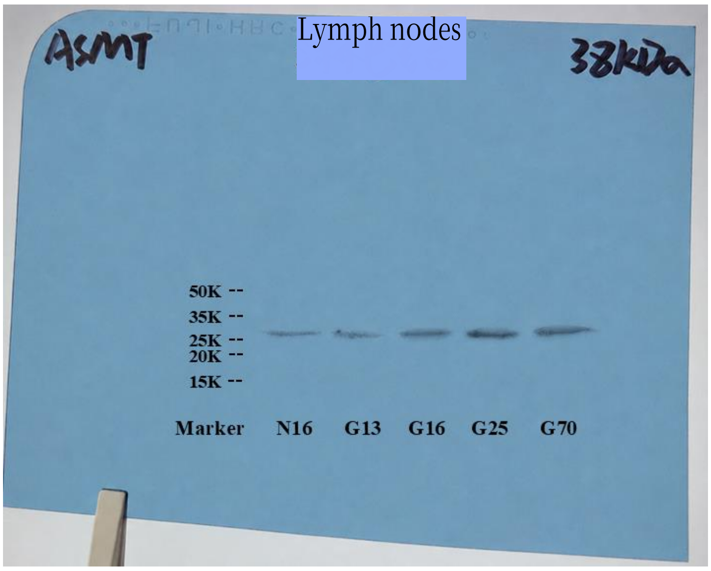
ASMT
Lymph nodes
38kDa
50K
35K
25K
20K
15K
Marker N16 G13 G16 G25 G70

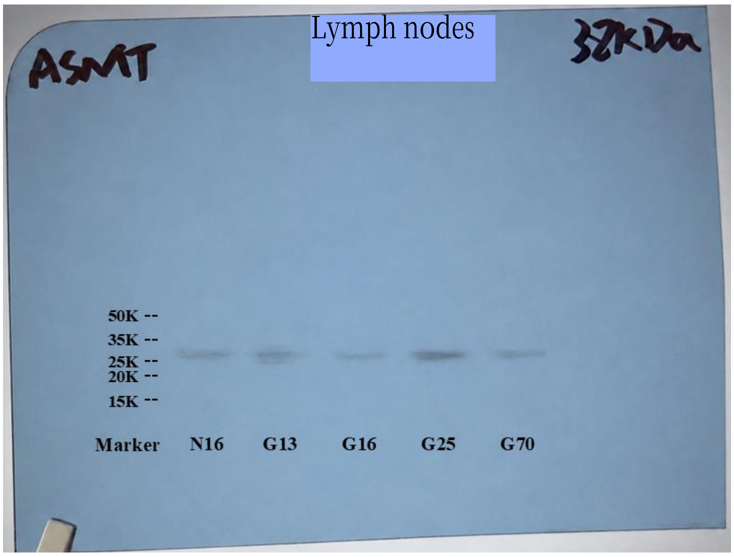
ASMT
Lymph nodes
50K
35K
25K
20K
15K
Marker N16 G13 G16 G25 G70

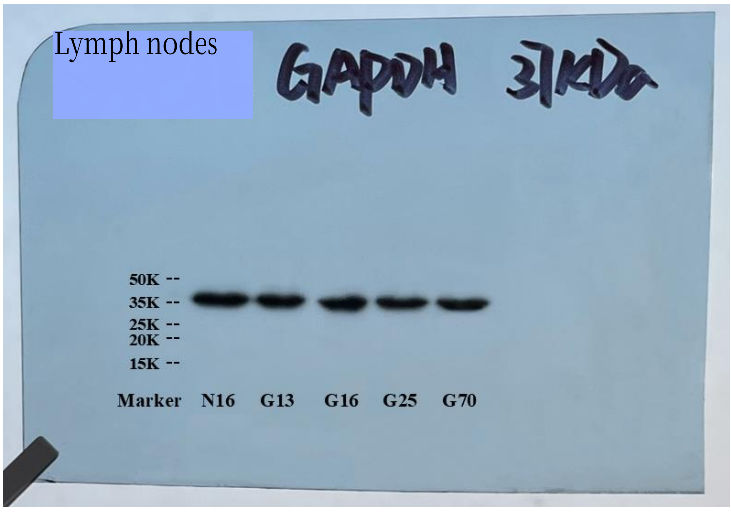
Lymph nodes
GAPDH 37KDa
50K
35K
25K
20K
15K
Marker
N16
G13
G16
G25
G70

3 Spleen

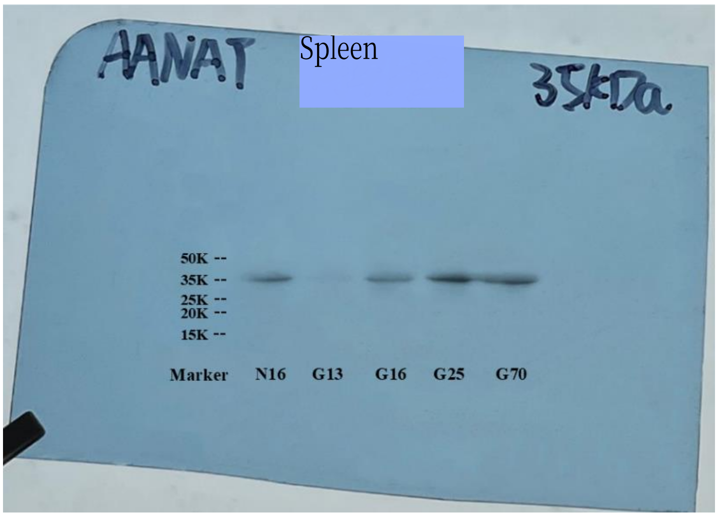
AANAT
Spleen
35kDa
50K --
35K --
25K --
20K --
15K --
Marker N16 G13 G16 G25 G70

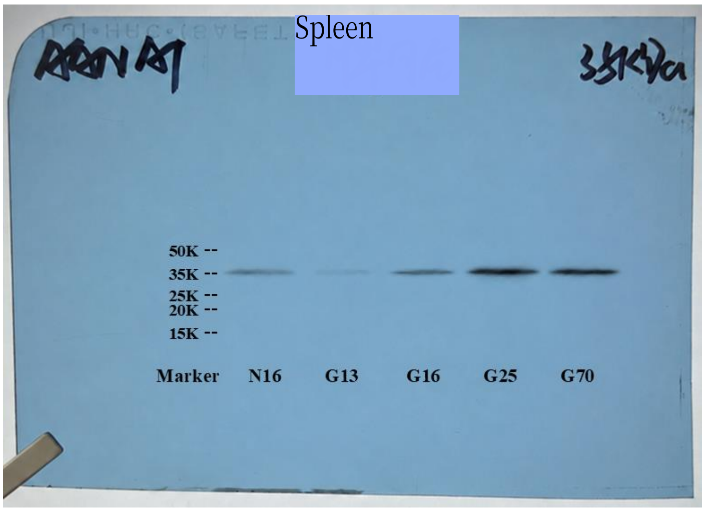
Spleen
50K --
35K --
25K --
20K --
15K --
Marker N16 G13 G16 G25 G70

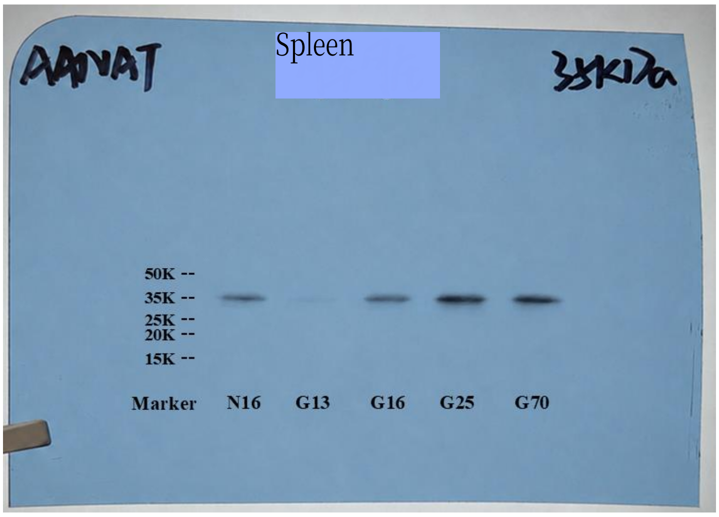
Spleen
50K --
35K --
25K --
20K --
15K --
Marker N16 G13 G16 G25 G70

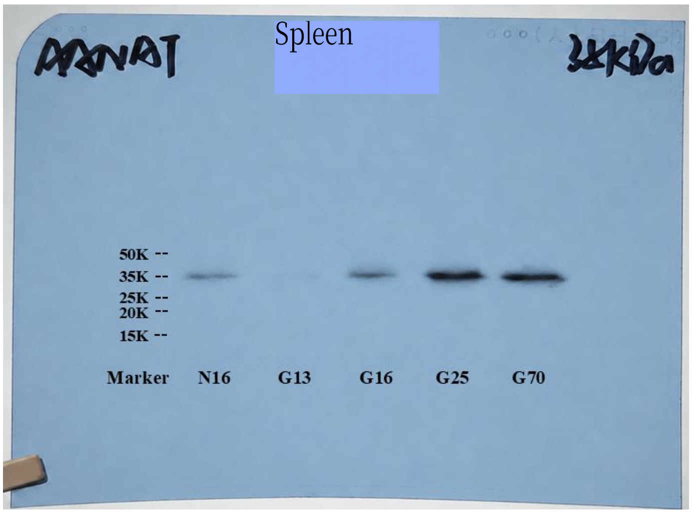
Spleen
50K --
35K --
25K --
20K --
15K --
Marker N16 G13 G16 G25 G70

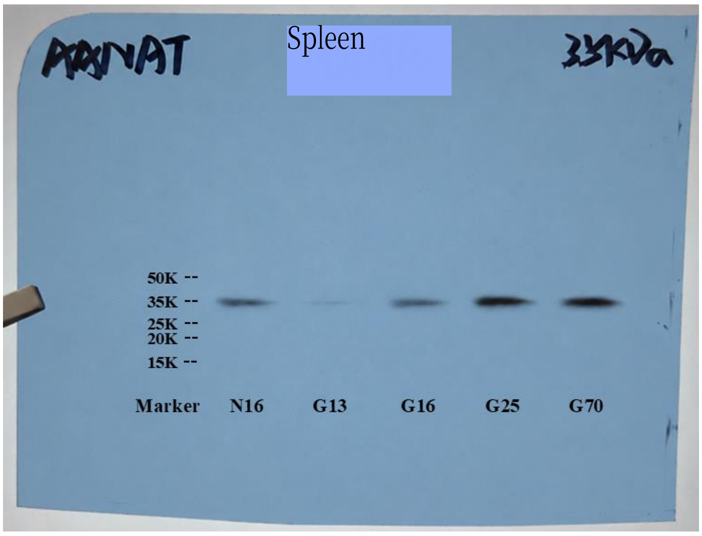
Spleen
50K --
35K --
25K --
20K --
15K --
Marker N16 G13 G16 G25 G70

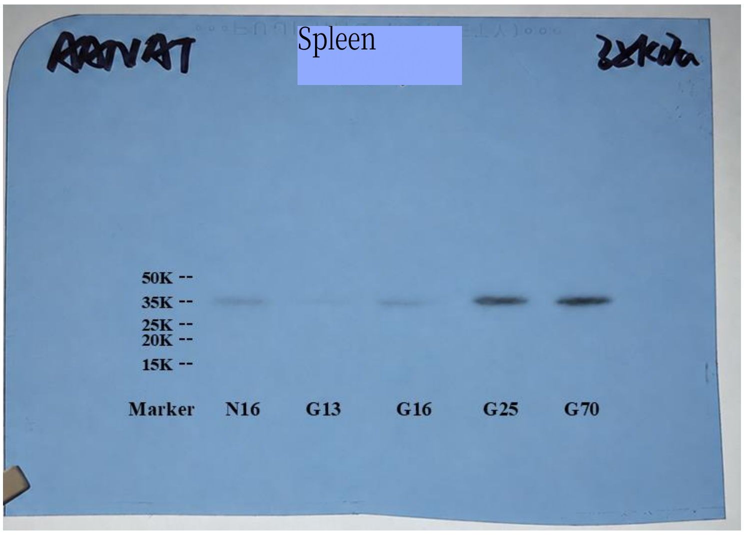
Spleen
50K --
35K --
25K --
20K --
15K --
Marker N16 G13 G16 G25 G70

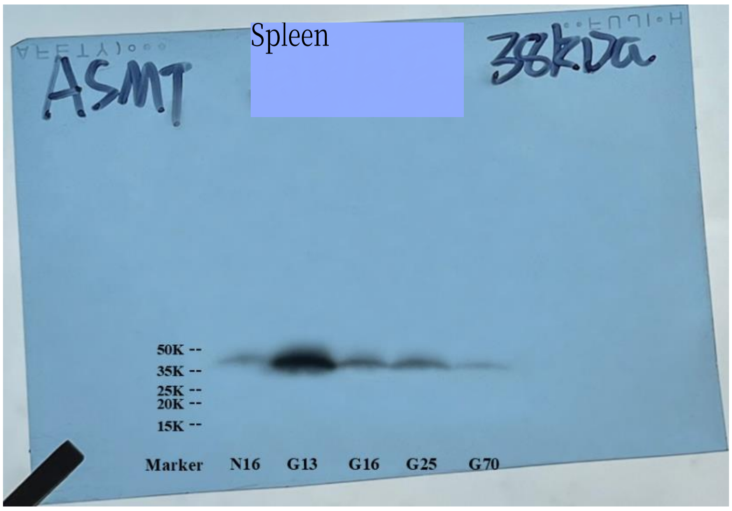
ASMT
Spleen
38kDa
50K
35K
25K
20K
15K
Marker
N16
G13
G16
G25
G70

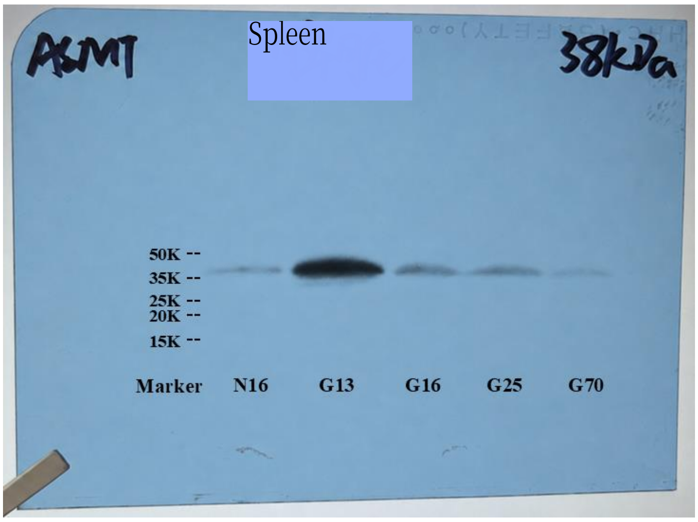
ASMT
Spleen
38kDa
50K
35K
25K
20K
15K
Marker
N16
G13
G16
G25
G70

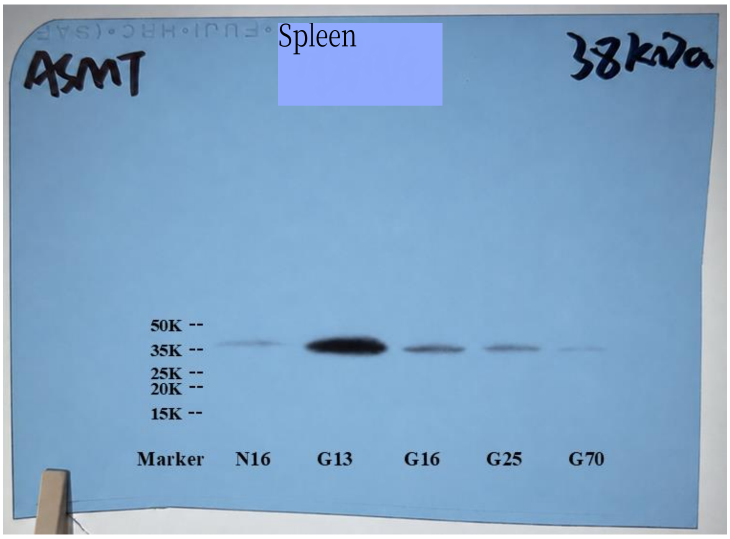
ASMT
Spleen
38kDa
50K
35K
25K
20K
15K
Marker
N16
G13
G16
G25
G70

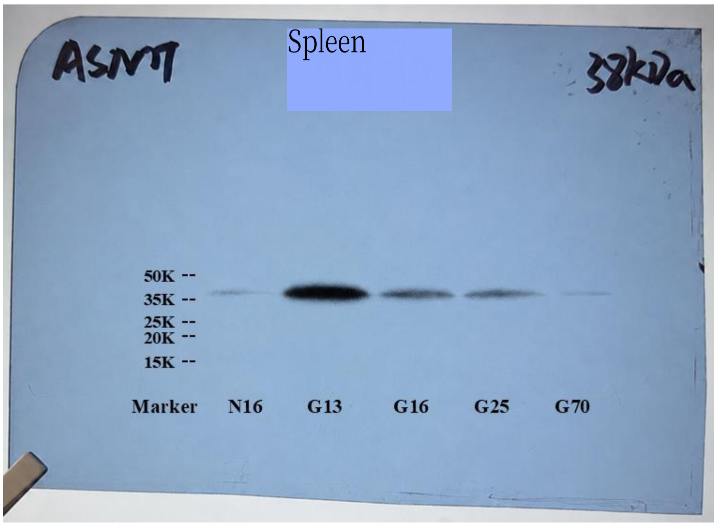
ASMT
Spleen
38kDa
50K
35K
25K
20K
15K
Marker
N16
G13
G16
G25
G70

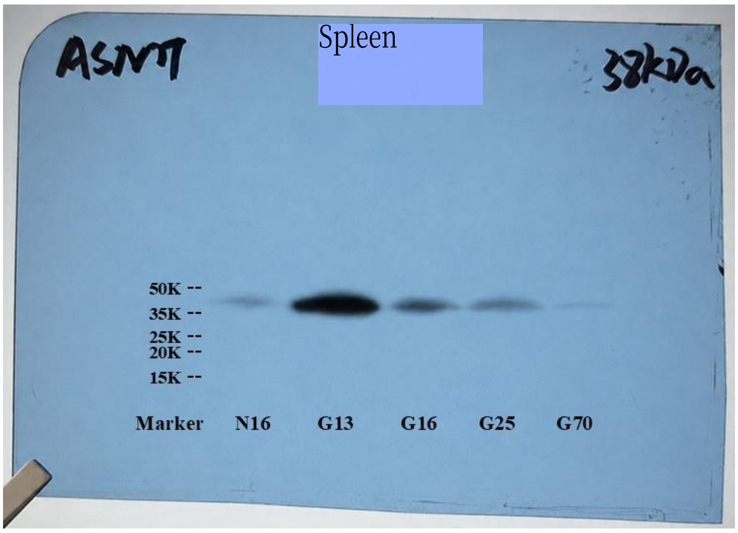
ASMT
Spleen
38kDa
50K
35K
25K
20K
15K
Marker
N16
G13
G16
G25
G70

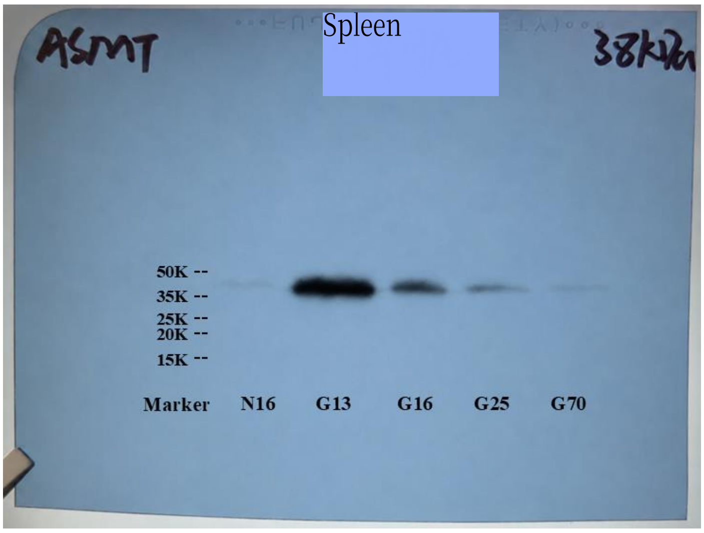
ASMT
Spleen
38kDa
50K
35K
25K
20K
15K
Marker
N16
G13
G16
G25
G70

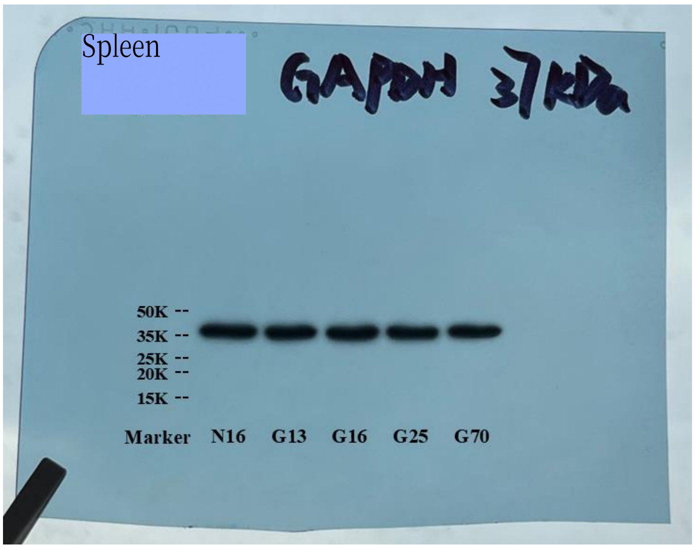
Spleen
GAPDH 37kDa
50K
35K
25K
20K
15K
Marker
N16
G13
G16
G25
G70

4 Liver

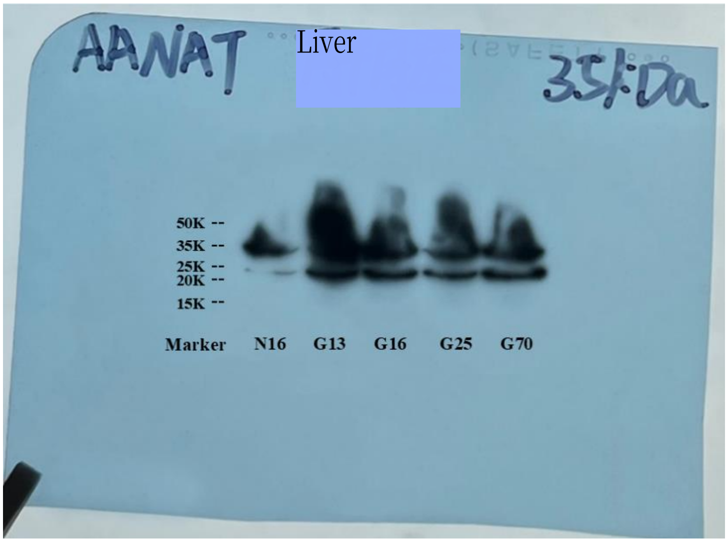
AANAT
Liver
35KDa
50K
35K
25K
20K
15K
Marker N16 G13 G16 G25 G70

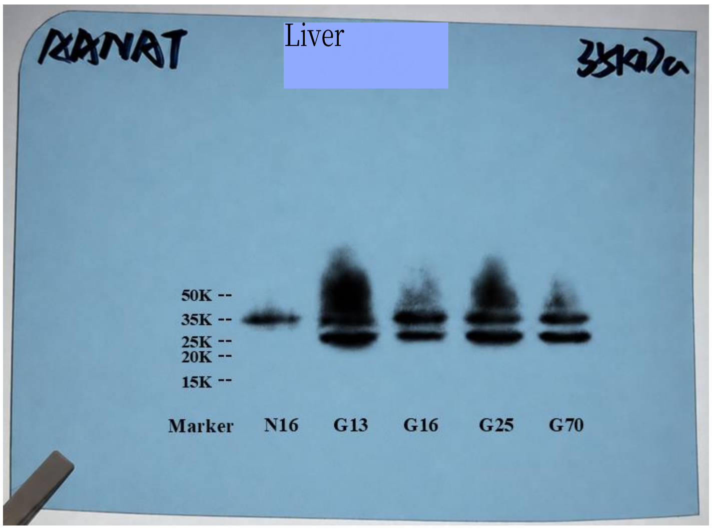
AANAT
Liver
35KDa
50K
35K
25K
20K
15K
Marker N16 G13 G16 G25 G70

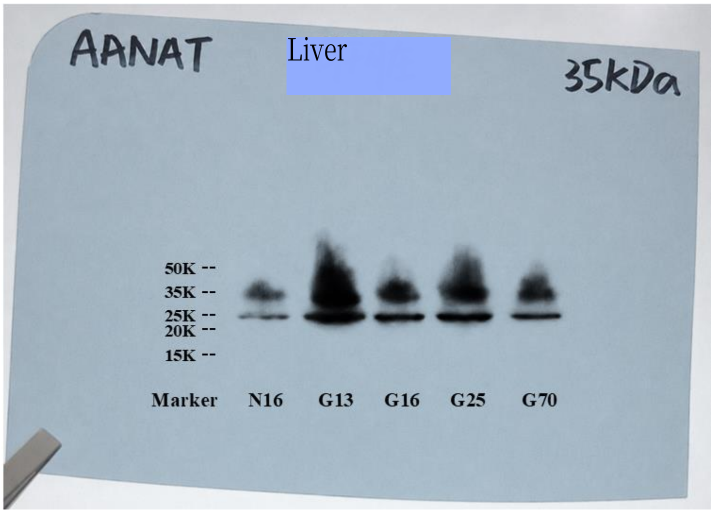
AANAT
Liver
35KDa
50K
35K
25K
20K
15K
Marker N16 G13 G16 G25 G70

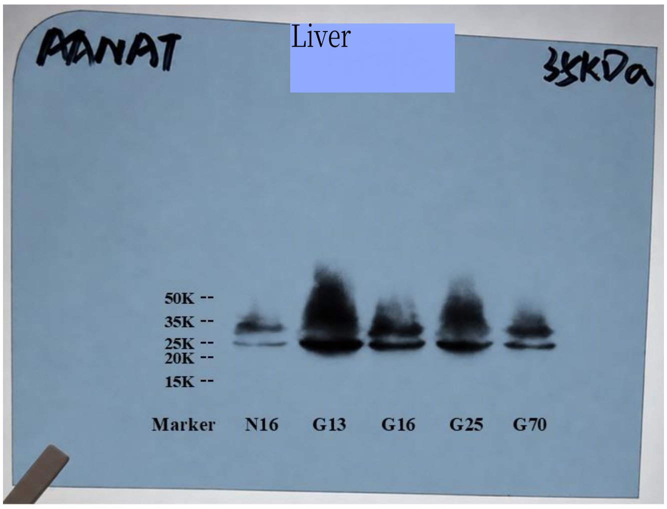
AANAT
Liver
35KDa
50K
35K
25K
20K
15K
Marker N16 G13 G16 G25 G70

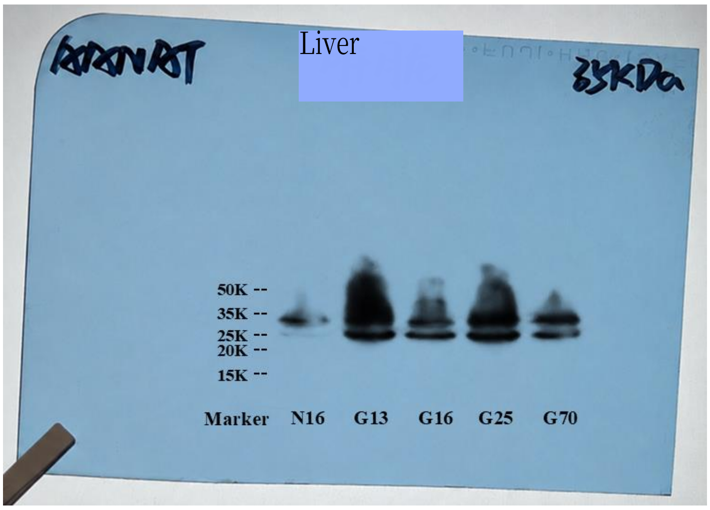
AANAT
Liver
35KDa
50K
35K
25K
20K
15K
Marker N16 G13 G16 G25 G70

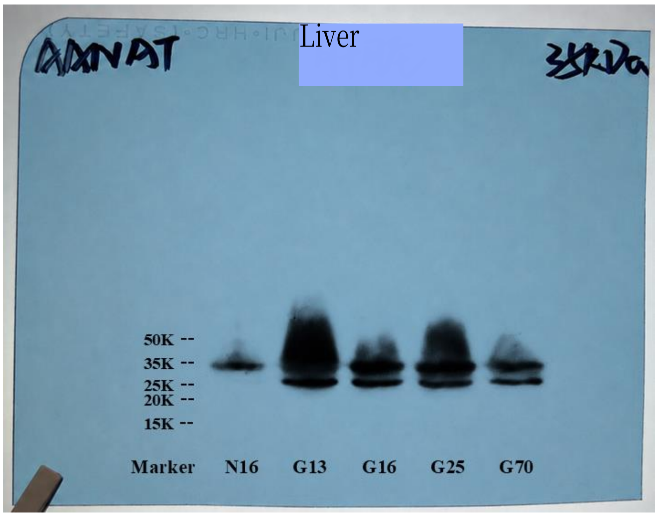
AANAT
Liver
35KDa
50K
35K
25K
20K
15K
Marker N16 G13 G16 G25 G70

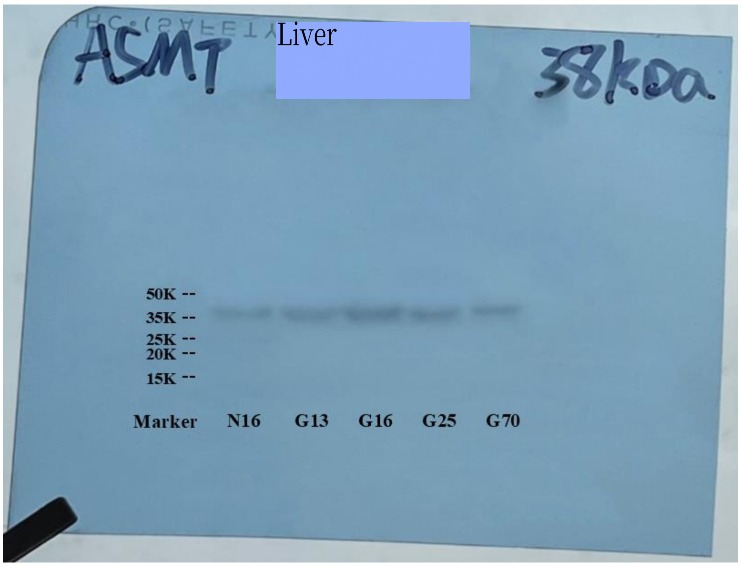
ASMT
Liver
38kDa
50K --
35K --
25K --
20K --
15K --
Marker N16 G13 G16 G25 G70

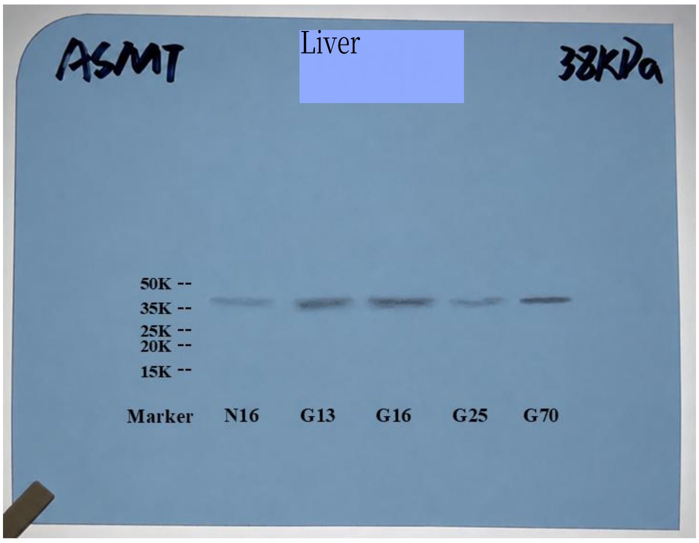
ASMT
Liver
38KDa
50K --
35K --
25K --
20K --
15K --
Marker N16 G13 G16 G25 G70

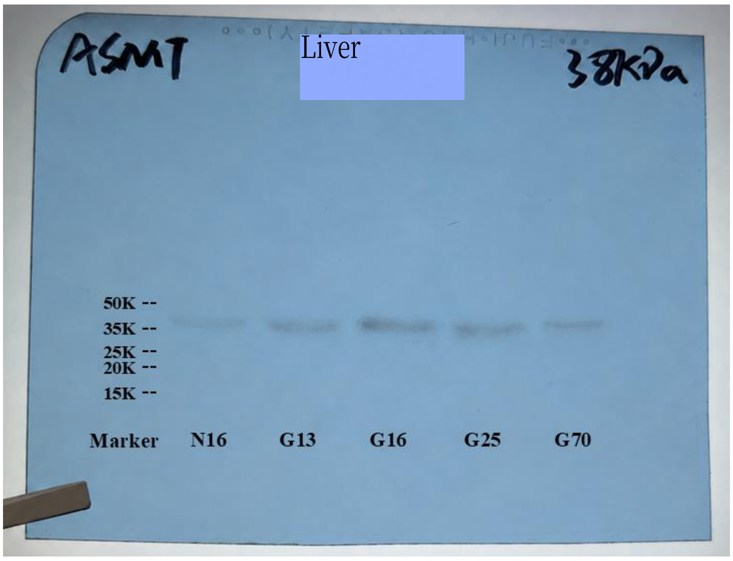
ASMT
Liver
38KDa
50K --
35K --
25K --
20K --
15K --
Marker N16 G13 G16 G25 G70

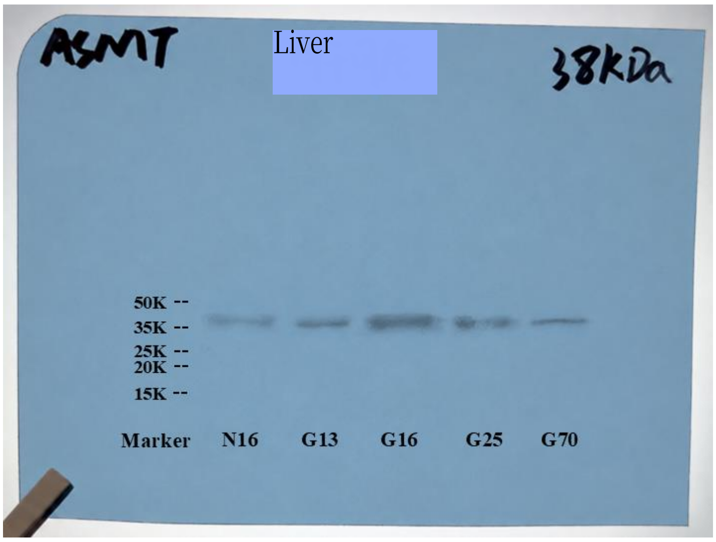
ASMT
Liver
38KDa
50K --
35K --
25K --
20K --
15K --
Marker N16 G13 G16 G25 G70

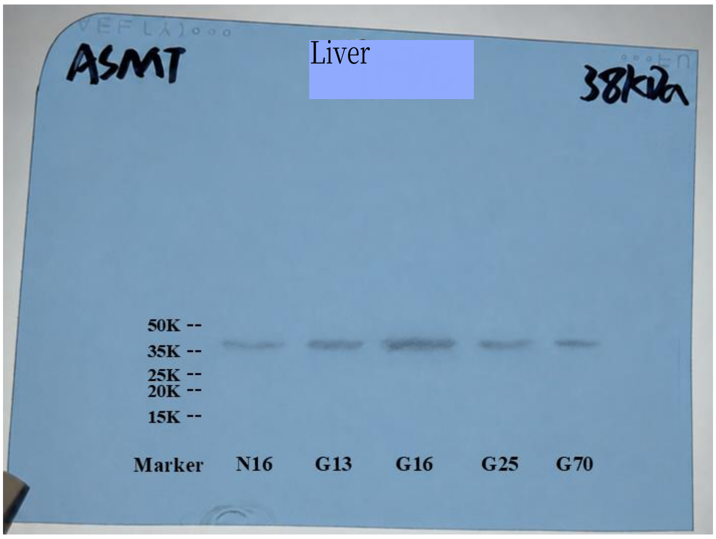
ASMT
Liver
38KDa
50K --
35K --
25K --
20K --
15K --
Marker N16 G13 G16 G25 G70

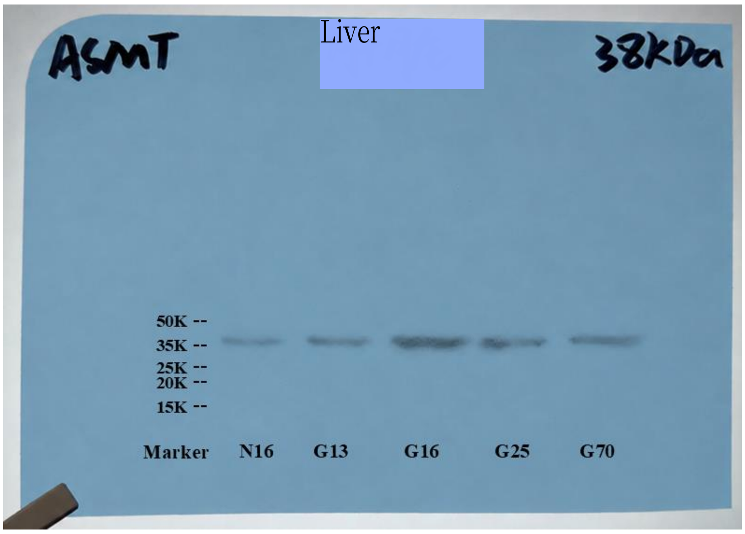
ASMT
Liver
38KDa
50K --
35K --
25K --
20K --
15K --
Marker N16 G13 G16 G25 G70

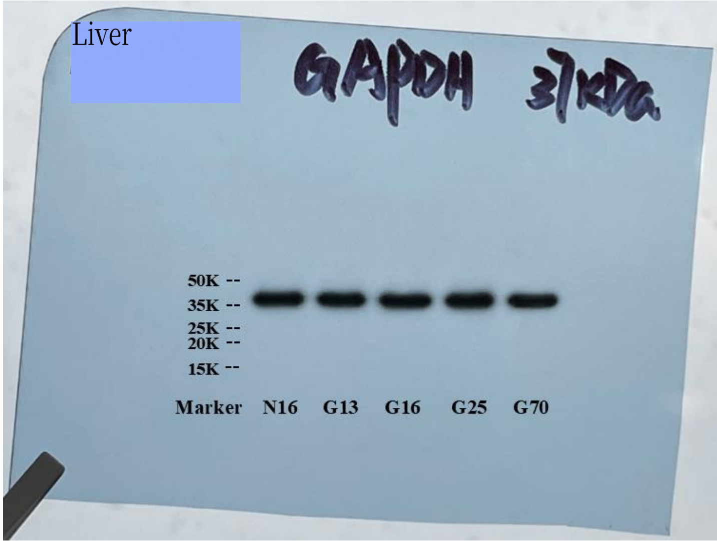
Liver
GAPDH 37kDa
50K
35K
25K
20K
15K
Marker N16 G13 G16 G25 G70

5 Thyroid

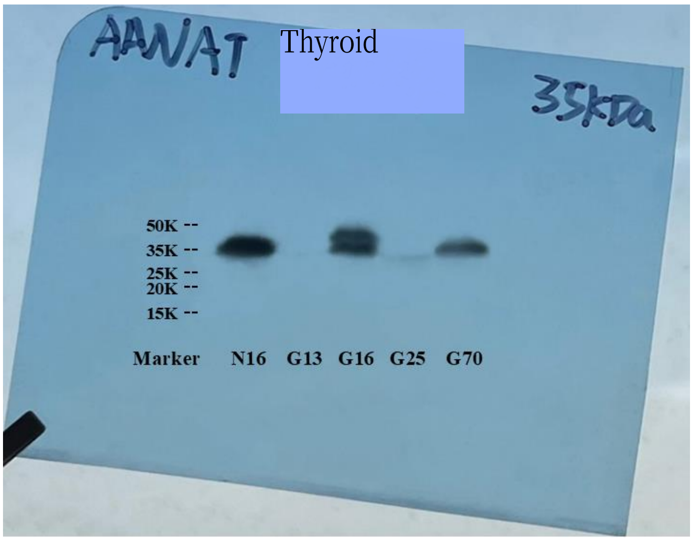
AANAT
Thyroid
35kDa
50K
35K
25K
20K
15K
Marker N16 G13 G16 G25 G70

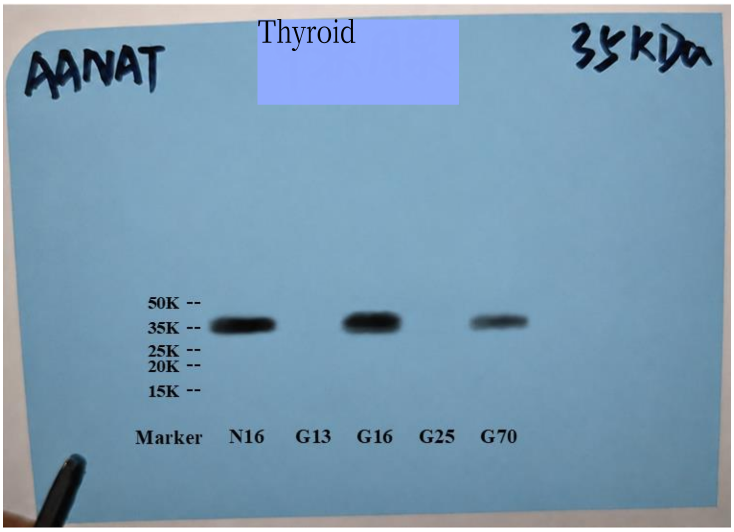
AANAT
Thyroid
35kDa
50K
35K
25K
20K
15K
Marker N16 G13 G16 G25 G70

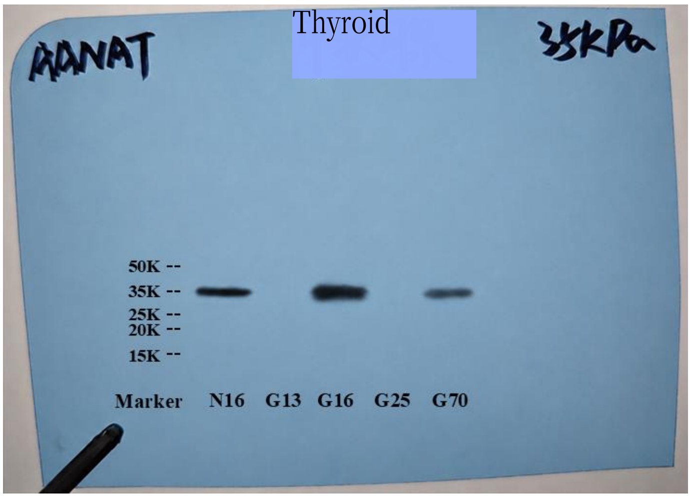
AANAT
Thyroid
35kDa
50K
35K
25K
20K
15K
Marker N16 G13 G16 G25 G70

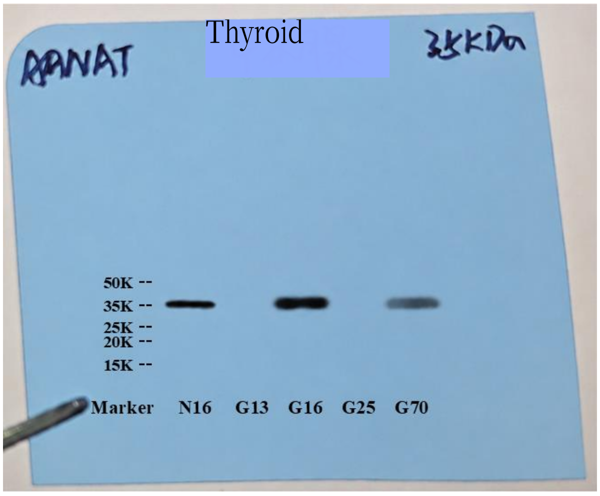
AANAT
Thyroid
35kDa
50K
35K
25K
20K
15K
Marker N16 G13 G16 G25 G70

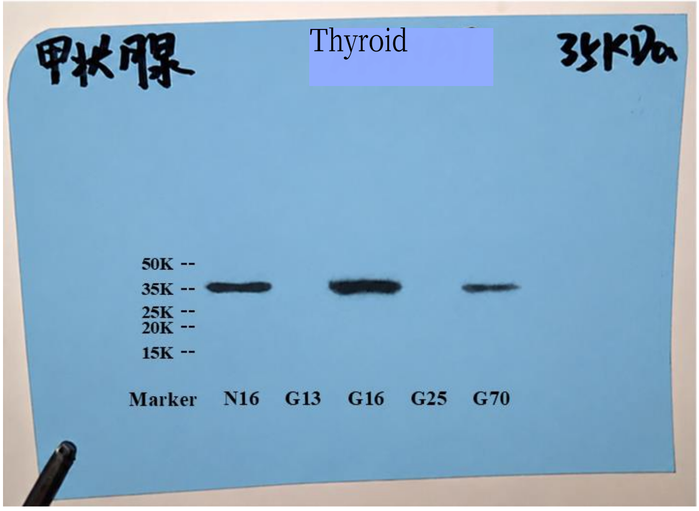
甲状腺
Thyroid
35kDa
50K
35K
25K
20K
15K
Marker N16 G13 G16 G25 G70

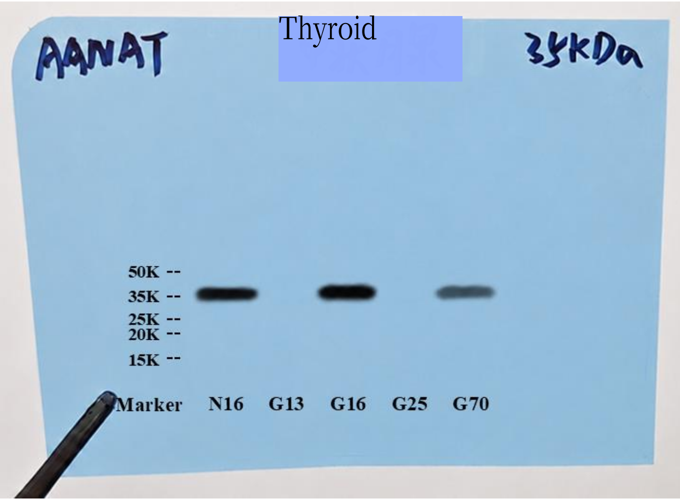
AANAT
Thyroid
35kDa
50K
35K
25K
20K
15K
Marker N16 G13 G16 G25 G70

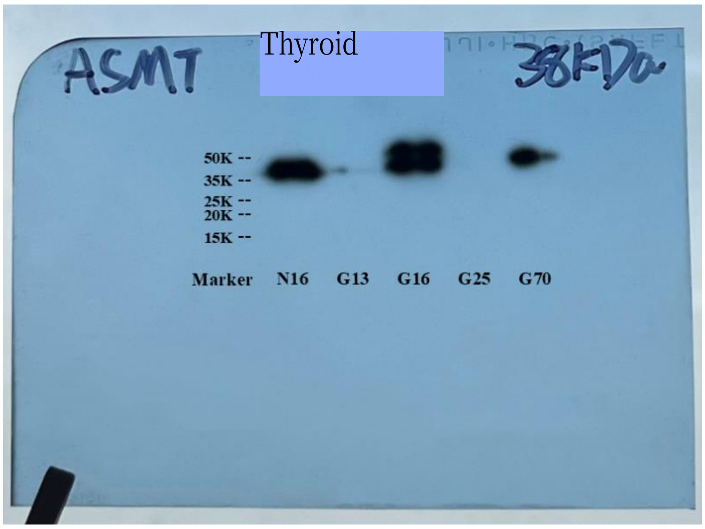
ASMT
Thyroid
38KDa
50K --
35K --
25K --
20K --
15K --
Marker N16 G13 G16 G25 G70
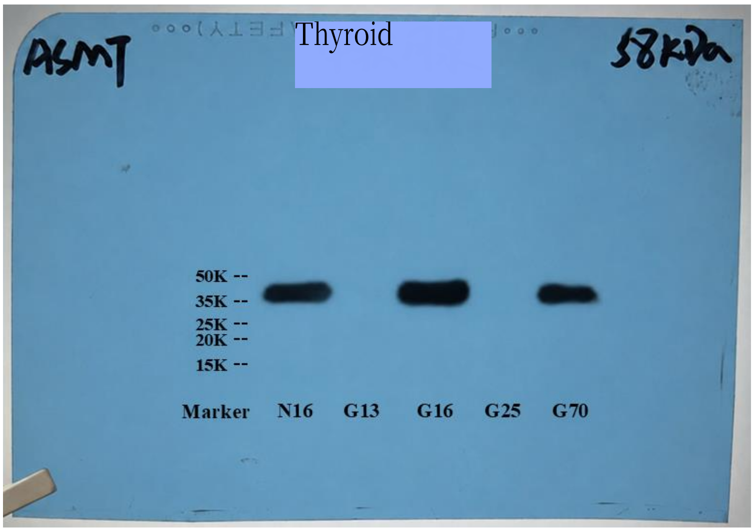
ASMT
Thyroid
38kDa
50K --
35K --
25K --
20K --
15K --
Marker N16 G13 G16 G25 G70
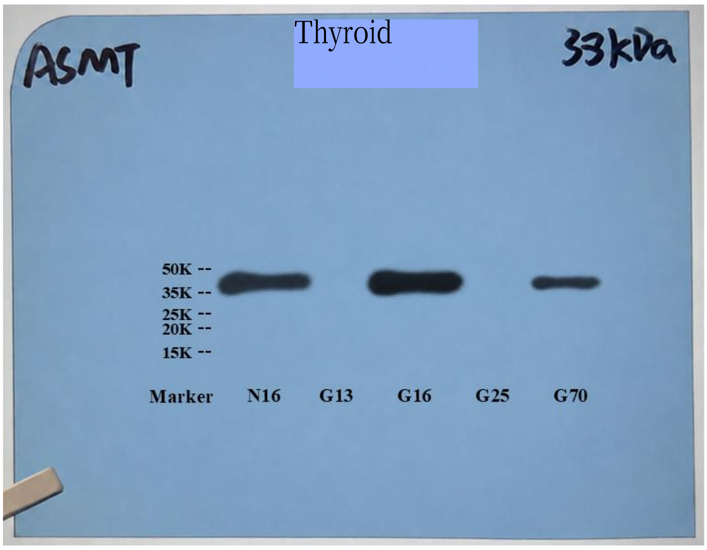
ASMT
Thyroid
33kDa
50K --
35K --
25K --
20K --
15K --
Marker N16 G13 G16 G25 G70
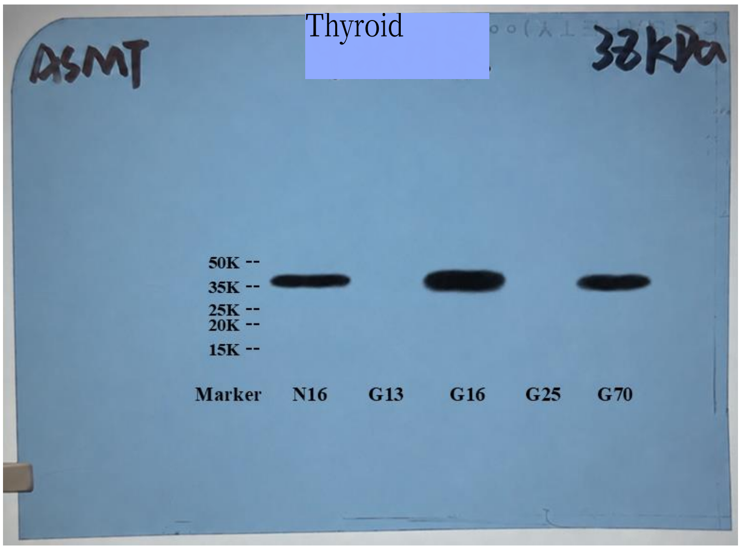
ASMT
Thyroid
38kDa
50K --
35K --
25K --
20K --
15K --
Marker N16 G13 G16 G25 G70
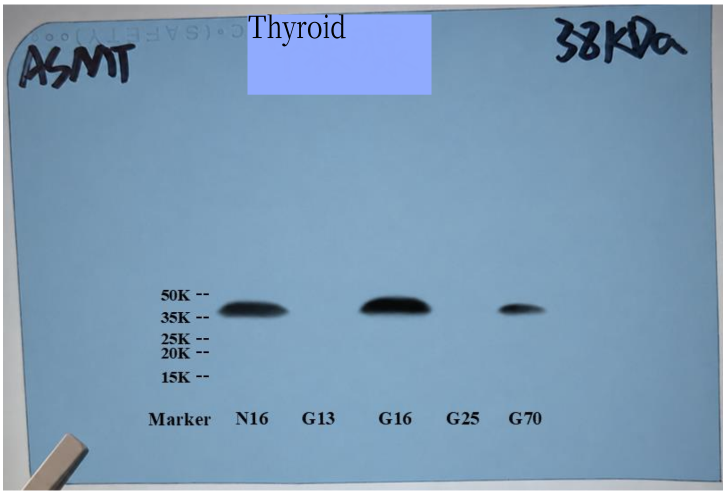
ASMT
Thyroid
38KDa
50K --
35K --
25K --
20K --
15K --
Marker N16 G13 G16 G25 G70
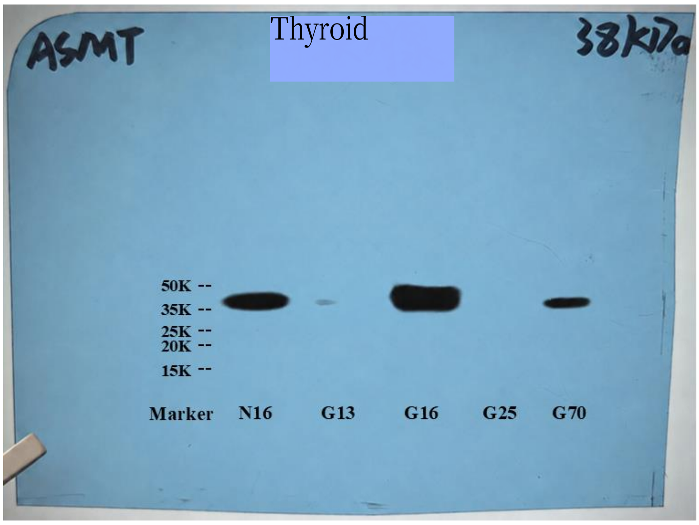
ASMT
Thyroid
38kDa
50K --
35K --
25K --
20K --
15K --
Marker N16 G13 G16 G25 G70

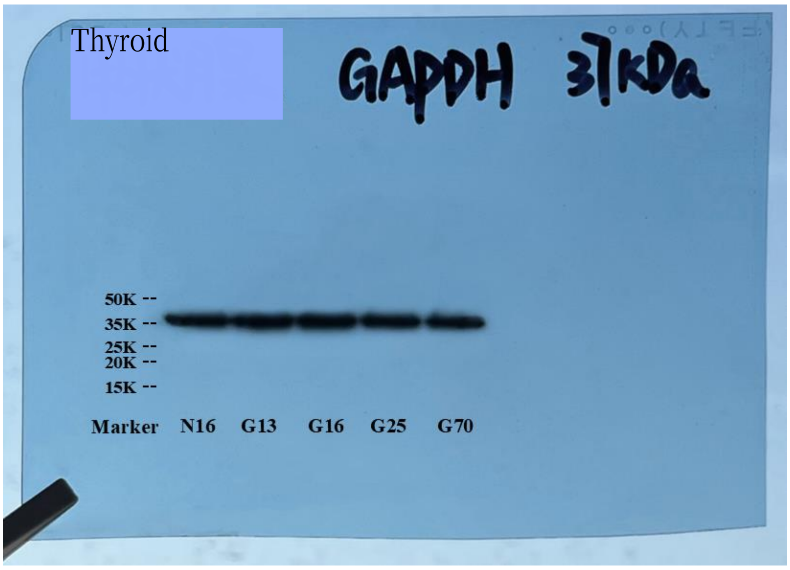
Thyroid
GAPDH 37kDa
50K
35K
25K
20K
15K
Marker
N16
G13
G16
G25
G70

6 Duodenum

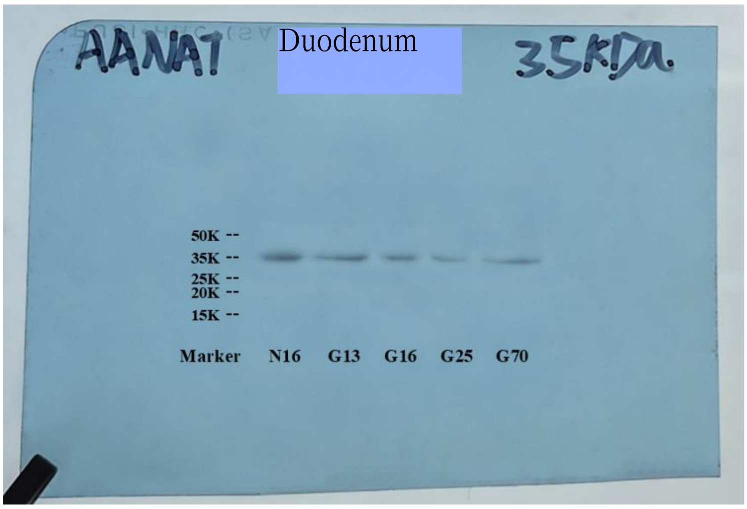
AANAT
Duodenum
35KDa.
50K --
35K --
25K --
20K --
15K --
Marker N16 G13 G16 G25 G70

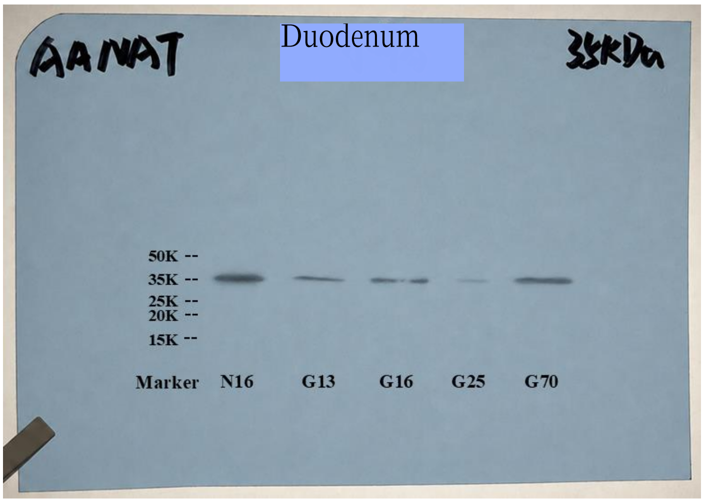
AANAT
Duodenum
35KDa
50K --
35K --
25K --
20K --
15K --
Marker N16 G13 G16 G25 G70

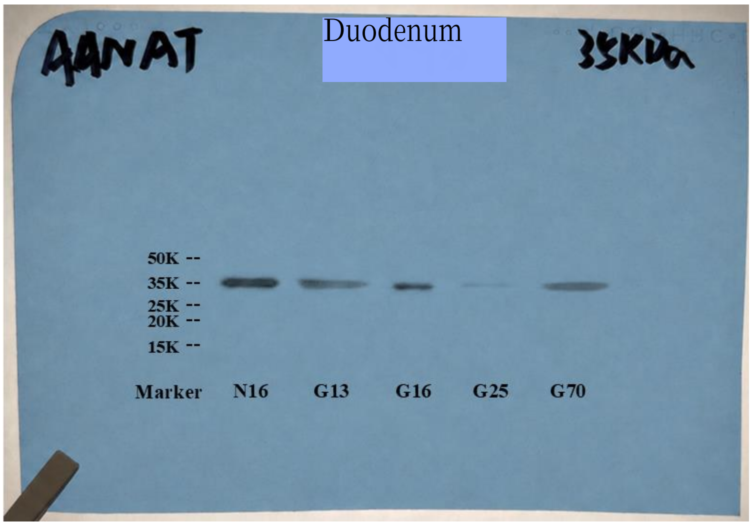
AANAT
Duodenum
35KDa
50K --
35K --
25K --
20K --
15K --
Marker N16 G13 G16 G25 G70

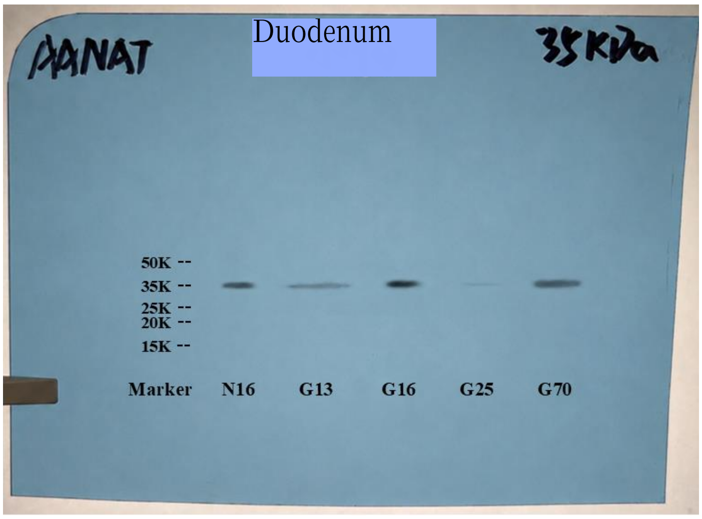
AANAT
Duodenum
35KDa
50K --
35K --
25K --
20K --
15K --
Marker N16 G13 G16 G25 G70

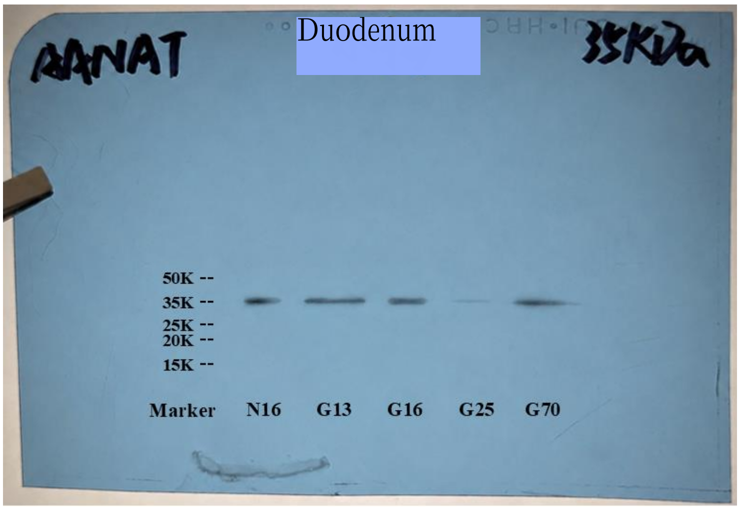
AANAT
Duodenum
35KDa
50K --
35K --
25K --
20K --
15K --
Marker N16 G13 G16 G25 G70

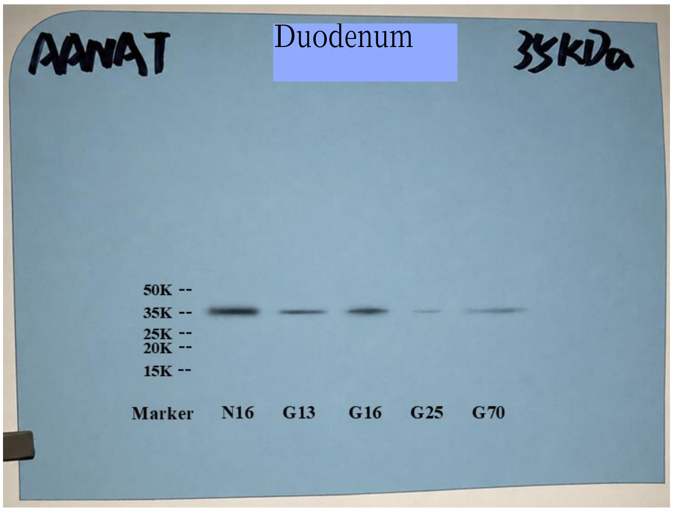
AANAT
Duodenum
35KDa
50K --
35K --
25K --
20K --
15K --
Marker N16 G13 G16 G25 G70

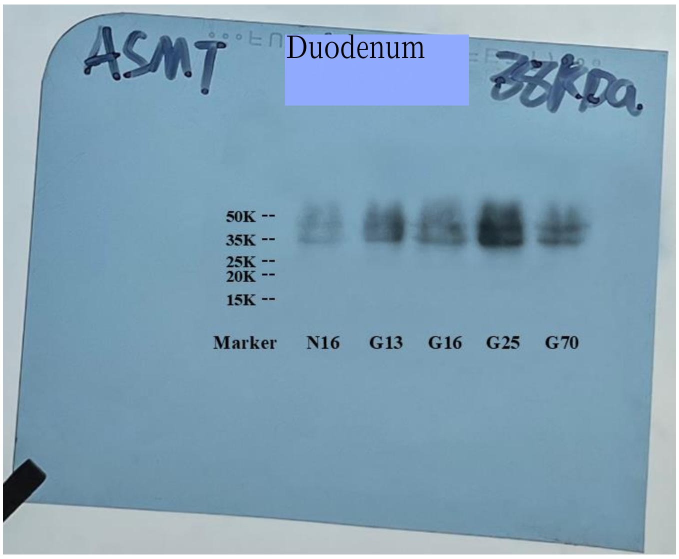
ASMT
Duodenum
38kDa
50K
35K
25K
20K
15K
Marker N16 G13 G16 G25 G70

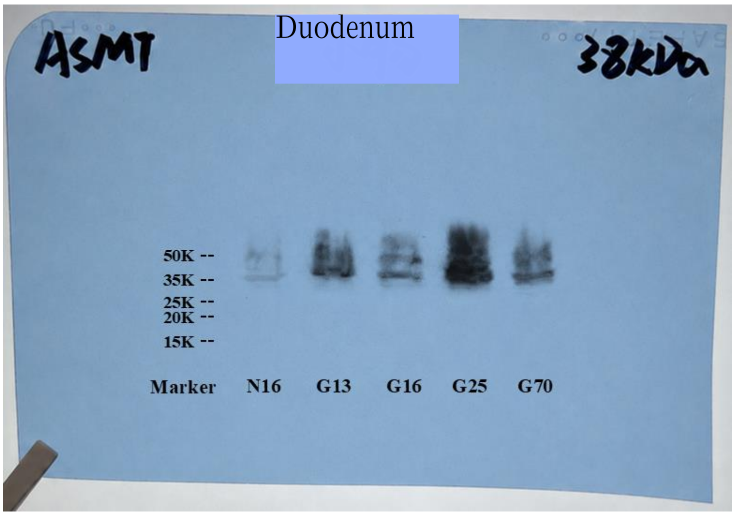
ASMT
Duodenum
38kDa
50K
35K
25K
20K
15K
Marker N16 G13 G16 G25 G70

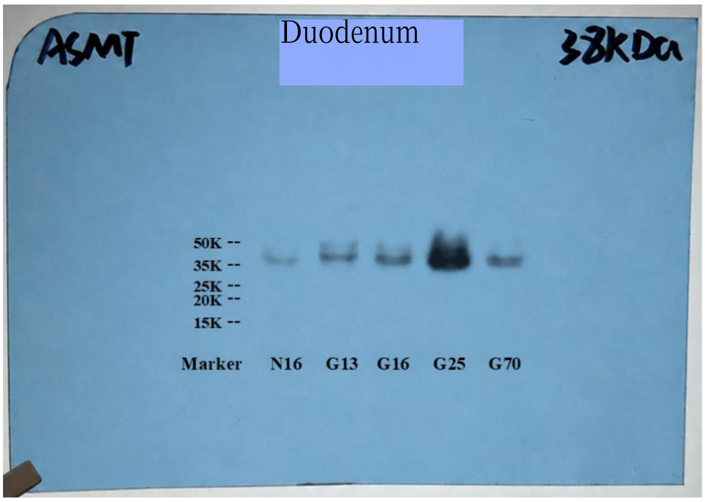
ASMT
Duodenum
38kDa
50K
35K
25K
20K
15K
Marker N16 G13 G16 G25 G70

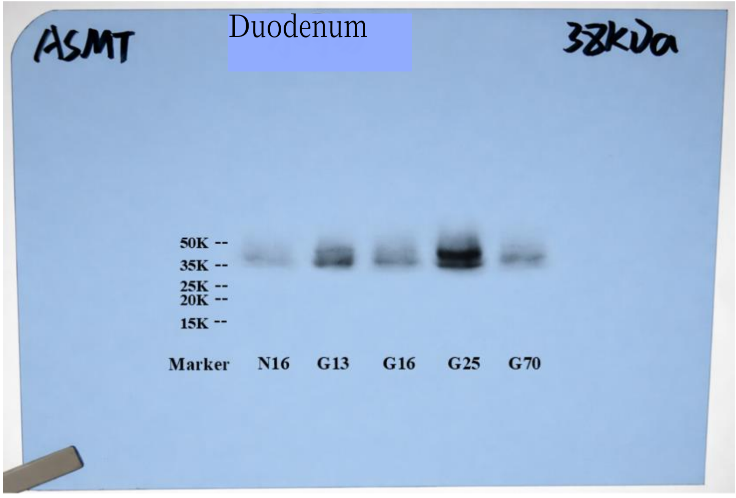
ASMT
Duodenum
38kDa
50K
35K
25K
20K
15K
Marker N16 G13 G16 G25 G70

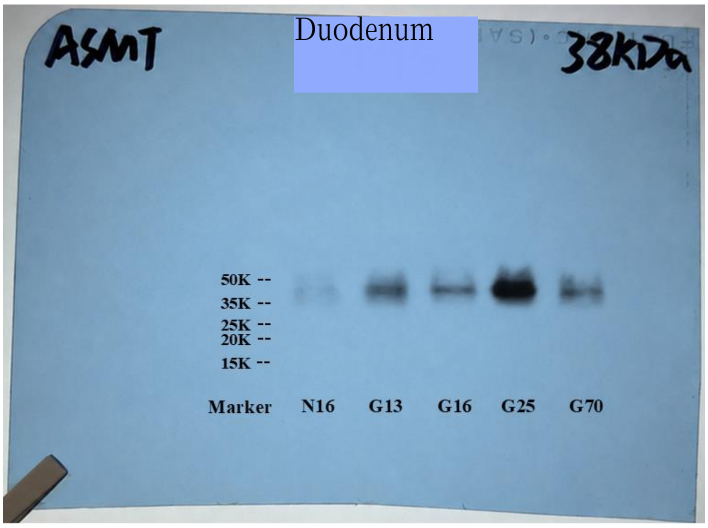
ASMT
Duodenum
38kDa
50K
35K
25K
20K
15K
Marker N16 G13 G16 G25 G70

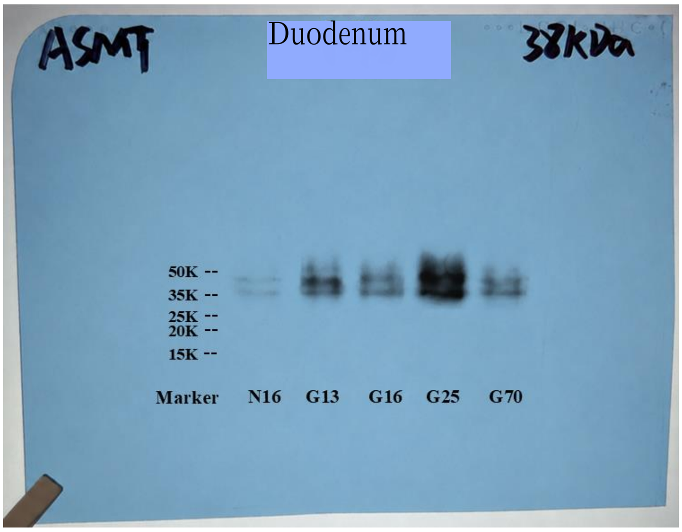
ASMT
Duodenum
38kDa
50K
35K
25K
20K
15K
Marker N16 G13 G16 G25 G70

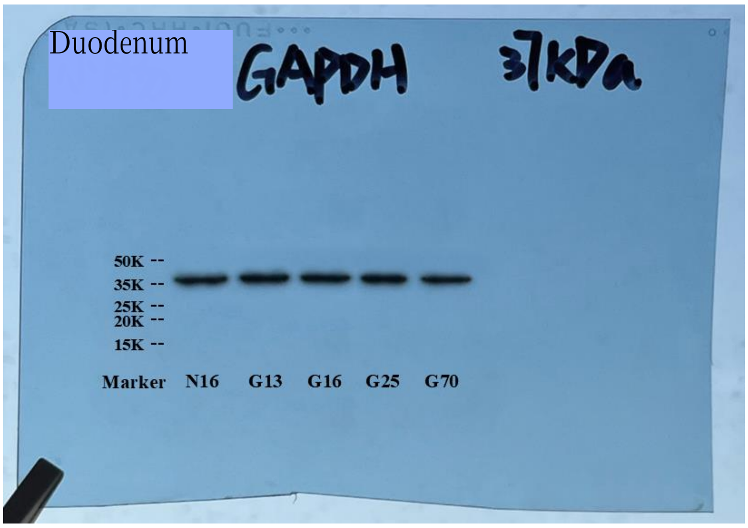
Duodenum
GAPDH
37kDa
50K
35K
25K
20K
15K
Marker
N16
G13
G16
G25
G70

7 Endometria

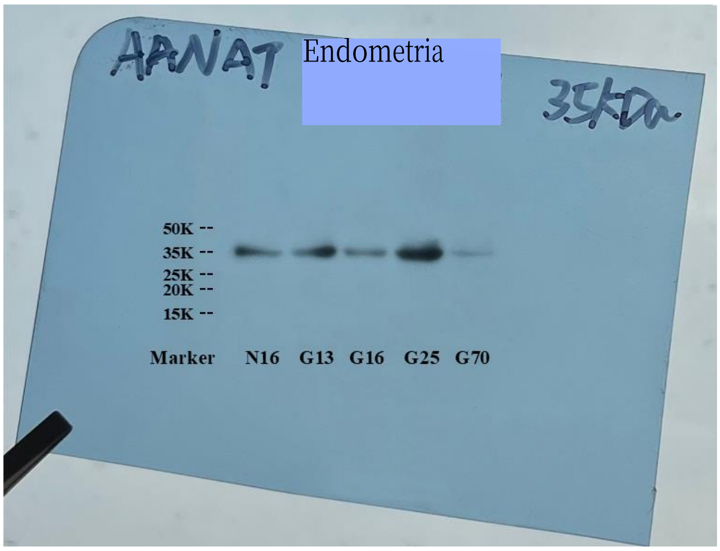

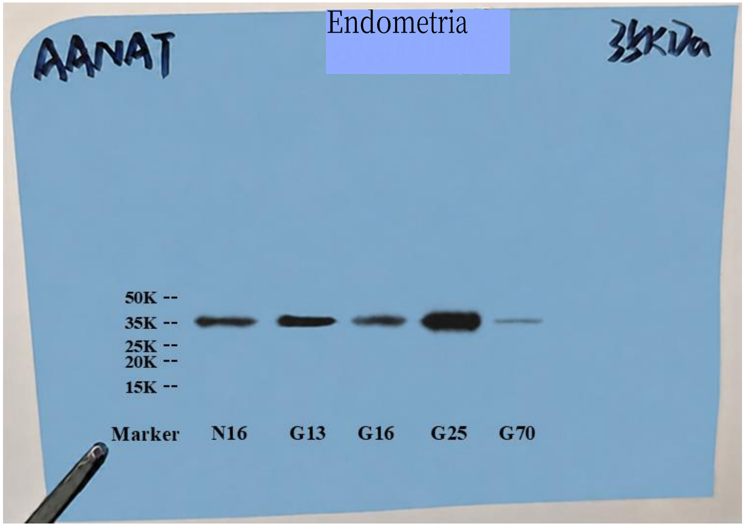

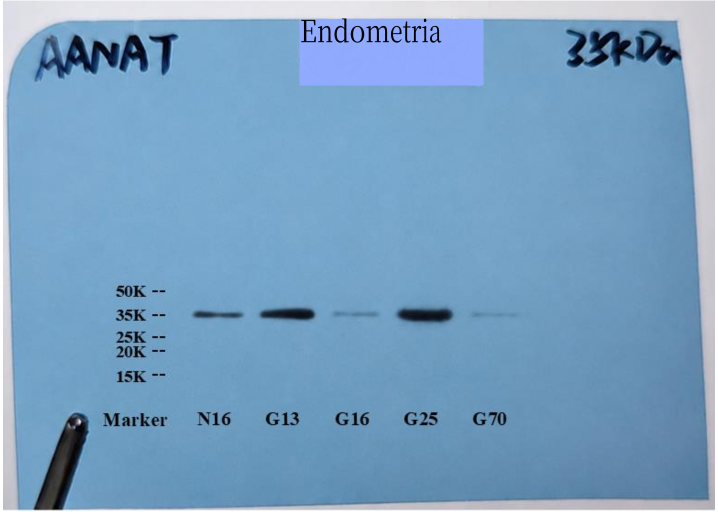

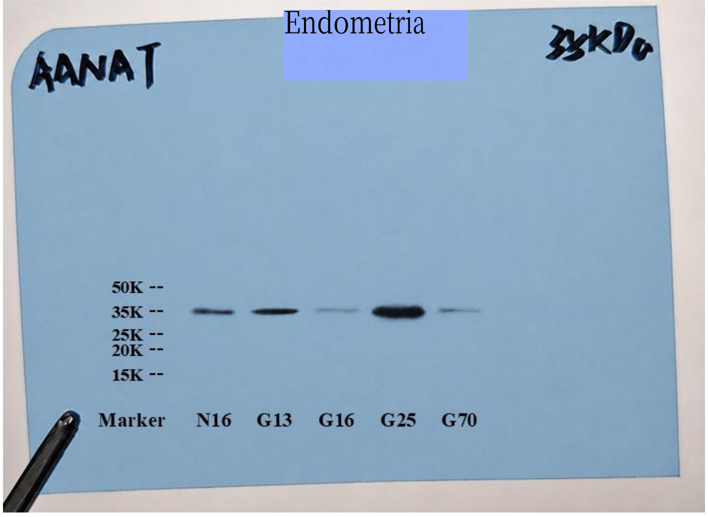

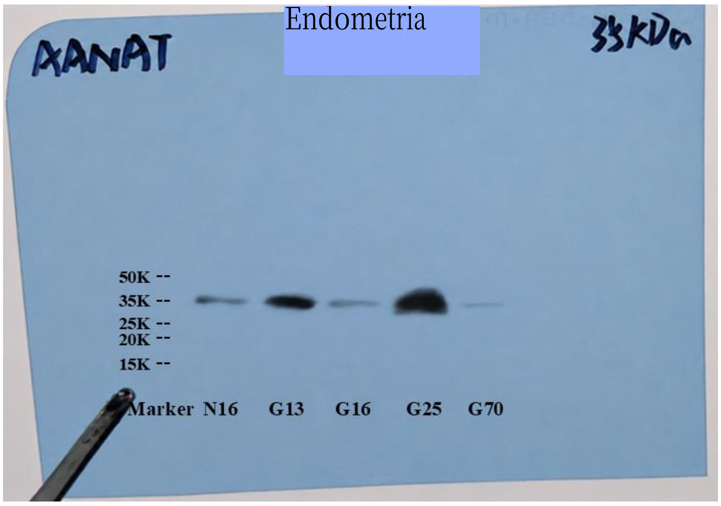

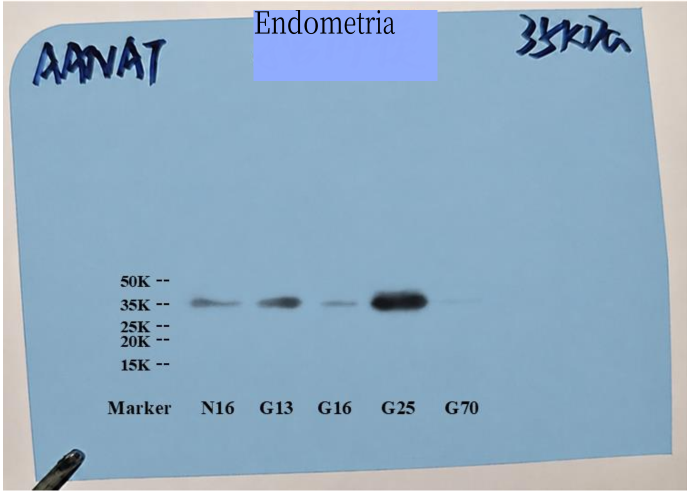

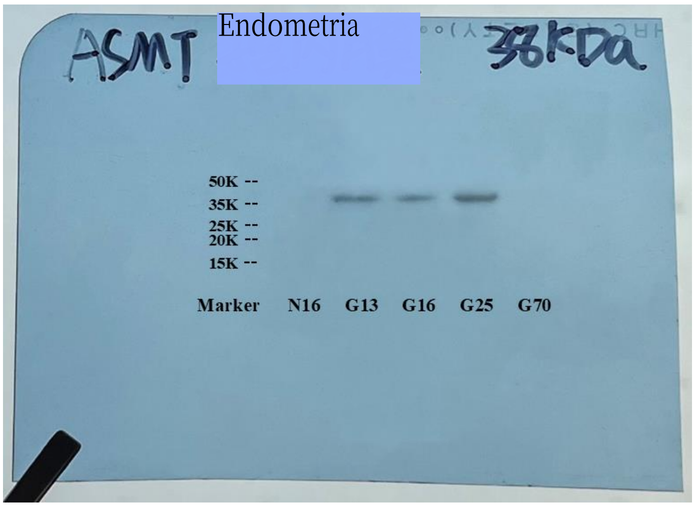
ASMT
Endometria
38KDa
50K --
35K --
25K --
20K --
15K --
Marker N16 G13 G16 G25 G70

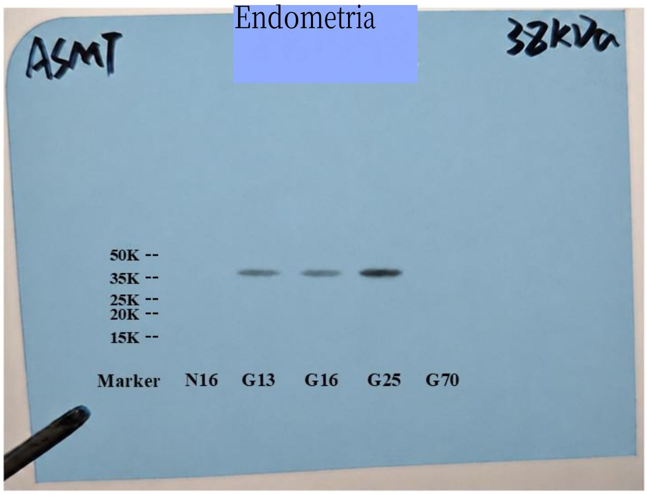
ASMT
Endometria
38kDa
50K --
35K --
25K --
20K --
15K --
Marker N16 G13 G16 G25 G70

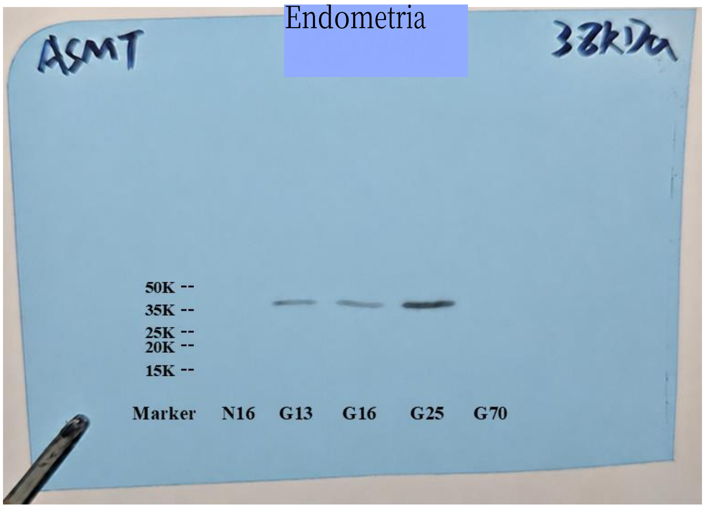
ASMT
Endometria
38kDa
50K --
35K --
25K --
20K --
15K --
Marker N16 G13 G16 G25 G70

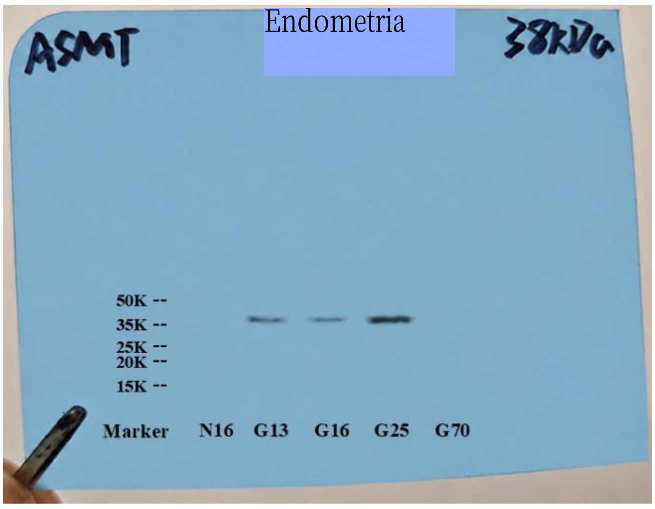
ASMT
Endometria
38kDa
50K --
35K --
25K --
20K --
15K --
Marker N16 G13 G16 G25 G70

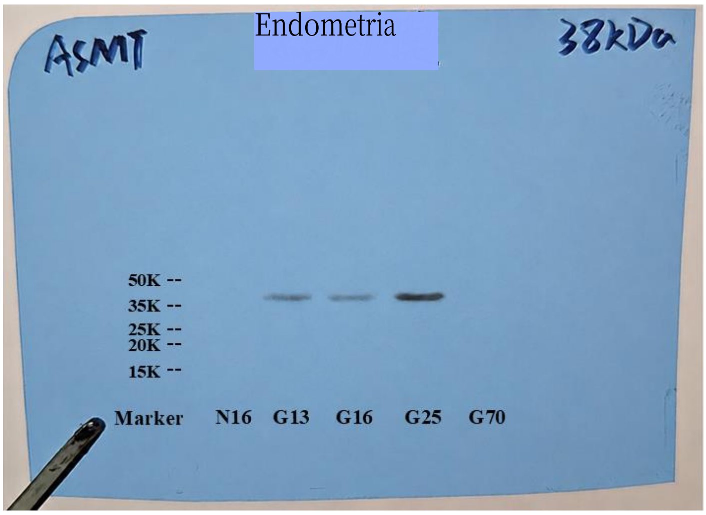
ASMT
Endometria
38kDa
50K --
35K --
25K --
20K --
15K --
Marker N16 G13 G16 G25 G70

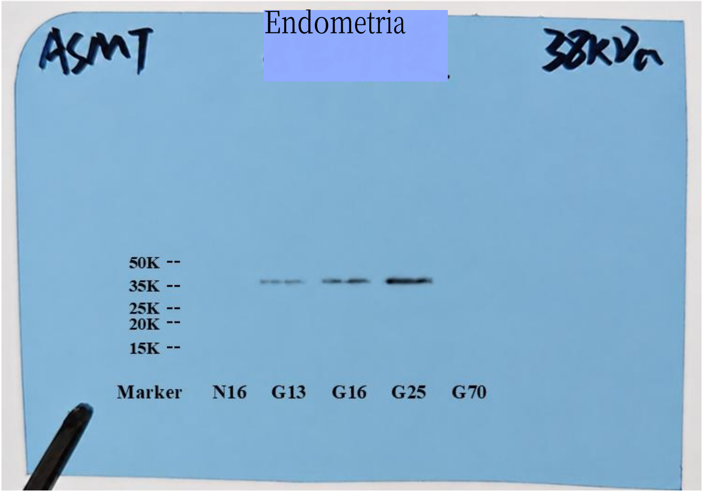
ASMT
Endometria
38kDa
50K --
35K --
25K --
20K --
15K --
Marker N16 G13 G16 G25 G70

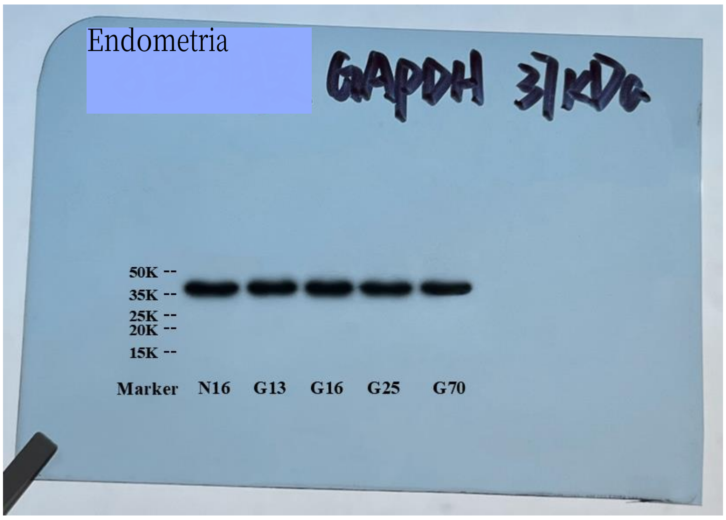
Endometria
GAPDH 37kDa
50K
35K
25K
20K
15K
Marker
N16
G13
G16
G25
G70
