# Supplementary material for: Expression of Rate-Limiting Enzymes of Melatonin Synthesis in Several Extrapineal Organs During Pregnancy in Ewes
Source: Biomolecules. 2026 Jul 17;16(7):1047. doi: 10.3390/biom16071047 (PMC13406823; doi:10.3390/biom16071047)

Thymus

AANAT

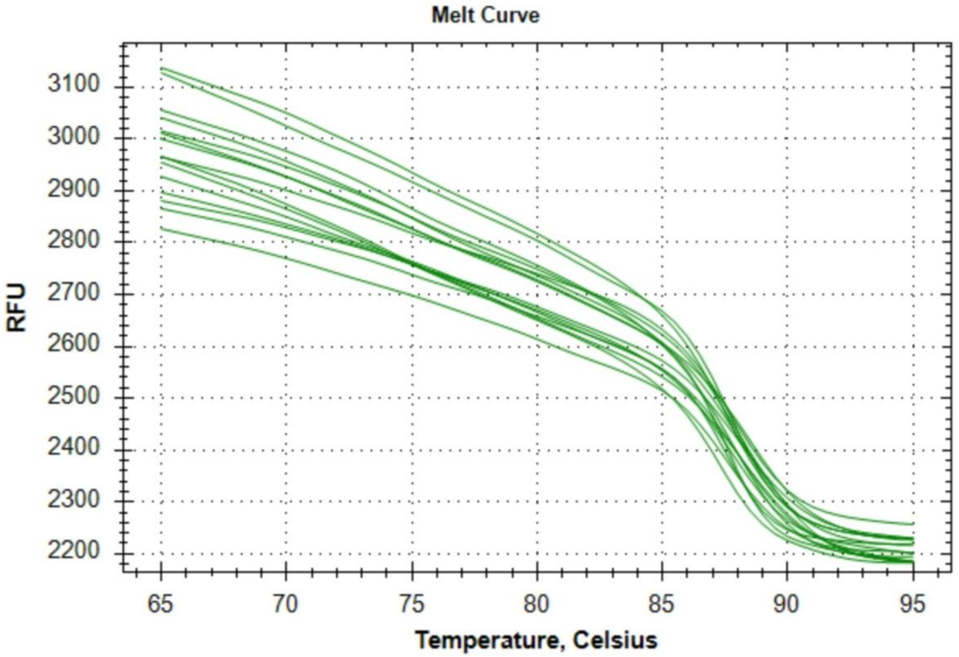

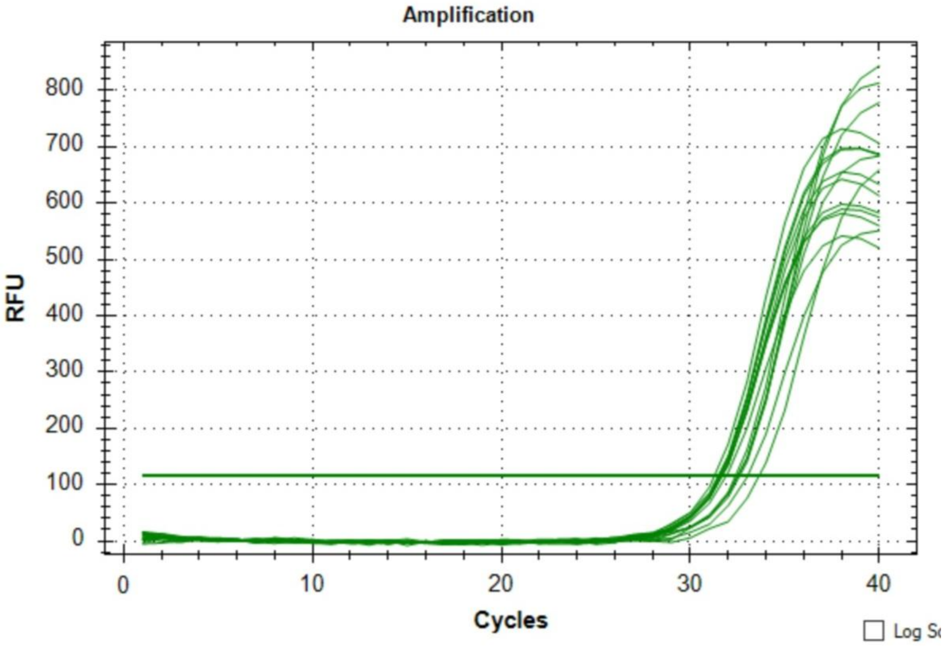

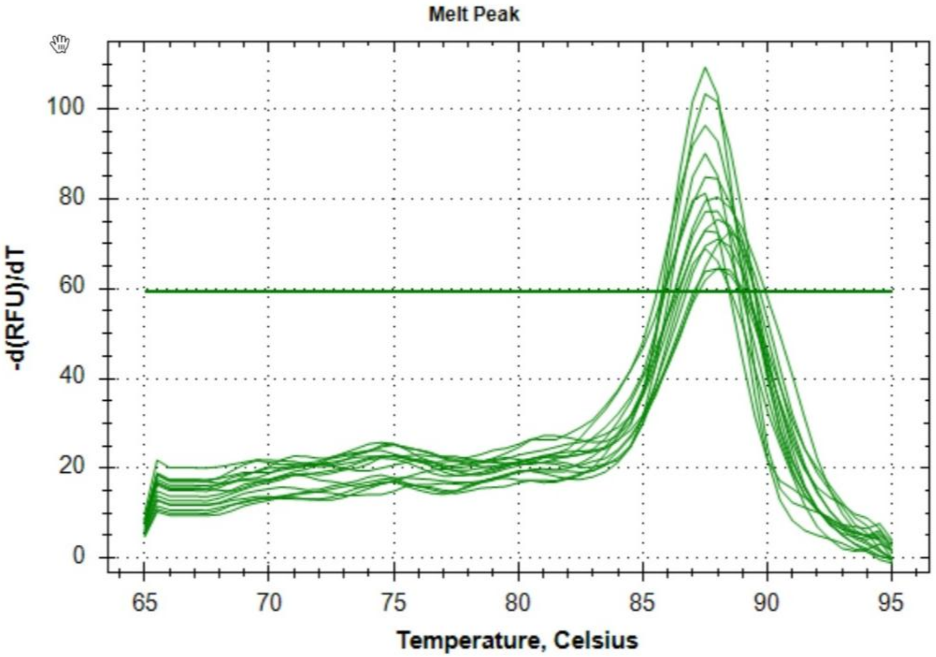

Lymph node

AANAT

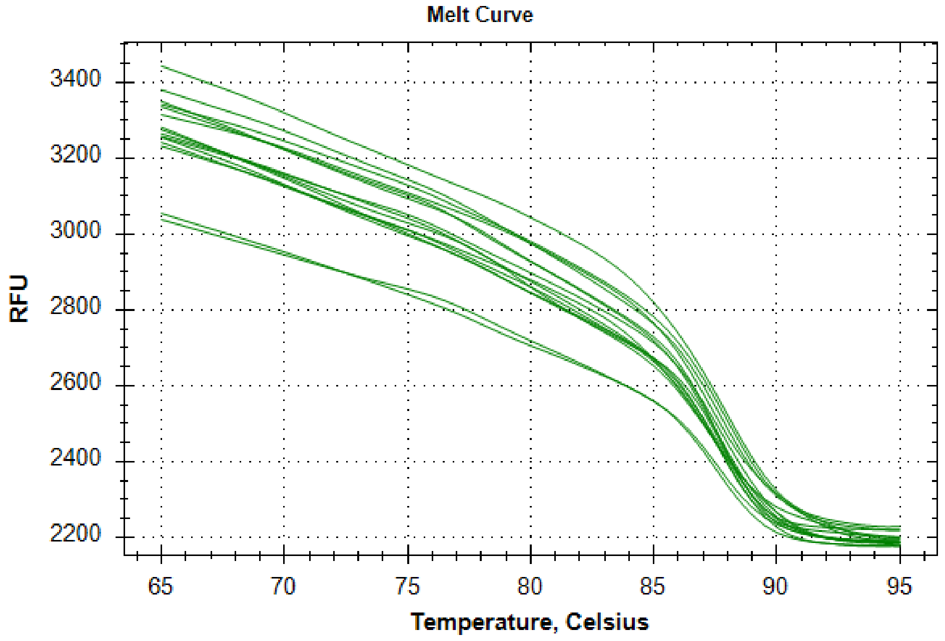

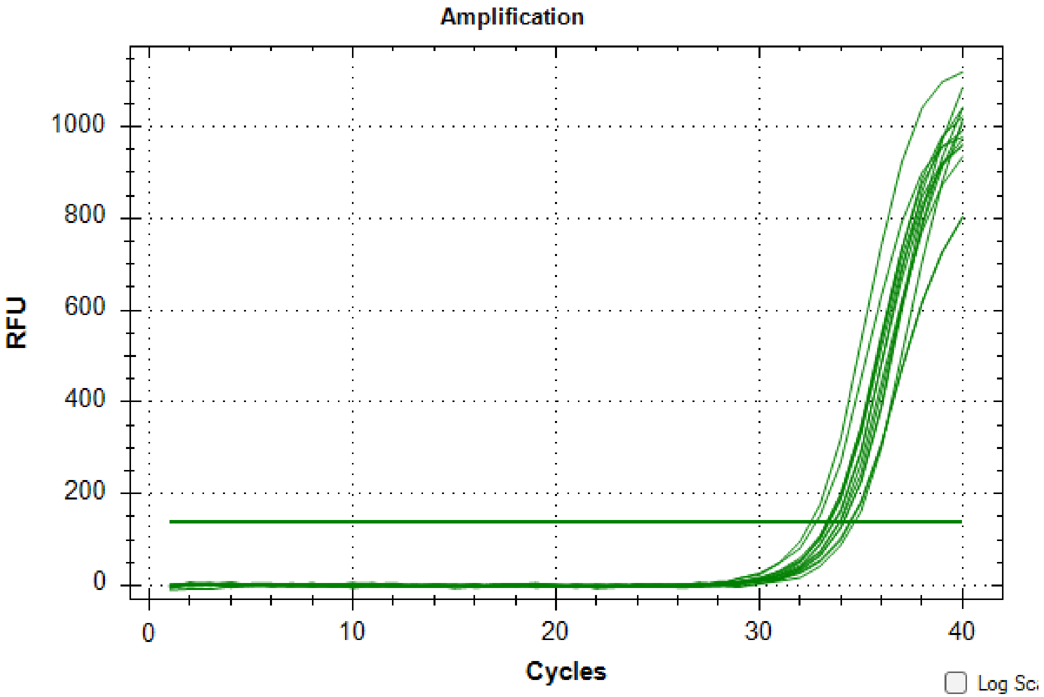

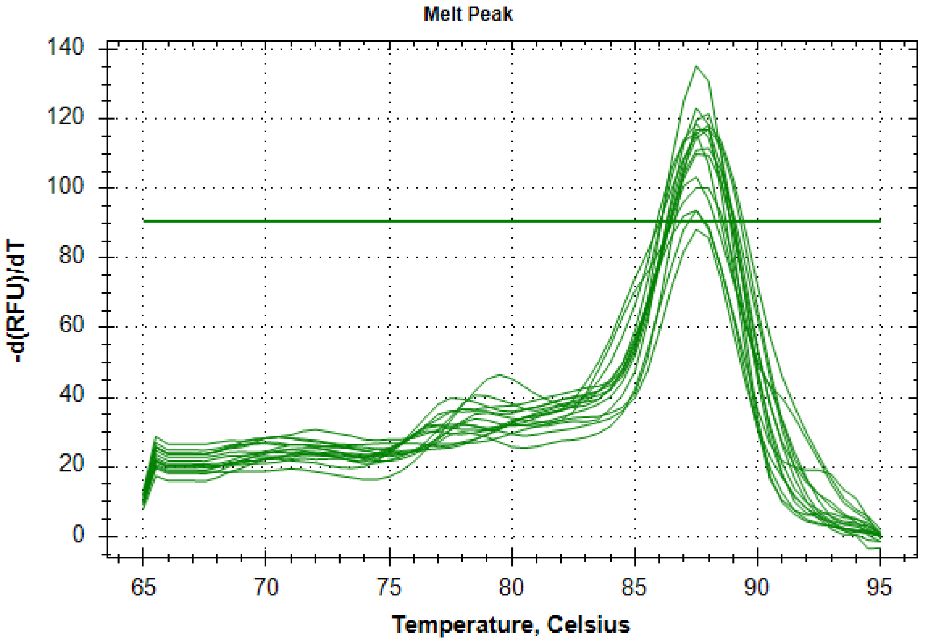

Spleen

AANAT

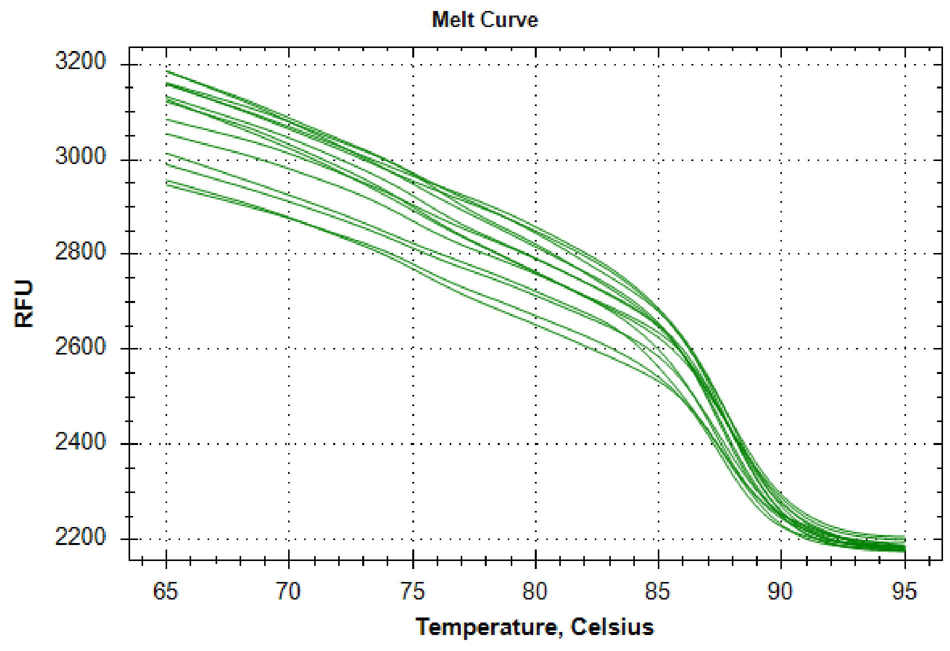

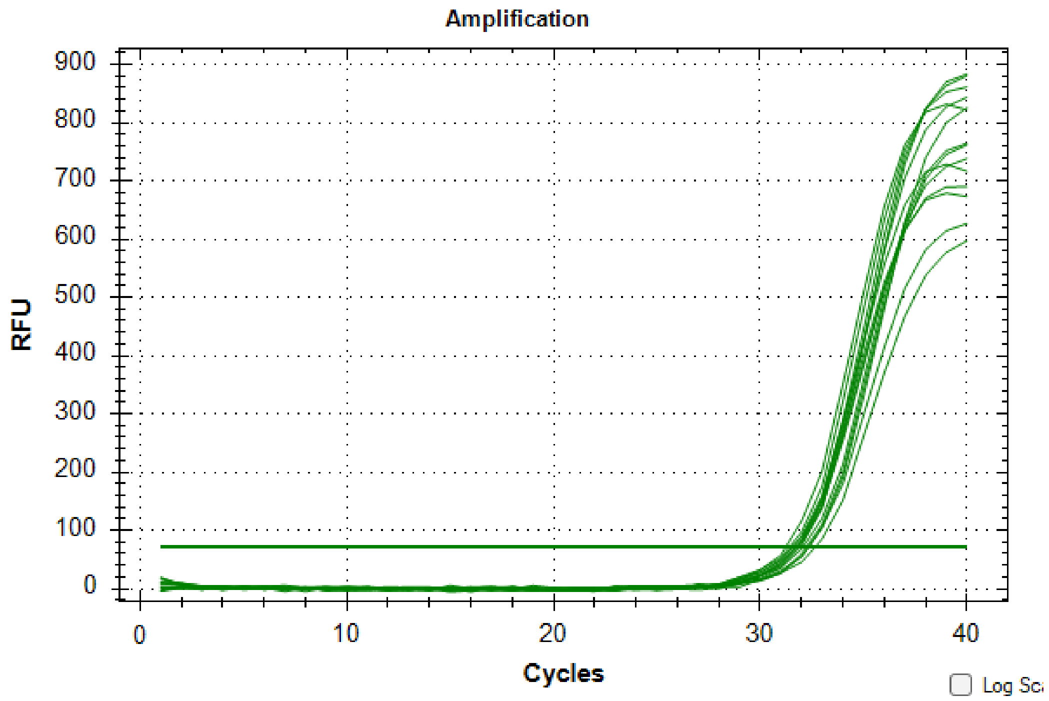

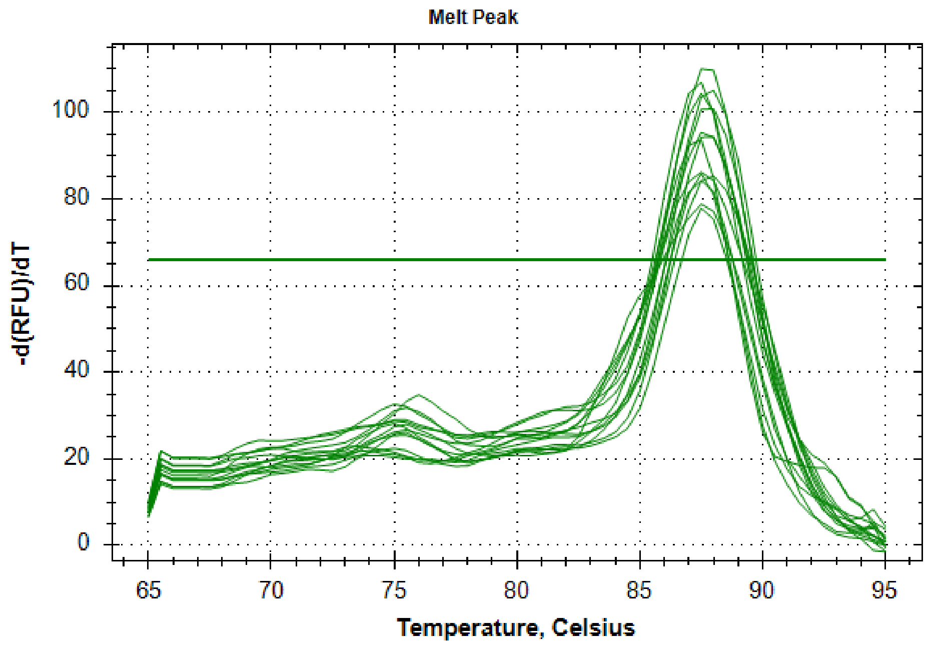

Liver

AANAT

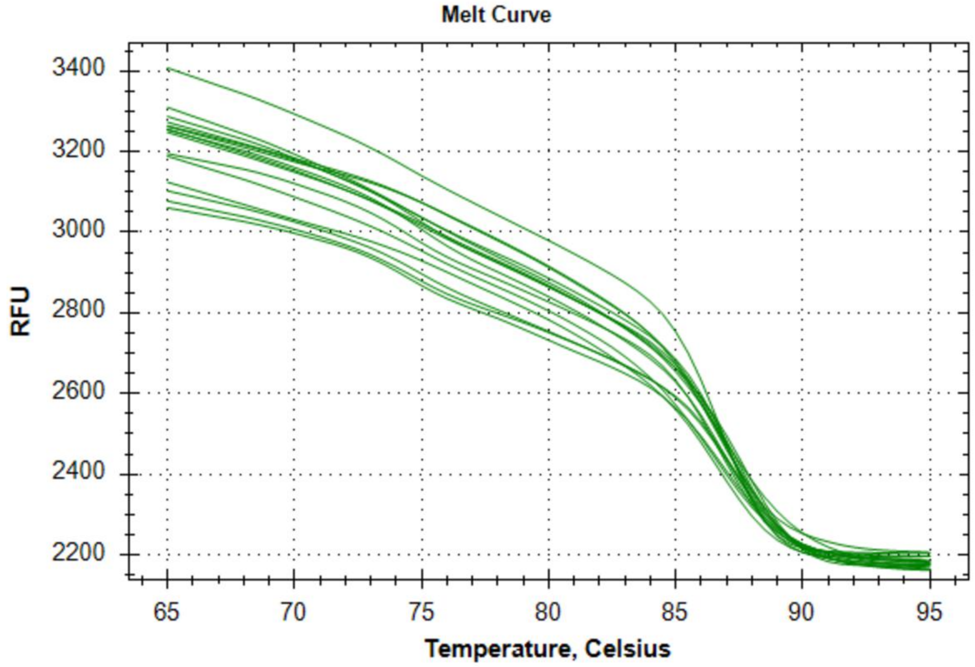
Melt Curve
RFU
3400
3200
3000
2800
2600
2400
2200
65
70
75
80
85
90
95
Temperature, Celsius

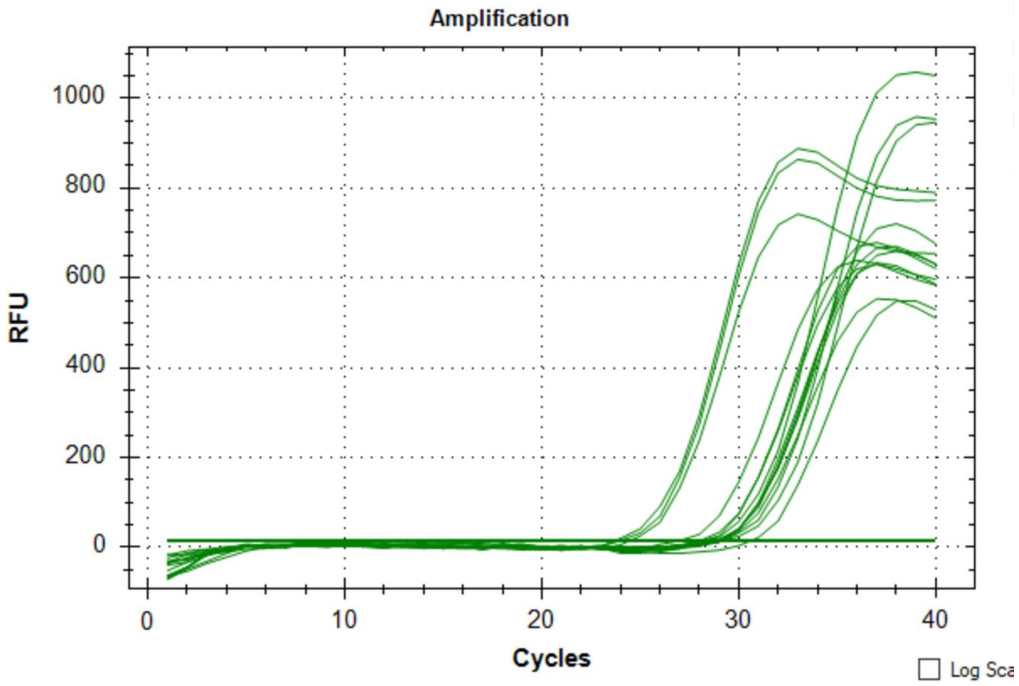
Amplification
RFU
1000
800
600
400
200
0
0
10
20
30
40
Cycles
Log Sca

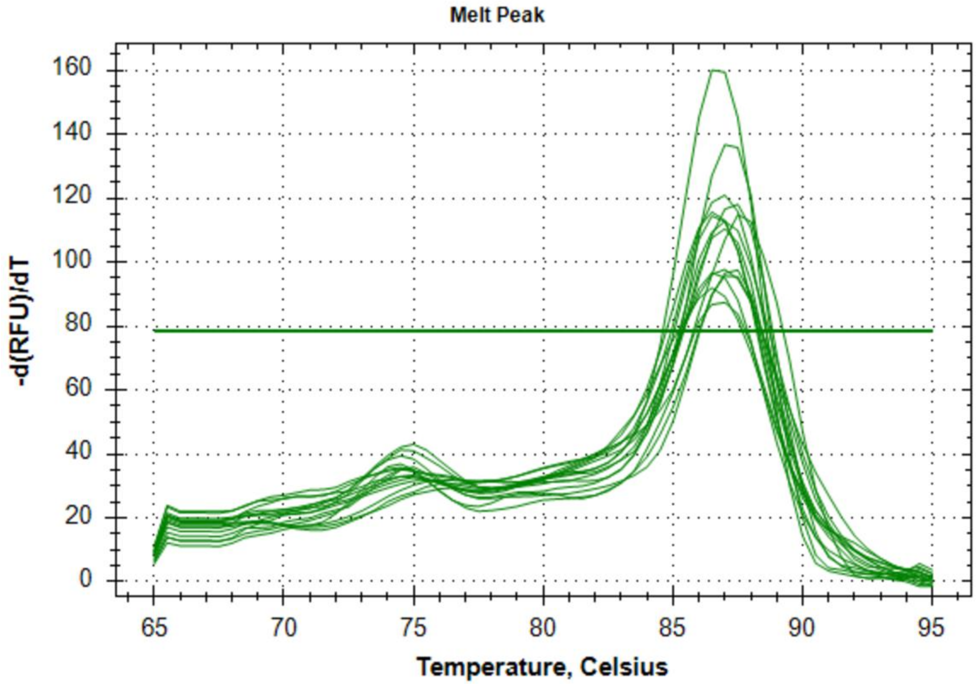
Melt Peak
-d(RFU)/dT
160
140
120
100
80
60
40
20
0
65
70
75
80
85
90
95
Temperature, Celsius

Thyroid

AANAT

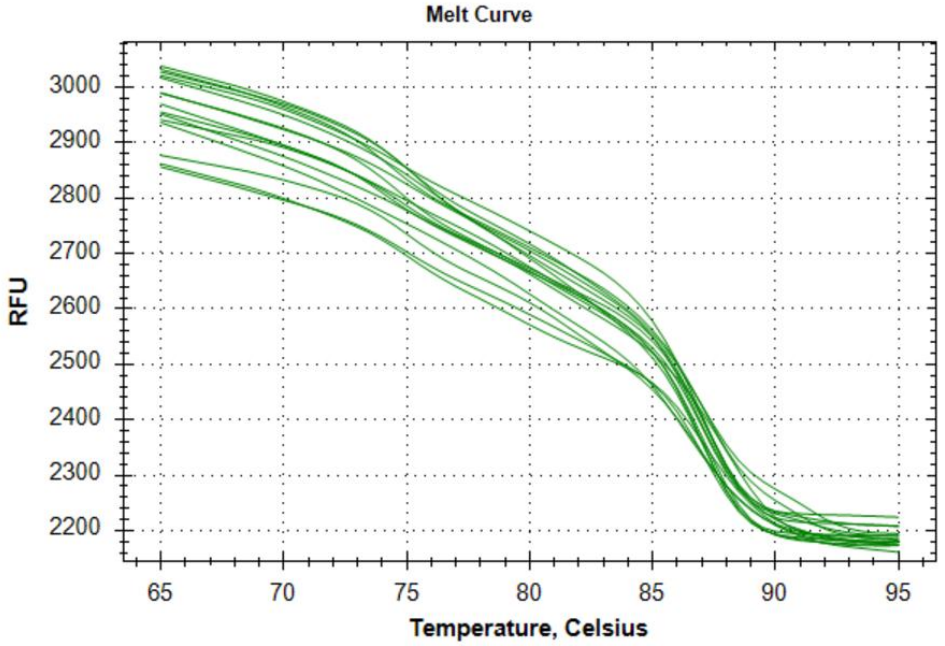

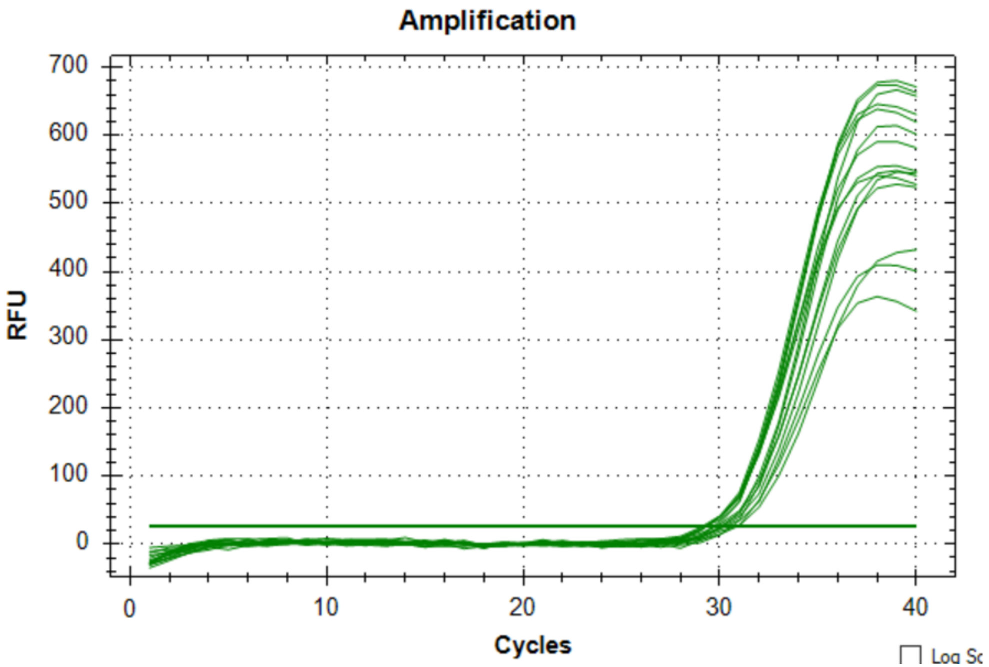

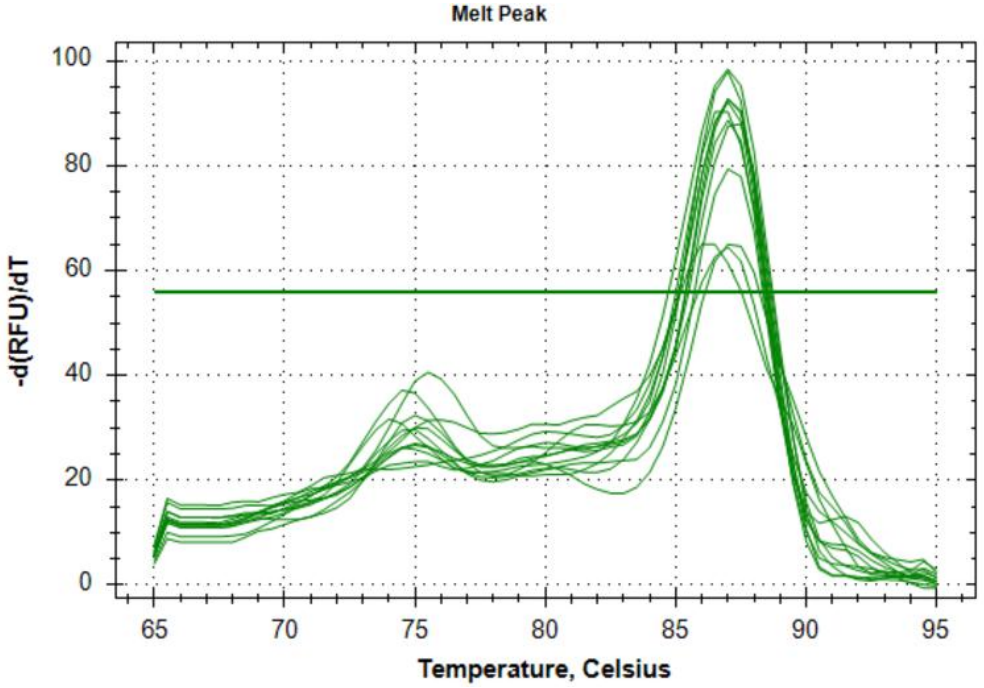

Duodenum

AANAT

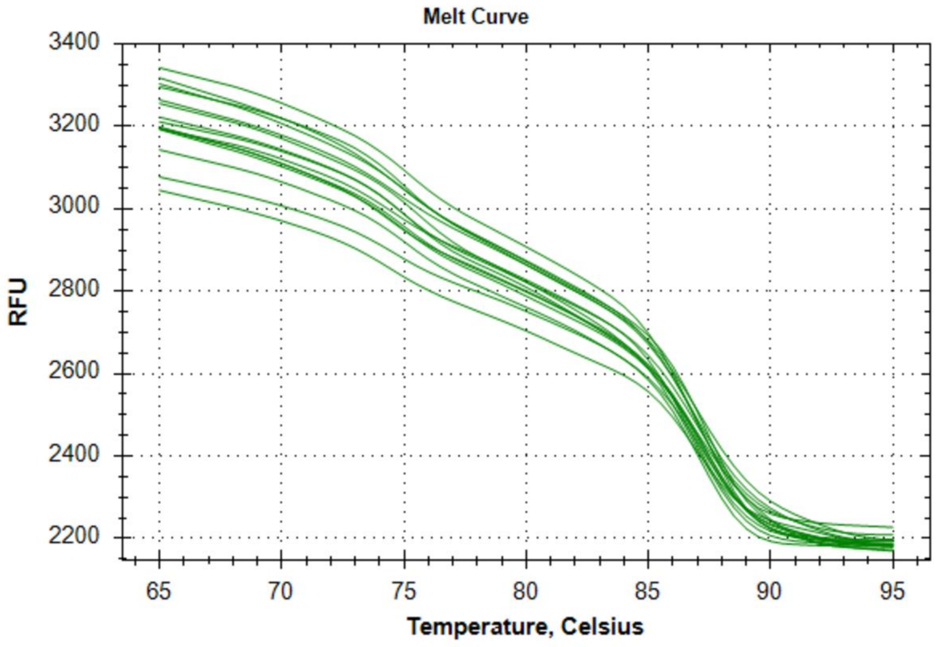

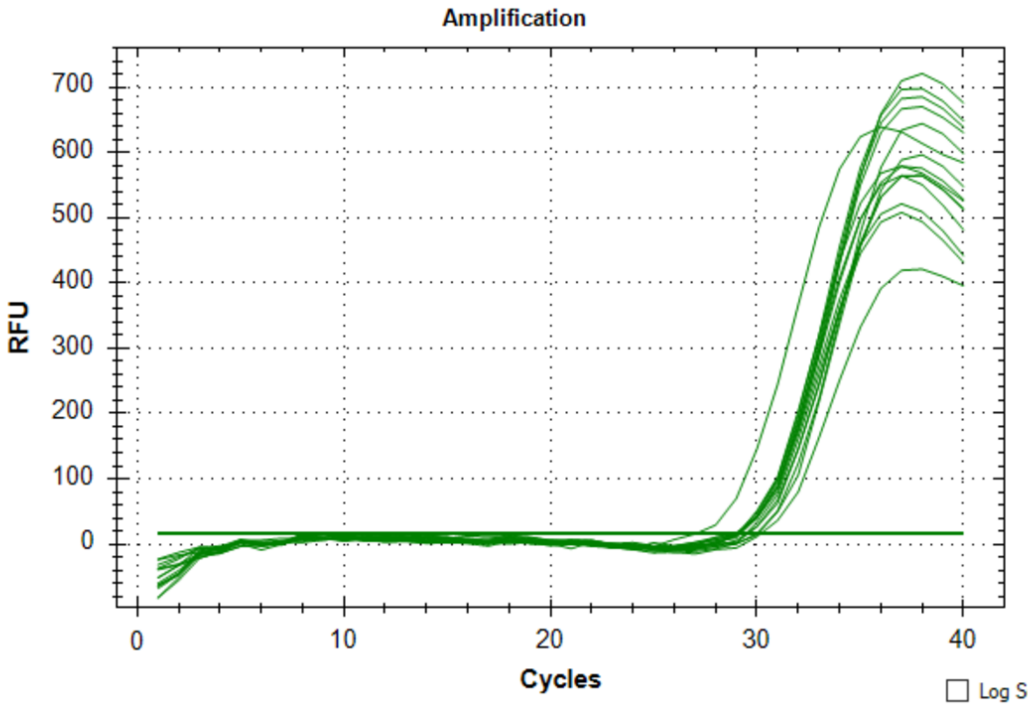

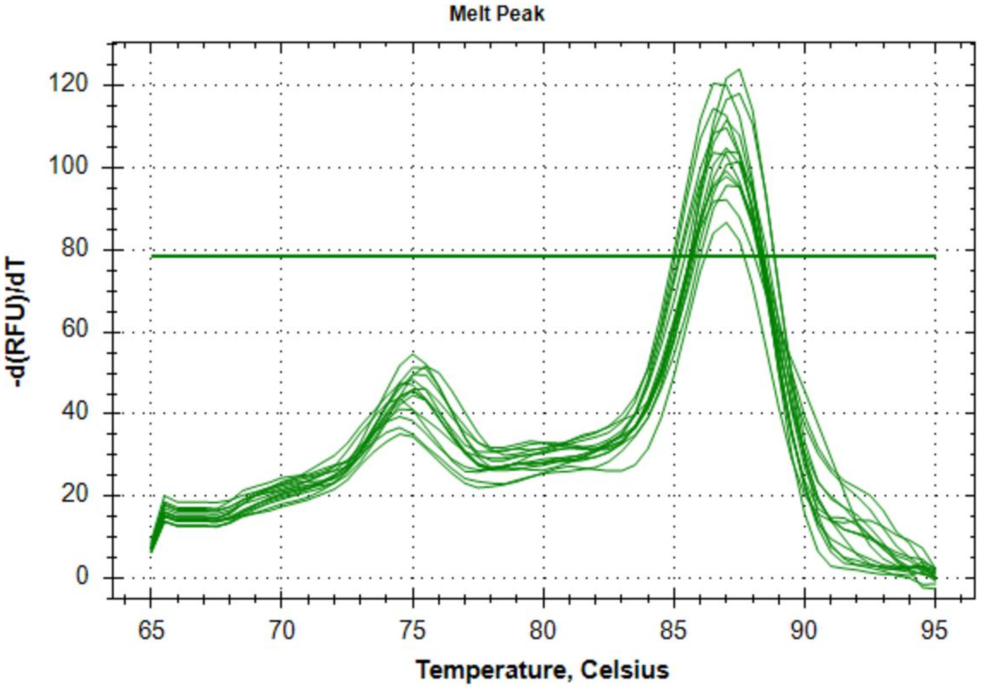

Endometrium

AANAT

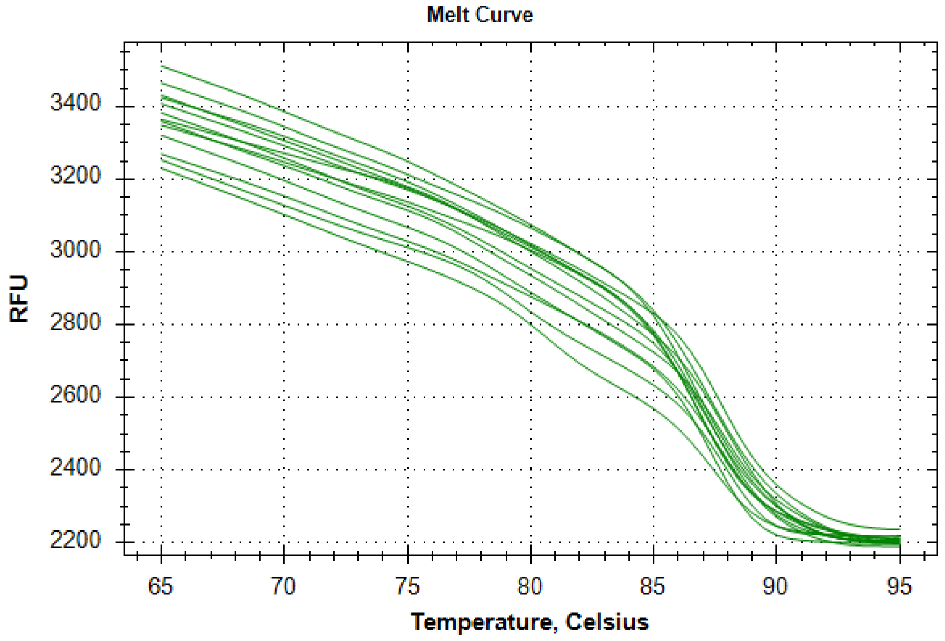

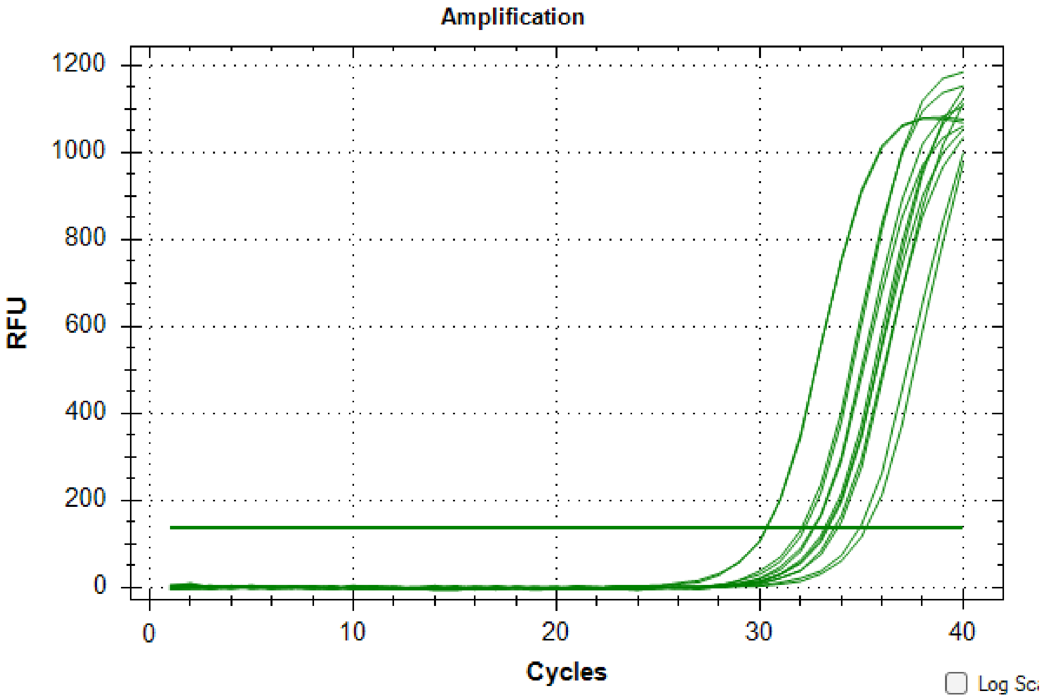

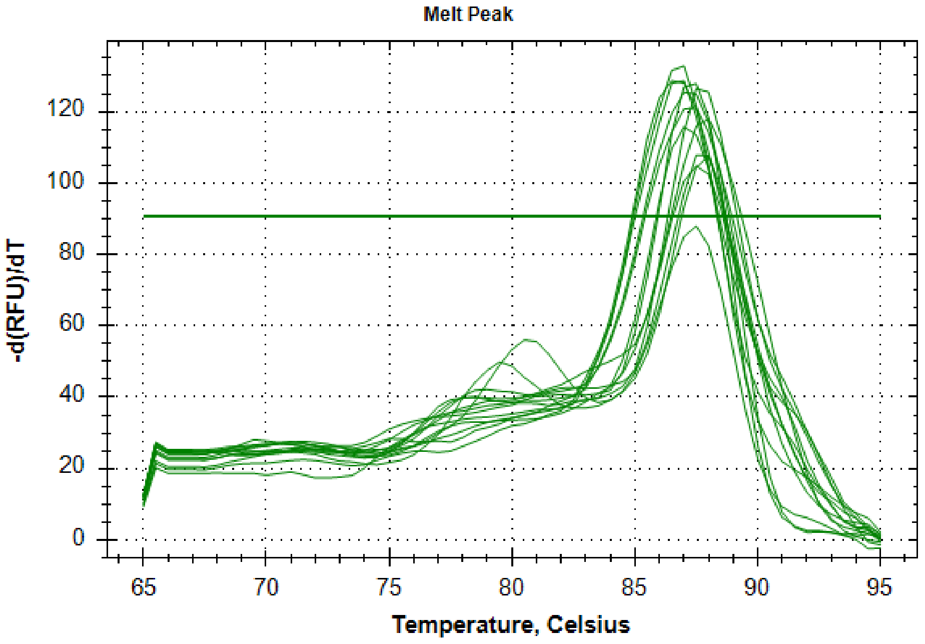

Thymus

ASMT

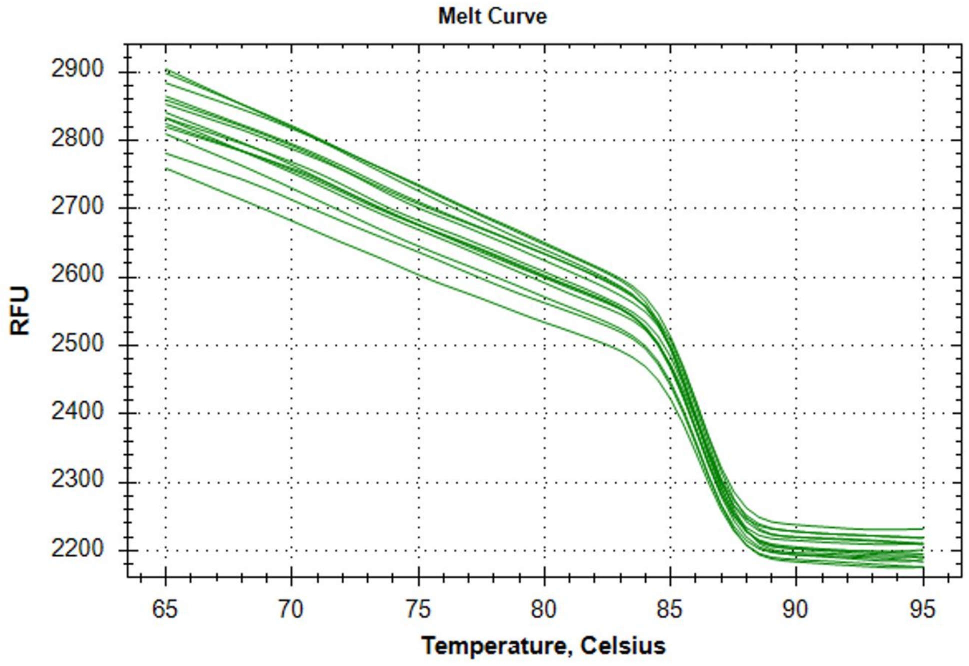

Melt Curve
2900
2800
2700
2600
2500
2400
2300
2200
RFU
65 70 75 80 85 90 95
Temperature, Celsius

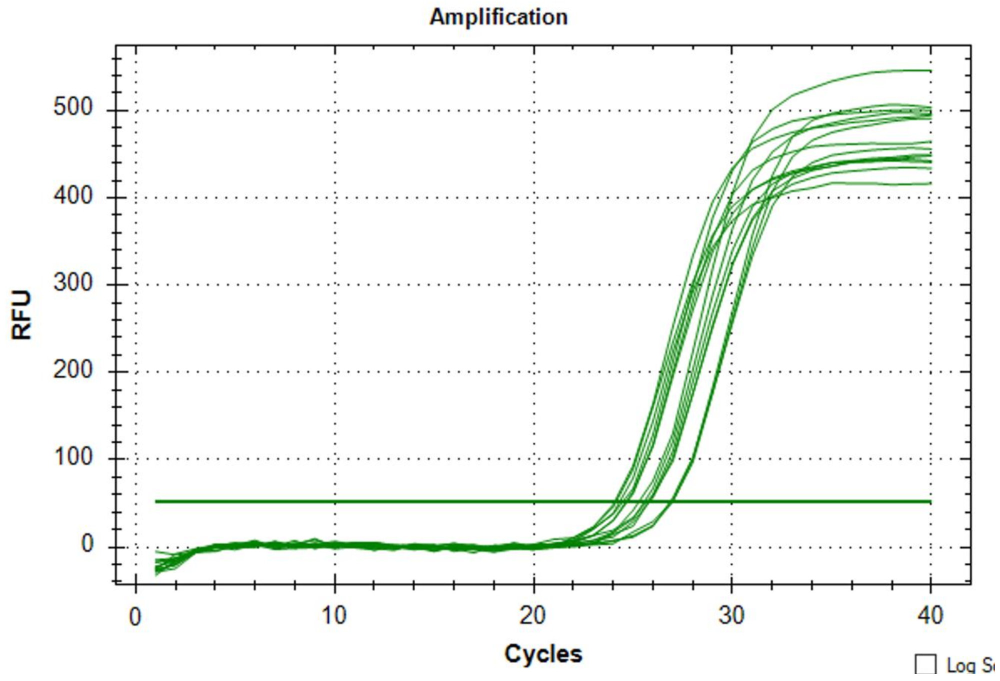

Amplification
500
400
300
200
100
0
RFU
0 10 20 30 40
Cycles

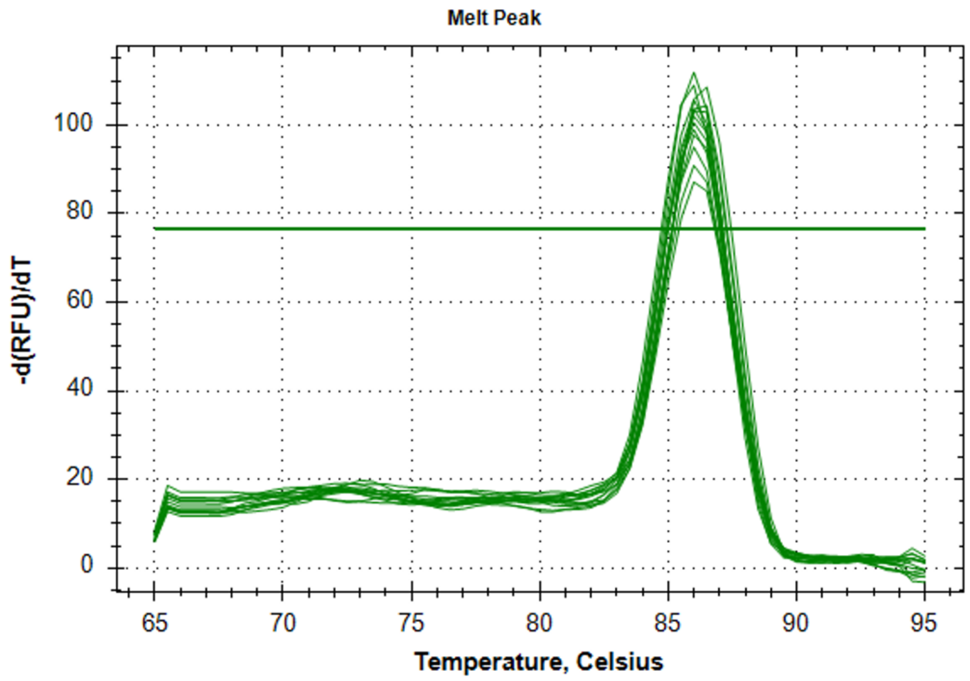

Melt Peak
100
80
60
40
20
0
-d(RFU)/dT
65 70 75 80 85 90 95
Temperature, Celsius

Lymph node

ASMT

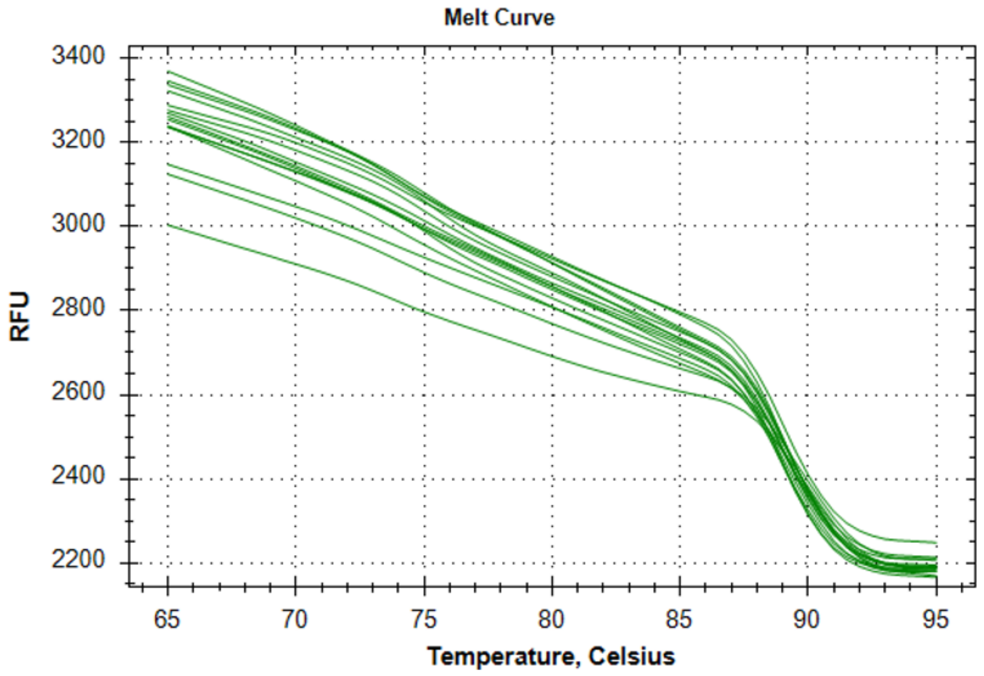

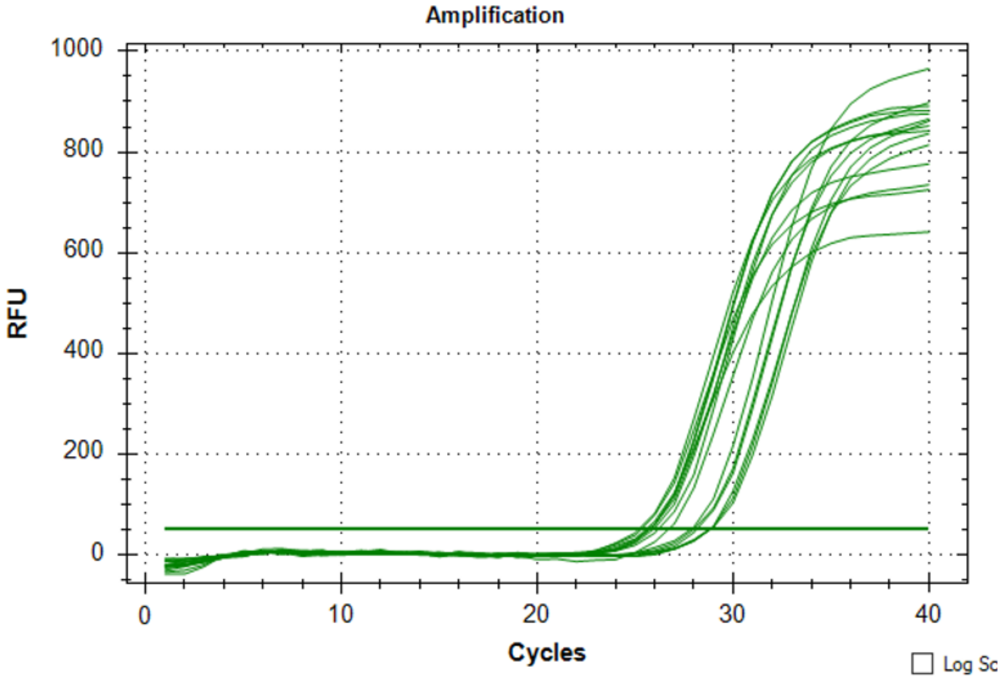

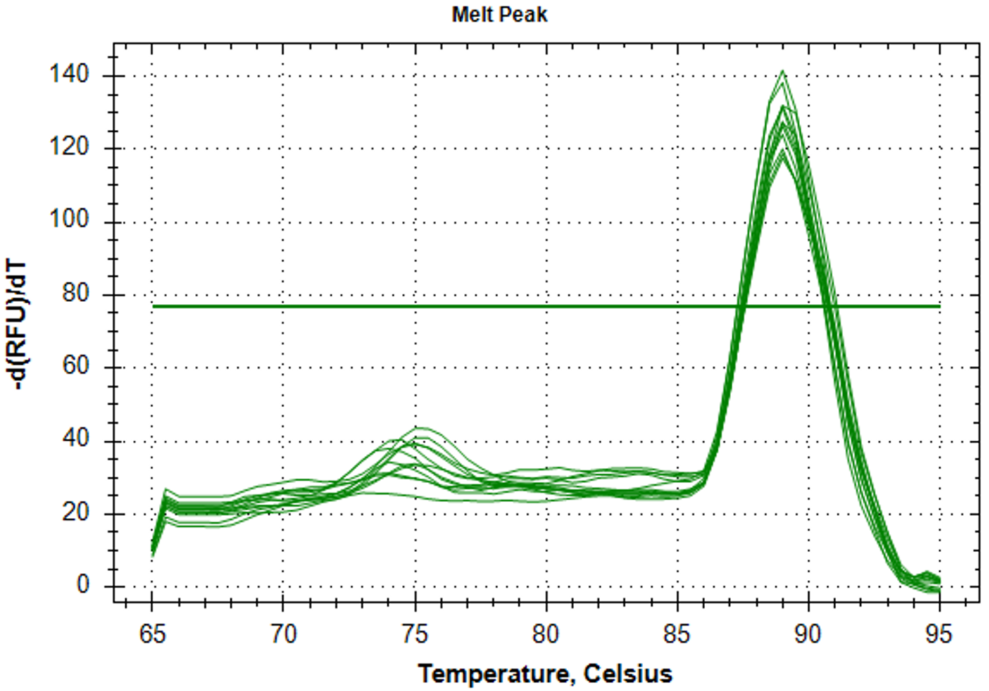

Spleen

ASMT

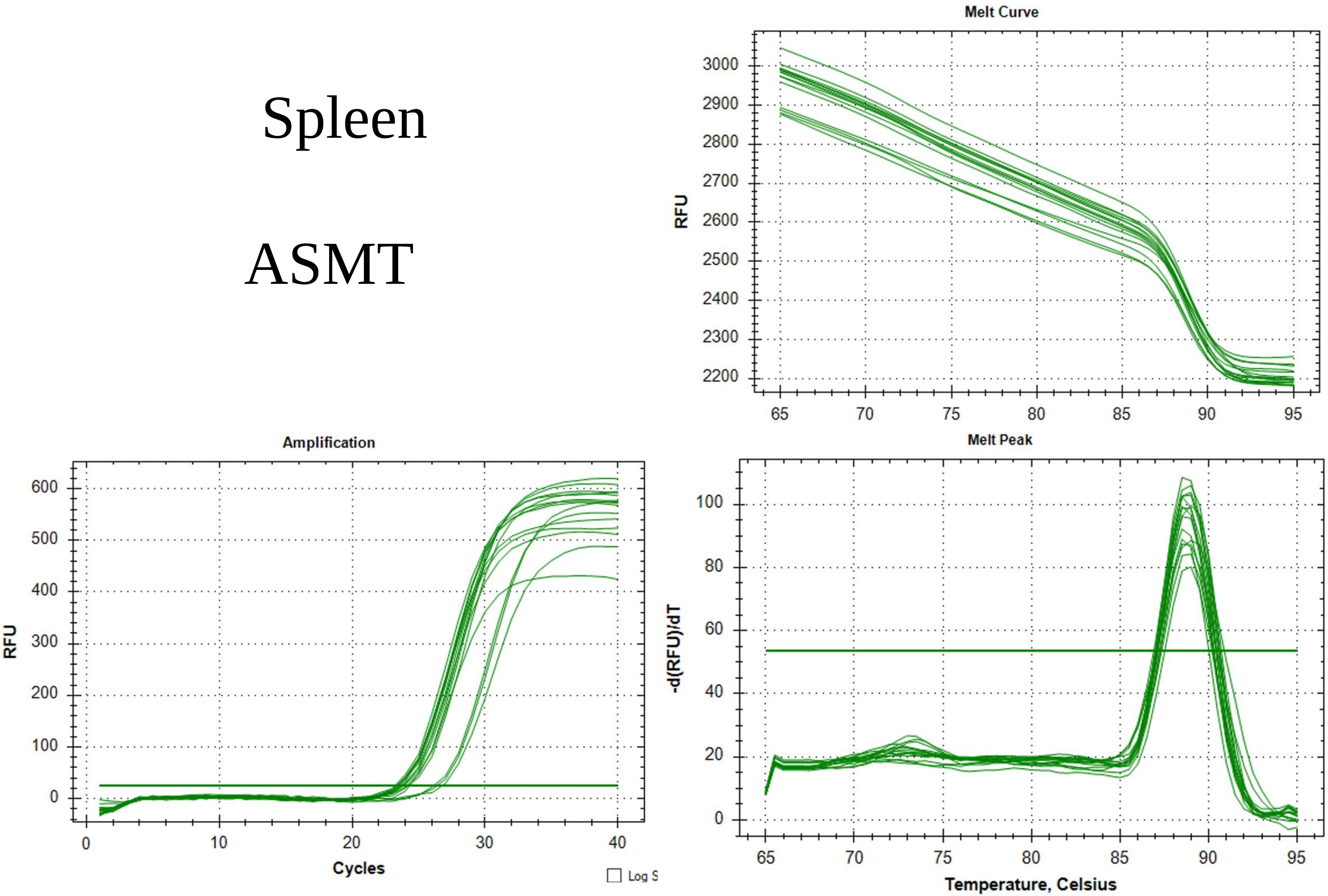
Melt Curve
RFU
3000
2900
2800
2700
2600
2500
2400
2300
2200
65
70
75
80
85
90
95
Amplification
RFU
600
500
400
300
200
100
0
0
10
20
30
40
Cycles
Log S
Melt Peak
-d(RFU)/dT
100
80
60
40
20
0
65
70
75
80
85
90
95
Temperature, Celsius

Liver

ASMT

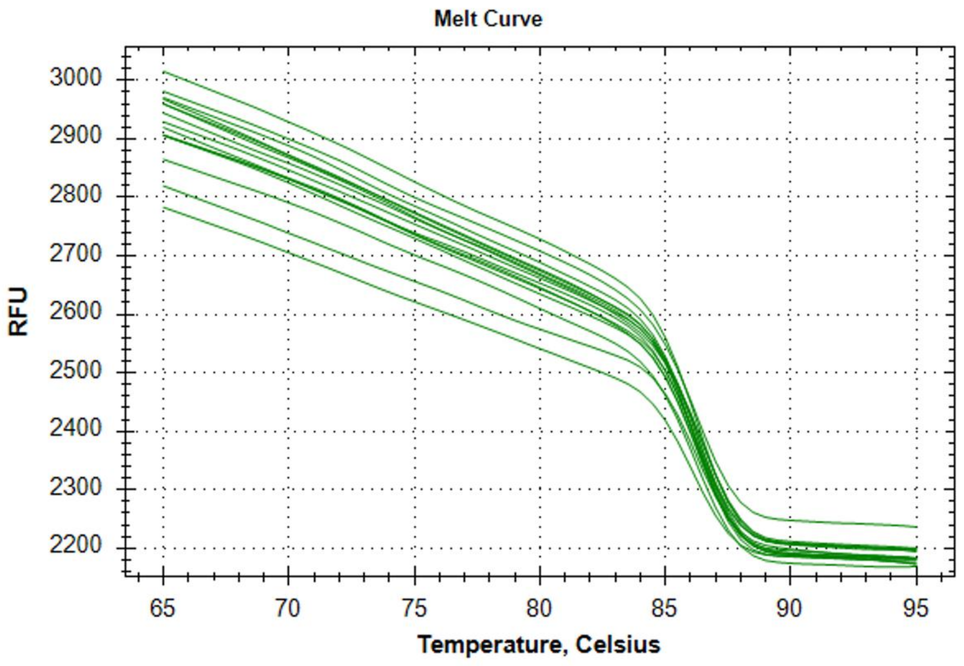
Melt Curve
RFU
3000
2900
2800
2700
2600
2500
2400
2300
2200
65
70
75
80
85
90
95
Temperature, Celsius

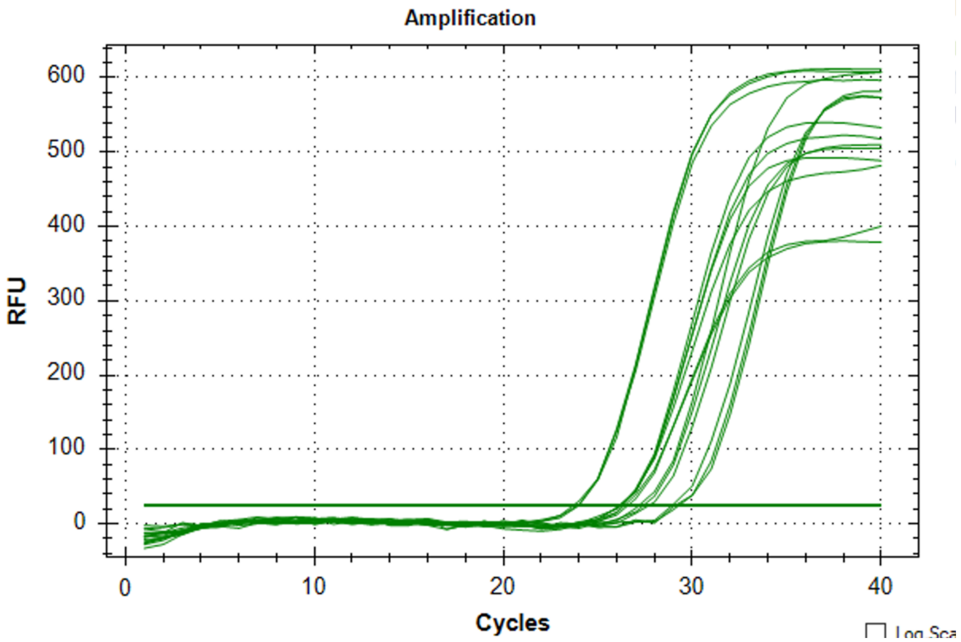
Amplification
RFU
600
500
400
300
200
100
0
0
10
20
30
40
Cycles

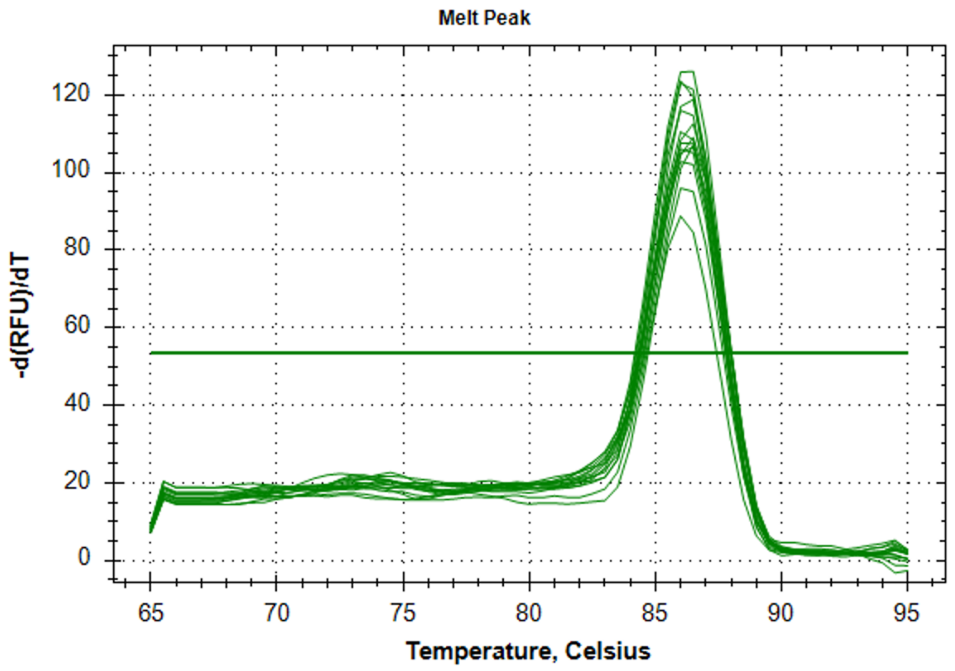
Melt Peak
-d(RFU)/dT
120
100
80
60
40
20
0
65
70
75
80
85
90
95
Temperature, Celsius

Thyroid

ASMT

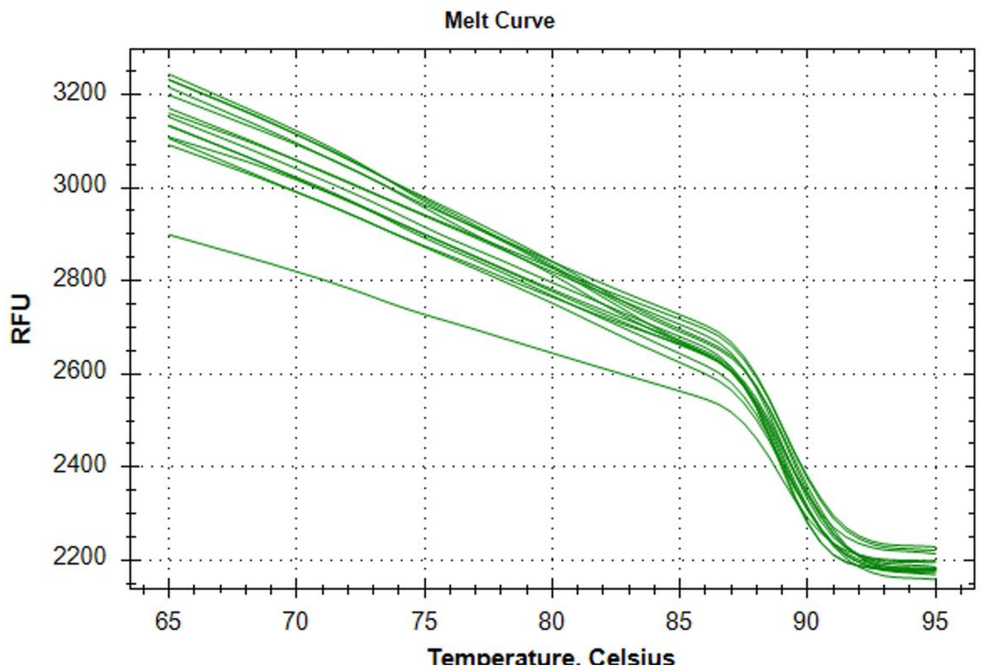

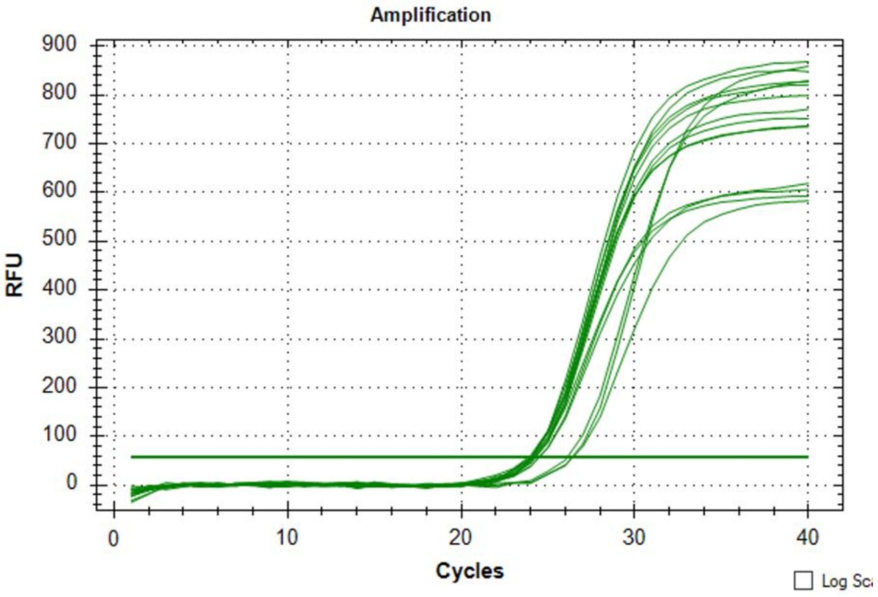

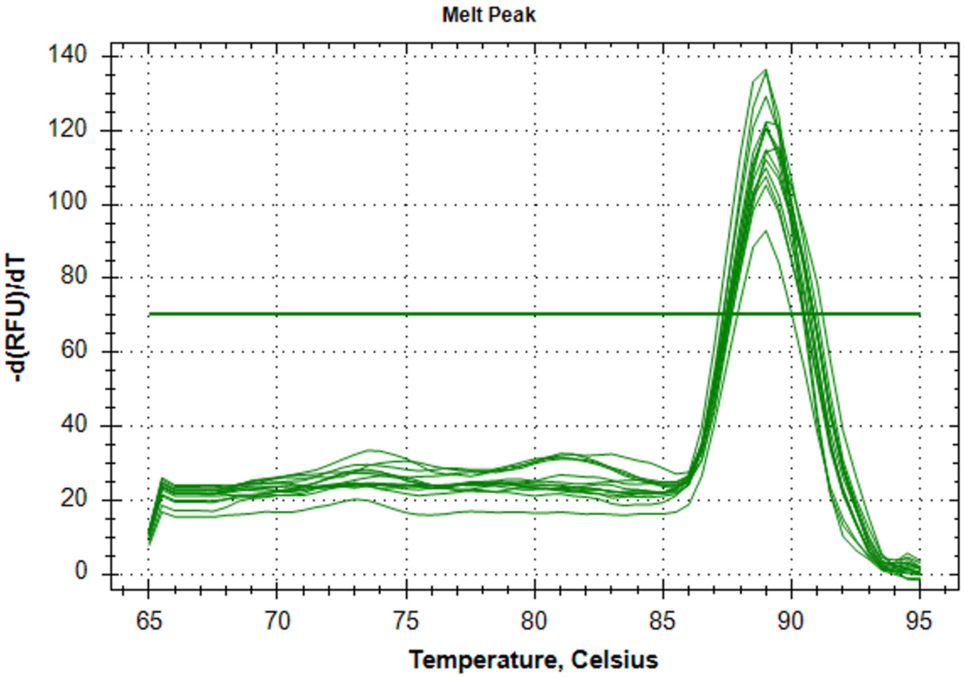

Duodenum

ASMT

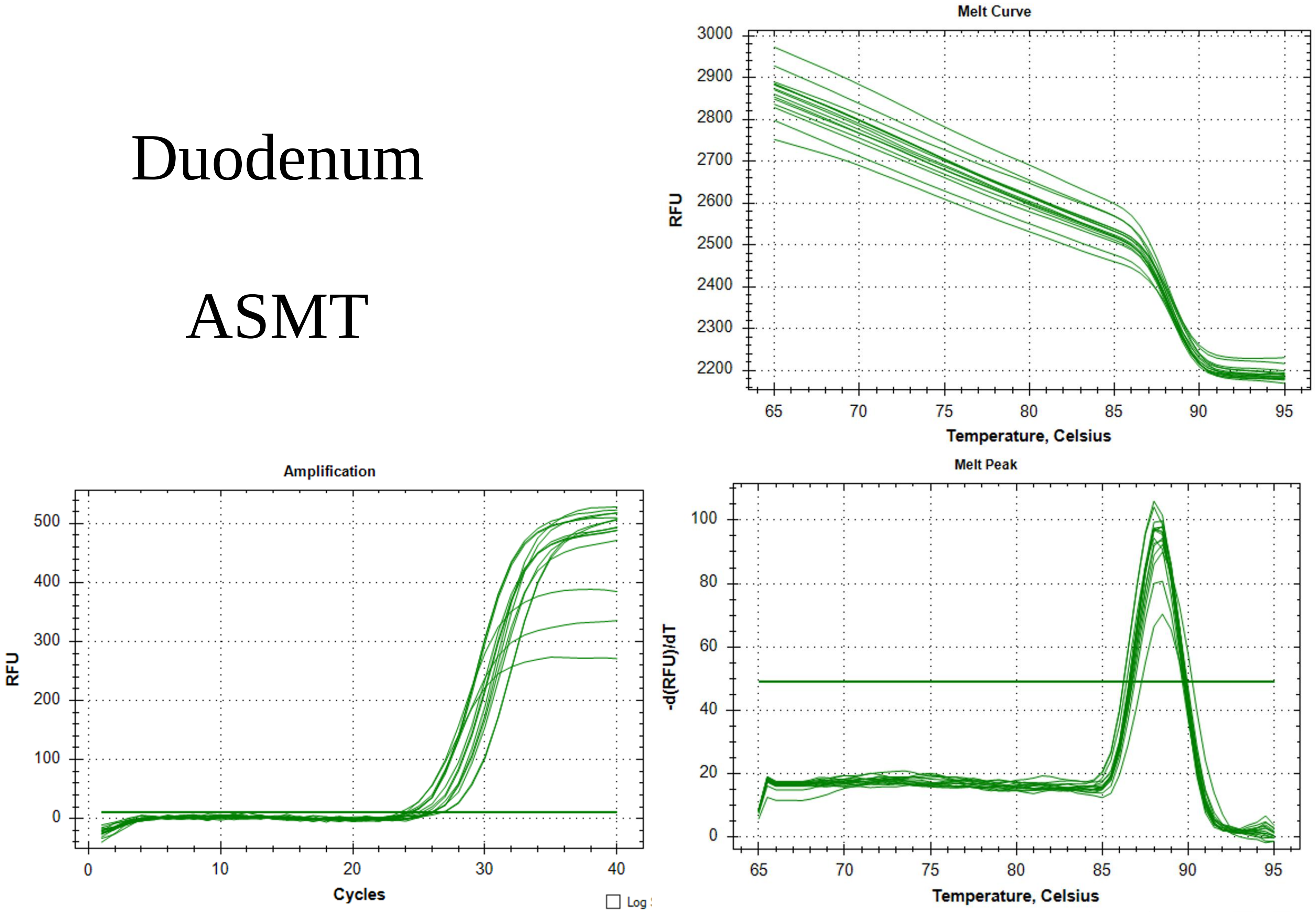

Endometrium

ASMT

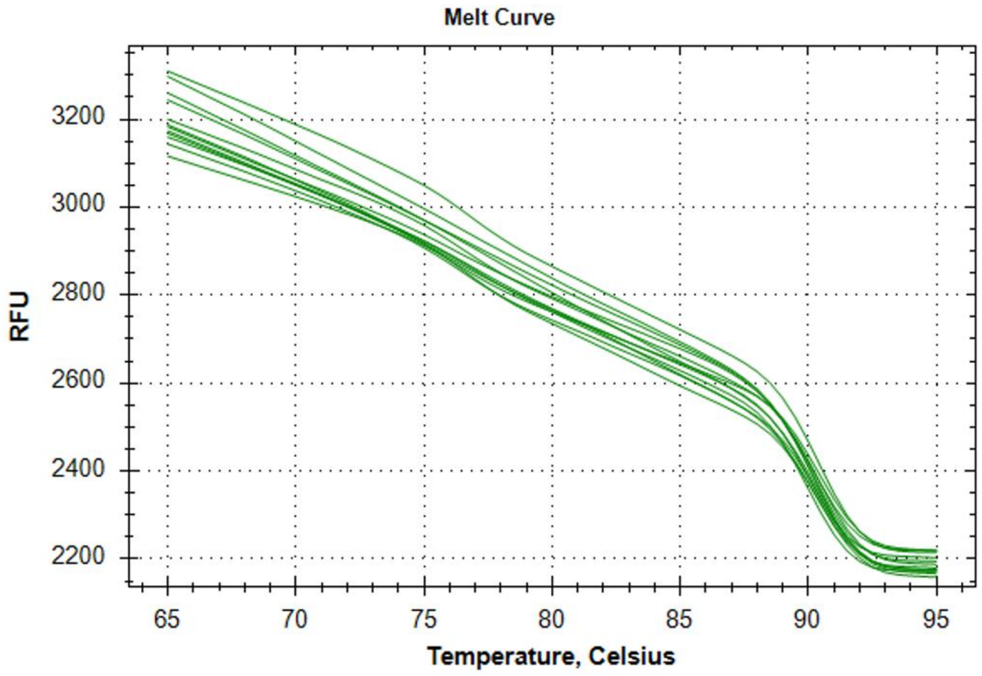

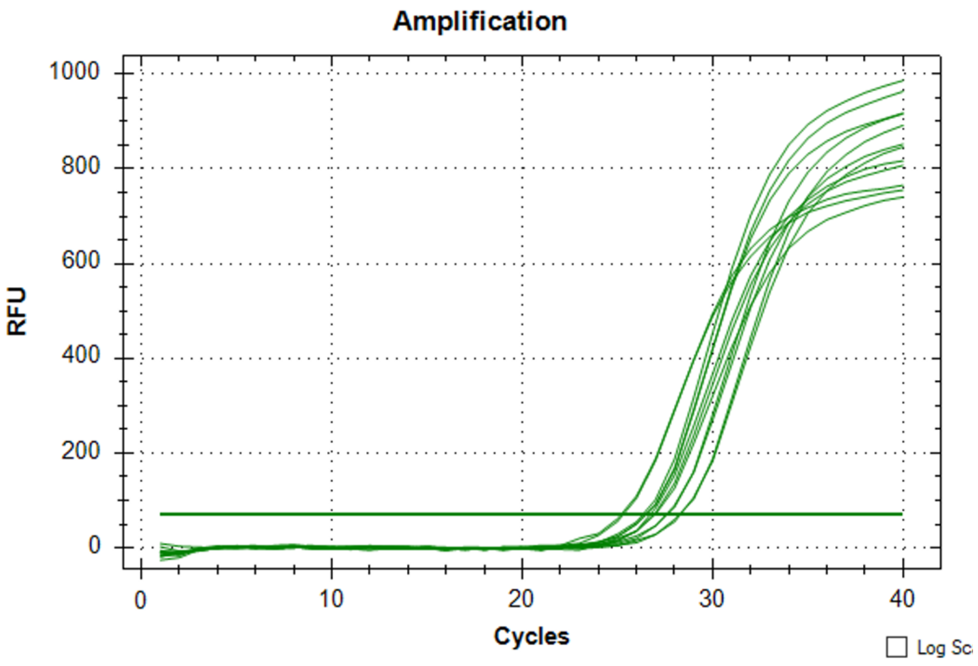

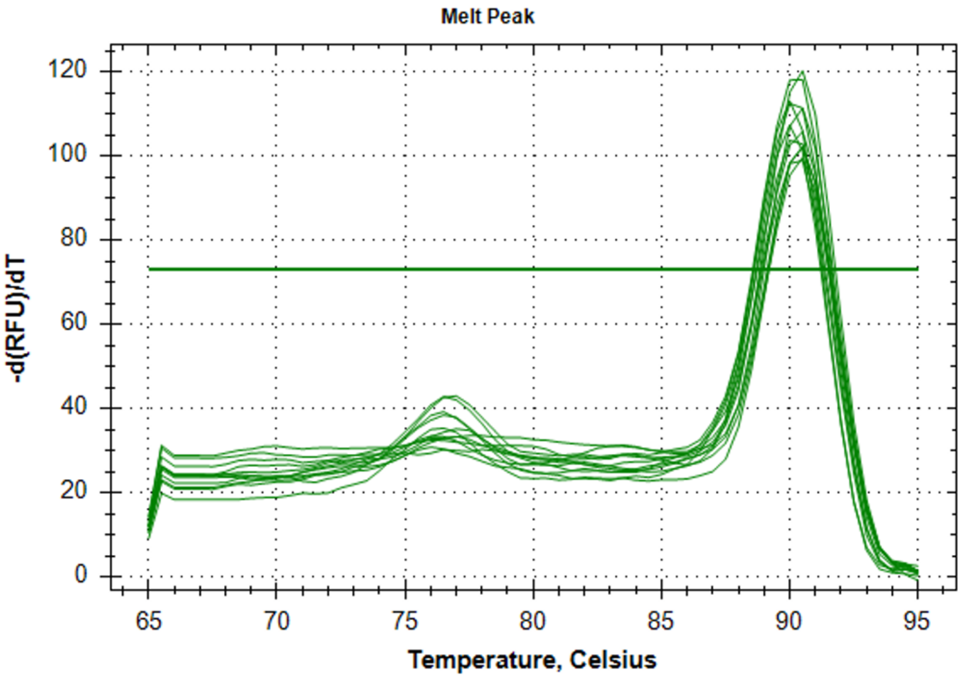

Supplement: Supplementary file 1 [file biomolecules-16-01047-s001.zip › Figure S2 Melt curve and amplification.pdf]
